# Modulation of Antitumor Immunity and the Tumor Microenvironment by *Scutellaria barbata* D. Don: A Scoping Review and Network Pharmacology Analysis

**DOI:** 10.3390/ijms27188419

**Published:** 2026-09-21

**Authors:** Soo-Dam Kim, Jung Hyang Park, Dong-Hyeon Kim, Taekyung Yeo, Mi-Jeong Choi, So-Jung Park, Hwa-Seung Yoo

**Affiliations:** 1 Department of Integrative Medicine, JABA Graduate School, Wonkwang University, Iksan 54538, Republic of Korea; hidden425@wku.ac.kr; 2 East West Cancer Center, Daejeon Korean Medicine Hospital, Daejeon University, Daejeon 34520, Republic of Korea; wjdgidqkr@naver.com; 3 Korean Medicine Hospital, Pusan National University, Yangsan 50612, Republic of Korea; dongxian92@gmail.com; 4 Oculi Korean Medicine Hospital, Seoul 05655, Republic of Korea; oculi2022@naver.com; 5 Biomedical Biotechnology Research Institute Co., Ltd., 32 Dongguk-ro, Ilsandong-gu, Goyang-si 10326, Republic of Korea; choimijeong5@gmail.com; 6 Department of Korean Internal Medicine, School of Korean Medicine, Pusan National University, Yangsan 50612, Republic of Korea

**Keywords:** *Scutellaria barbata*, tumor microenvironment, immunomodulation, network pharmacology, herbal medicine

## Abstract

*Scutellaria barbata* D. Don (SBD) has been widely investigated for antitumor activity, but its immune- and tumor microenvironment (TME)-related mechanisms remain fragmented. This study synthesized preclinical evidence on SBD and contextualized the findings using network pharmacology. A scoping review was conducted according to established scoping review methodology and PRISMA-ScR guidance. Preclinical in vitro and in vivo cancer studies published between January 2016 and December 2025 were searched in PubMed, Embase, CENTRAL, and OASIS. Network pharmacology analysis was performed using TCMSP, SwissTargetPrediction, ImmPort, GeneCards, STRING, Cytoscape, MCODE, DAVID, GO, and KEGG analyses. Fifty-six preclinical studies were included. SBD extracts, fractions, polysaccharides, diterpenoids, flavonoids, and isolated constituents suppressed cancer cell proliferation, induced cell-cycle arrest, promoted apoptosis and ferroptosis, modulated autophagy, induced endoplasmic reticulum stress-associated cell death, inhibited migration, invasion, angiogenesis, and metastasis, and enhanced chemosensitivity across diverse cancer models. Immune- and TME-related effects included activation of natural killer cells and CD8^+^ T cells, reduction in regulatory T cells, T helper 17 cells, myeloid-derived suppressor cells, and M2-like tumor-associated macrophage polarization, modulation of cytokine and inflammatory signaling, and regulation of epithelial–mesenchymal transition, matrix remodeling, angiogenesis, and metastatic niche formation. Network pharmacology identified 32 active candidate compounds, 408 predicted targets, and 152 immune/TME-overlapping targets. Protein–protein interaction and enrichment analyses highlighted AKT1, TNF, SRC, EGFR, and BCL2 as candidate hub targets and implicated PI3K-Akt, MAPK, Ras, EGFR tyrosine kinase inhibitor resistance, and proteoglycans in cancer. SBD-derived interventions showed antitumor, immunomodulatory, and TME-remodeling activities across diverse preclinical models. The integrated findings identify recurrent biological processes and candidate molecular targets, while differences in preparation, chemical characterization, dose, and experimental design limit direct comparison across studies. This framework supports further mechanistic, pharmacokinetic, and translational evaluation of chemically characterized SBD preparations and candidate compounds.

## 1. Introduction

Cancer remains a leading cause of morbidity and mortality worldwide, with an estimated 20 million new cases and 9.7 million deaths recorded in 2022 alone [1]. Despite the transformative impact of immune checkpoint inhibitors and other immuno-oncology strategies over the past decade, durable responses remain limited to a subset of patients with solid tumors [2]. This therapeutic gap is increasingly attributed to the biological complexity of the tumor microenvironment (TME), a dynamic milieu comprising immune cells, stromal components, vascular networks, and extracellular matrix that collectively regulate tumor progression, immune evasion, and treatment resistance [3,4]. Within the TME, suppressive immune populations such as regulatory T cells (Tregs) and tumor-associated macrophages (TAMs), along with pro-tumorigenic cytokine networks involving IL-6, IL-10, and TGF-β, represent critical mediators of resistance to both conventional and immunotherapy-based treatments [5,6]. These observations underscore the need for therapeutic strategies capable of simultaneously modulating multiple regulatory nodes within the tumor ecosystem.

Multi-component botanical medicines have attracted growing interest as candidates for such multi-target modulation [7,8]. *Scutellaria barbata* D. Don (SBD; Ban Zhi Lian), a perennial herb widely used in East Asian traditional medicine for its heat-clearing and detoxifying properties, has demonstrated consistent antitumor activity across a range of experimental systems [9,10]. SBD contains diverse bioactive constituents—including flavonoids (baicalein, baicalin, wogonin, luteolin, scutellarein), neoclerodane diterpenoids, and polysaccharides—which have individually been reported to exert antiproliferative, pro-apoptotic, and anti-invasive effects in various cancer cell lines [9]. In tumor-bearing animal models, SBD extracts have been shown to suppress tumor growth while modulating immune cell populations, including NK cells and Treg/Th17 ratios, and reducing immunosuppressive cytokines such as IL-10 and TGF-β [11]. These observations suggest that SBD may exert its anticancer effects not only through direct cytotoxicity but also through modulation of the tumor immune microenvironment.

Despite this growing body of evidence, the current understanding of SBD’s antitumor mechanisms remains fragmented. Existing preclinical studies have provided valuable evidence for the anticancer effects of SBD in diverse tumor models, but most have focused on individual cancer types, specific experimental systems, or discrete mechanistic endpoints, such as PI3K/AKT inhibition, caspase-mediated apoptosis, suppression of migration and invasion, or chemosensitization [12,13,13,14,15]. In parallel, network pharmacology-based studies have explored the potential anticancer mechanisms of SBD or its constituents in specific cancers, including hepatocellular carcinoma, thyroid cancer, and colorectal cancer [16,17,18]. However, these studies remain largely cancer type-specific and have not systematically clarified whether SBD regulates common biological processes across diverse tumor contexts, particularly those related to immune regulation and TME remodeling. Moreover, heterogeneity in SBD preparations, dosing conditions, and experimental models, together with limited pharmacokinetic and causal validation, complicates cross-study comparison and translation to clinical practice.

To address these limitations, we conducted a scoping review of preclinical studies to map the antitumor, immune-, and TME-related effects of SBD across diverse tumor models. We then performed a targeted network pharmacology analysis focused on immune- and TME-associated molecular targets and pathways to support systems-level interpretation of the mapped evidence. This integrated approach aims to identify conserved regulatory mechanisms underlying the antitumor activity of SBD and to facilitate hypothesis generation for future biomarker- or immunotherapy-oriented evaluation.

## 2. Materials and Methods

### 2.1. Study Design

This study was designed to systematically investigate the anticancer mechanisms of SBD, with a particular focus on immune regulation and TME-associated processes. The study consisted of two complementary components. First, a scoping review was conducted to comprehensively identify, map, and synthesize preclinical evidence related to the antitumor, immunomodulatory, and TME-associated effects of SBD. This review aimed to characterize experimental models, intervention types, and biological outcomes reported across studies. Second, a network pharmacology analysis was performed to explore how active compounds of SBD interact with immune- and TME-related molecular targets at a systems level. By integrating findings from these two approaches, this study aimed to provide a comprehensive interpretation of the molecular mechanisms underlying the anticancer activity of SBD within the tumor–immune–microenvironment context.

### 2.2. Scoping Review

The scoping review was carried out using the methodological framework originally described by Arksey and O’Malley, with reporting aligned to the PRISMA-ScR recommendations [19,20]. To enhance transparency and reproducibility, the review protocol was prospectively registered in the Open Science Framework (OSF) (https://osf.io/f3x4u, accessed on 20 September 2026). The methodological approach was further informed by guidance from the Joanna Briggs Institute for scoping reviews [21]. This review sought to provide a comprehensive synthesis of existing preclinical data on SBD, with a specific focus on three interconnected domains: direct antitumor activity, modulation of immune cell populations and cytokine networks, and remodeling of the TME. Included studies were examined to determine the range of cancer models employed, the nature of the interventions tested, and the mechanistic outcomes reported.

#### 2.2.1. Search Strategy

A systematic literature search was conducted in PubMed, Embase, CENTRAL, and OASIS. Studies published between January 2016 and December 2025 were included. The search strategy incorporated both controlled vocabulary and free-text keywords related to SBD and cancer. The main search terms included “*Scutellaria barbata*” and “Ban Zhi Lian,” along with cancer-related terms such as “cancer,” “tumor,” “carcinoma,” and “neoplasm.” These terms were combined using database-specific Boolean operators. No language restrictions were applied at the search stage; however, studies published in languages other than English were subsequently excluded during the eligibility screening process (Supplementary Table S1).

#### 2.2.2. Eligibility Criteria

Studies were included if they satisfied the following criteria: (1) preclinical research employing in vitro or in vivo cancer models; (2) administration of SBD-derived preparations, including crude extracts, fractionated components, or chemically identified active constituents; (3) reported outcomes demonstrating antitumor activity or biological effects associated with immune regulation or TME modulation. Studies were excluded if they involved multi-herb formulations in which the specific effects of SBD could not be clearly distinguished. In accordance with the registered protocol, review articles, editorials, conference abstracts lacking sufficient data for extraction, clinical investigations, and non-English-language full-text publications were excluded throughout the review process.

#### 2.2.3. Study Selection

Retrieved records were deduplicated and managed using EndNote 2025 (Clarivate Analytics, Philadelphia, PA, USA). Title and abstract screening was performed independently by two reviewers (S.D.K. and J.H.P.) to assess initial eligibility, followed by full-text evaluation of potentially relevant articles. Where disagreements arose between reviewers, resolution was achieved through structured discussion; cases that remained unresolved were adjudicated by a third reviewer (H.S.Y.). The complete selection workflow is summarized in a PRISMA-ScR flow diagram.

#### 2.2.4. Data Extraction and Synthesis

A predefined data extraction framework was applied to systematically gather information from the selected studies. The extracted variables comprised publication year, country of origin, cancer type, experimental model, type of SBD preparation, dosage and exposure parameters, evaluated outcomes, and key findings. The collected data were then structured to explore cross-study trends, including commonly reported mechanisms of action, shared signaling pathways, and recurrent biological effects. Consistent with the exploratory scope of a scoping review, a narrative synthesis approach was adopted rather than formal meta-analysis. Extracted findings were thematically organized by cancer type, experimental system, preparation type, and primary biological activity to facilitate cross-study comparison. For the narrative synthesis, interventions were categorized as crude and solvent extracts, enriched fractions and derived formulations, or isolated and chemically defined constituents.

### 2.3. Network Pharmacology Analysis

A network pharmacology framework was applied to elucidate the potential mechanisms by which bioactive constituents of SBD regulate immune responses and the TME at a systems level. This approach was based on the principles of network-oriented drug discovery and systems pharmacology in traditional medicine, aiming to capture multi-component, multi-target interactions rather than single-molecule effects [22,23]. This analysis was intended as a hypothesis-generating approach to propose candidate mechanisms, rather than to provide experimental validation. The analytical pipeline comprised sequential steps including compound screening, target prediction, integration of immune- and TME-related gene sets, network construction, and functional interpretation.

#### 2.3.1. Identification of Candidate Active Compounds

Candidate compounds of SBD were retrieved from the Traditional Chinese Medicine Systems Pharmacology Database (TCMSP) (https://tcmsp-e.com/tcmsp.php, accessed on 19 March 2026) [24]. Pharmacokinetic-related parameters, including oral bioavailability (OB) and drug-likeness (DL), were used to filter compounds with potential biological relevance. Compounds meeting the predefined thresholds of OB ≥ 30% and DL ≥ 0.18 were retained for subsequent analysis to balance pharmacokinetic plausibility with adequate compound coverage. In addition to the TCMSP-derived compounds, three bioactive compounds repeatedly reported in the included preclinical studies—scutellarein, scutellarin, and pheophorbide a—were manually added based on literature evidence. Scutellarein and scutellarin were excluded by the predefined OB/DL thresholds despite being registered in TCMSP, whereas pheophorbide a was not available in TCMSP.

#### 2.3.2. Target Prediction for Active Compounds

Putative human protein targets corresponding to the selected compounds were predicted using SwissTargetPrediction (https://www.swisstargetprediction.ch/, accessed on 19 March 2026) [25]. The SMILES structures of each compound were obtained from TCMSP or PubChem and submitted with the organism restricted to Homo sapiens. Targets with a predefined prediction probability of ≥0.09 were retained, while those below this threshold were excluded to reduce very low-probability predictions while maintaining sufficient target coverage for network construction. The resulting targets were consolidated into a unified list after removing redundant entries based on gene symbols or UniProt identifiers.

#### 2.3.3. Collection of Immune and TME-Related Gene Sets

Immune-associated genes were collected from the ImmPort database, which provides curated datasets linked to immune function [26]. Gene symbols from ImmPort immune-related gene lists were merged after removal of duplicates. Genes related to the TME were identified from the GeneCards platform using the keyword “tumor microenvironment”, and only genes with a relevance score ≥2 were selected to reduce weak keyword associations while retaining broad coverage of TME-related genes [27]. These datasets were integrated into a single gene pool after eliminating duplicate entries. Both databases were accessed on 19 March 2026.

#### 2.3.4. Identification of Intersecting Targets and PPI Network Construction

Overlap between compound-associated targets and the immune/TME gene set was determined to identify candidate genes potentially mediating the immunomodulatory and microenvironmental effects of SBD. These intersecting targets were subsequently analyzed using the STRING database (version 12.0, https://version-12-0.string-db.org/, accessed on 19 March 2026) to construct a protein–protein interaction (PPI) network, with the species restricted to Homo sapiens and an interaction confidence score ≥0.4, corresponding to the medium-confidence setting and balancing interaction reliability with network coverage [28]. The interaction data were exported for further analysis.

#### 2.3.5. Network Visualization and Identification of Key Targets

The PPI network was visualized and analyzed using Cytoscape software, version 3.10.4 (Cytoscape Consortium, San Diego, CA, USA) [29]. Topological parameters, including degree centrality and betweenness centrality, were calculated to identify hub genes and key regulatory nodes. In addition, the MCODE algorithm was applied to identify densely connected functional modules within the network [30].

#### 2.3.6. Functional Enrichment Analysis

To characterize the biological significance of the identified targets, functional enrichment analyses were performed using the DAVID bioinformatics platform. Gene Ontology (GO) biological processes, cellular component, and molecular function categories, as well as Kyoto Encyclopedia of Genes and Genomes (KEGG) pathways, were examined, and statistical significance was determined using adjusted *p*-values with Benjamini–Hochberg correction (FDR < 0.05) to control for multiple testing and reduce false-positive enrichment findings [31,32]. Enriched pathways were interpreted with particular attention to immune-related signaling, cytokine and chemokine networks, leukocyte activation, macrophage polarization, angiogenesis, and inflammatory regulation. Finally, the network pharmacology findings were integrated with preclinical evidence from the scoping review to propose a comprehensive mechanistic framework describing the role of SBD in modulating antitumor immunity and the TME.

## 3. Results

### 3.1. Study Selection Results

**Figure 1:**
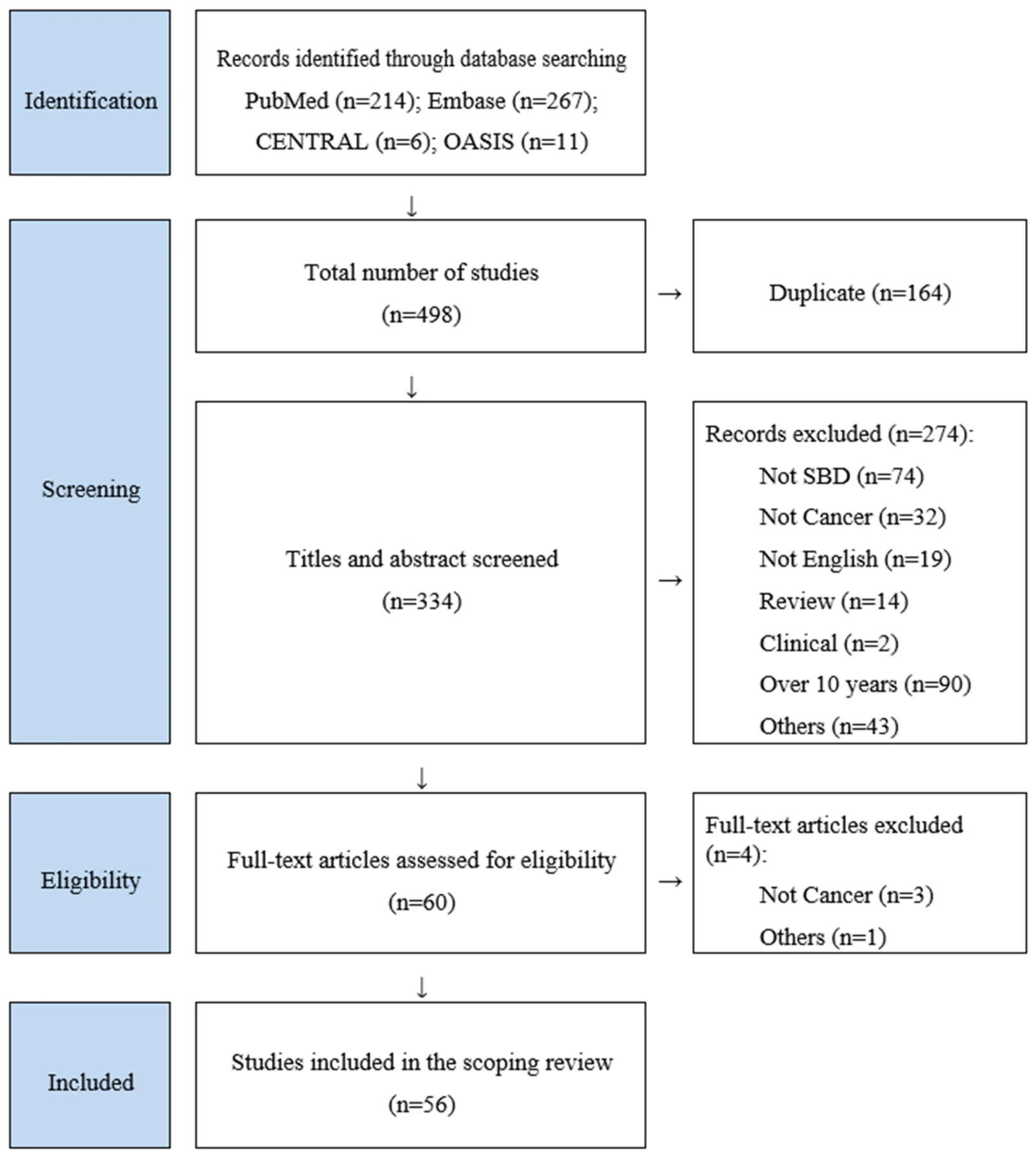
PRISMA-ScR flow diagram. SBD, *Scutellaria barbata* D. Don.

A total of 498 records were retrieved from the database search. After the removal of 164 duplicate entries, 334 studies remained and were subjected to title and abstract screening. Based on predefined eligibility criteria, a subset of articles was selected for full-text evaluation, resulting in 60 studies undergoing detailed assessment. Following full-text review, 56 studies were deemed eligible and included in the final analysis. Studies were excluded if they were not related to SBD, not relevant to cancer research, published in languages other than English, or categorized as review articles, conference abstracts, protocols, or other non-original research. The overall study selection process is illustrated in Figure 1.

### 3.2. Characteristics of Included Studies

The majority of the included studies were conducted in China (*n* = 52), with additional studies originating from Korea, Taiwan, the United States, and Vietnam. In terms of experimental design, in vitro-only studies accounted for the largest proportion (*n* = 31), followed by studies employing combined in vitro and in vivo approaches (*n* = 23), while only a limited number relied exclusively on in vivo animal models (*n* = 2).

A broad spectrum of cancer types was represented across the included studies. Colorectal cancer was the most frequently investigated (*n* = 16), followed by lung cancer, including non-small cell lung cancer and adenocarcinoma (*n* = 10), and hepatocellular carcinoma (*n* = 8). Additional cancer types included breast, gastric, bladder, pancreatic, osteosarcoma, ovarian, cervical, prostate, and hematologic malignancies, indicating that the antitumor effects of SBD have been explored across diverse oncological contexts.

The forms of intervention varied considerably and were grouped into three broad categories: (1) crude and solvent extracts, including aqueous, ethanol, methanol, petroleum ether, and chloroform preparations; (2) enriched fractions and derived formulations, including polysaccharide preparations, flavonoid and alkaloid fractions, oil preparations, and nanoparticle-based formulations; and (3) isolated or chemically defined constituents, such as scutellarin, scutellarein, quercetin, luteolin, baicalein, wogonin, and neo-clerodane diterpenoids. Crude and solvent extracts and enriched fractions were generally evaluated using mass-based concentrations, whereas isolated or chemically defined constituents were more commonly reported in molar units. In vivo studies employed oral or intraperitoneal administration over varying treatment periods, and conventional anticancer agents were used in some studies as positive controls, combination partners, or comparators in drug-resistance models. Quantitative phytochemical composition or marker-compound abundance was not consistently reported for crude extracts and enriched fractions. Detailed study-level information, including doses, exposure conditions, administration routes, treatment durations, and comparators, is provided in Supplementary Tables S2 and S3, while the main characteristics of the included studies are summarized in Table 1.

### 3.3. Antitumor Activity and Growth Inhibition

#### 3.3.1. Cancer Cell Proliferation and Cell-Cycle Regulation

Crude and solvent extracts of SBD suppressed cancer cell growth across diverse tumor models. Crude and aqueous extracts reduced cell viability, colony formation, EdU-positive proliferation, or tumor growth in hepatocellular, colorectal, lung, prostate, and ovarian cancer models [11,13,33,34,35]. Ethanol or methanol extracts showed growth-inhibitory effects in colorectal, gastric, lung, and ovarian cancer models [12,14,15,36,37,38,39,40], while petroleum ether and chloroform preparations were investigated in breast, cervical, and colorectal cancer models [41,42,43]. Enriched fractions and derived formulations also showed growth-inhibitory activity. Polysaccharide preparations reduced proliferation or tumor growth in colorectal and hepatocellular cancer models [44,45,46,47,48], flavonoid and alkaloid fractions showed similar effects in endometrial, ovarian, colorectal, and breast cancer models [49,50,51,52], and oil or nanoparticle-based formulations were evaluated in hepatocellular and pancreatic cancer models [53,54].

Several interventions showed selective or differential cytotoxicity. SBD water extract inhibited HCT116 and Colon26 cells without cytotoxicity in normal Hs27 fibroblasts [33]. Scutellarin inhibited SKOV3, A2780, and OVCAR8 ovarian cancer cells but not OVCAR3 cells [55]. BZL-5 reduced HCT-116 viability without significant toxicity to FHC normal colon epithelial cells [18], while SBD chloroform extract and crude extract showed selective effects in cervical and ovarian cancer models with limited toxicity to normal comparator cells [38,42]. In lung cancer models, SBD ethanol or methanol extracts reduced cancer cell viability, with methanol extract showing limited cytotoxicity toward normal MRC5 lung cells [37,40].

Isolated or chemically defined constituents also showed growth-inhibitory activity. Quercetin, scutellarin, rhamnazin, luteolin, baicalein, wogonin, and microbiota-derived SBD metabolites suppressed proliferation in gastric, esophageal, AML, colorectal, breast, hepatocellular, thyroid, nasopharyngeal, and ovarian cancer models [16,17,56,57,58,59,60,61,62,63]. Neo-clerodane diterpenoids showed compound-specific cytotoxicity across colorectal, breast, gastric, hepatocellular, and lung cancer cell lines, indicating structure-dependent antitumor potency [64,65,66,67,68,69,70].

Cell-cycle regulation was another recurrent mechanism. Interventions across these preparation categories induced G0/G1, G1, G2, or G2/M arrest with modulation of cyclin B1, cyclin D1, p-Cdc2, p-Cdc25C, p21, p27, p53, CDK4, and CDK1 [34,48,56,57,69,70,71,72]. Stemness-associated growth was also suppressed, as methanol extract of SBD reduced sphere formation and stemness-related gene expression in NSCLC through the SOX2/SMO/GLI1 loop, and scutellarin reduced breast cancer stem cell viability, sphere formation, CD44 expression, tumorigenicity, and lung metastatic potential [36,60]. Across preparation categories, reductions in cell viability and proliferation were generally concentration- or time-dependent. Detailed exposure conditions and reported IC_50_ values are provided in Supplementary Table S2.

**Table 1:** Characteristics of the included preclinical studies.

Study ID	Study Design	Cancer Type	Experimental Model	Intervention	Sample Size	Measured Outcomes
Bao 2019 [49]	In vitro	Endometrial cancer	Ishikawa human endometrial carcinoma cells co-cultured with U937 macrophage-derived TAMs	SBD flavonoids (SBF)	NR	1. Cell viability assay (MTT); 2. TAM marker expression analysis (flow cytometry for CD163, CD206); 3. Cytokine secretion analysis (ELISA for IL-10); 4. IL-12, IL-1β, TNF-α mRNA expression (qPCR); 5. Protein expression analysis (Western blot)
Cao 2025 [56]	In vitro + In vivo	Gastric cancer	HGC-27-HG gastric cancer cells under high-glucose conditions; mouse subcutaneous tumor xenograft model	Quercetin	NR	1. Cell viability assay (CCK-8); 2. Colony formation assay; 3. Apoptosis assay; 4. Cell cycle analysis; 5. Cell migration assay (wound healing); 6. Cell invasion assay (Transwell); 7. Protein expression analysis for PI3K/AKT signaling proteins (AKT1, TP53, JUN, MYC, CCND1) (Western blot); 8. Tumor growth measurement (mouse xenograft model)
Chen 2017 [40]	In vitro + in vivo	Liver cancer	HepG2 human hepatocellular carcinoma cells (in vitro) and H22 tumor-bearing mice (in vivo)	SBD methanol extract (MESB)	40 mice (4 groups, *n* = 10 each)	1. Cell viability assay (MTT); 2. Apoptosis assay; 3. Tumor growth measurement (tumor volume, mouse model); 4. Immune cell subset analysis (flow cytometry for NK cells, Treg cells, Th17 cells); 5. Cytokine level analysis (ELISA for IL-10, TGF-β, IL-17, IL-2, IFN-γ)
Chen 2021 [36]	In vitro + in vivo	Lung cancer	NSCLC cell lines and patient-derived xenograft (PDX) mouse model	SBD methanol extract (MESB)	NR	1. Cell viability assay (CCK-8); 2. Colony formation assay; 3. Sphere formation assay; 4. Stemness-related gene expression (RT-qPCR); 5. Target identification assays (DARTS, SPR); 6. Tumor growth measurement (PDX mouse model)
Chen 2024 [53]	In vitro	Liver cancer	SMMC-7721 human hepatoma cell line	SBD oil (SBO)	NR	1. Cell viability assay (SRB); 2. Cell viability assay (ATP-based assay, KG-1 cells); 3. Apoptosis morphology analysis (morphological observation); 4. Caspase-3 activity assay; 5. Apoptosis assay (TUNEL)
Ding 2025 [55]	In vitro	Ovarian cancer	Ovarian cancer cell lines (SKOV3, A2780, OVCAR3, OVCAR8) and patient-derived ovarian cancer organoids	Scutellarin (SCU)	NR	1. Cell proliferation assay; 2. Cell migration assay (Transwell); 3. Colony formation assay; 4. Calcein-AM/PI staining; 5. ATP assay for organoids; 6. Western blot: METTL5; 7. Database analysis (TCGA, GEPIA2, HPA, KM-plotter)
Feng 2021 [71]	In vitro	Liver cancer	Human HCC cell lines HepG2 and Huh7	Scutebarbatine A (SBT-A)	NR	1. Cell viability assay (CCK-8); 2. Colony formation assay; 3. Cell cycle analysis (flow cytometry); 4. Apoptosis assay (TUNEL); 5. Western blot: apoptosis and MAPK/ER stress proteins
Gao 2025 [50]	In vitro + in vivo	Ovarian cancer	SKOV3 human ovarian cancer cells; SKOV3 xenograft nude mouse model	SBD alkaloid fraction (SBA)	Not reported (mice *n* = 6/group reported)	1. Cell viability assay (CCK-8); 2. Colony formation assay; 3. Cell migration assay (wound healing); 4. Cell migration assay (Transwell); 5. Apoptosis assay (Annexin V/PI flow cytometry); 6. Western blot; 7. Xenograft tumor growth; 8. Histology
Guo 2019 [73]	In vitro	Colorectal cancer	HCT116 human colon cancer cells	Scutellarein	NR	1. Cell viability assay (MTT); 2. Apoptotic morphology (Hoechst staining); 3. DNA fragmentation assay; 4. ROS generation assay; 5. Mitochondrial membrane potential assay; 6. Caspase-3 activity assay; 7. Western blot: Bcl-2 and Bax
Hanh 2019 [64]	In vitro	Multiple cancers	LNCaP, HepG2, KB, MCF7, SK-Mel2 human cancer cell lines	Scutebarbatolides A–C and related diterpenoids (compounds 1–9)	NR	1. Cell viability assay (SRB)
Hu 2019 [37]	In vitro	Lung cancer	A549 human lung cancer cells	SBD ethanol extract (SBDE)	NR	1. Cell viability assay (MTT); 2. Cell morphology observation; 3. Cell growth curve analysis; 4. Apoptosis assay (Annexin V/PI flow cytometry); 5. Cell cycle analysis (flow cytometry); 6. Immunocytochemical staining: caspase-3, caspase-8, caspase-9, Ki-67, Bcl-2, p21ras
Huang 2022 [58]	In vitro + in vivo	Hematologic malignancies	HL-60, THP-1, and KG-1 AML cell lines; HL-60 xenograft nude mouse model	Scutellarin (SCU)	In vivo: *n* = 8 per group; in vitro repeated ≥3 times	1. Cell viability assay (CCK-8); 2. Apoptosis assay (Annexin V/PI flow cytometry); 3. Western blot: JNK, p-JNK, cleaved caspase-3, PARP; 4. Xenograft tumor volume and weight measurement
Jin 2017 [15]	In vitro	Colorectal cancer	HCT-8 human colorectal carcinoma cell line	SBD ethanol extract (EESB)	NR	1. Cell viability assay (MTT); 2. Cell migration assay (Transwell); 3. Invasion assay (Matrigel invasion chamber); 4. Western blot: MMP1, MMP2, MMP3/10, MMP9, MMP13, E-cadherin, N-cadherin, PI3K/AKT pathway proteins, TGF-β/Smad pathway proteins
Kan 2017 [11]	In vitro + in vivo	Liver cancer	HepG2 human hepatocellular carcinoma cells (in vitro) and H22 tumor-bearing BALB/c mice (in vivo)	SBD extract (SBE)	40 mice (4 groups, *n* = 10 each)	1. Cell viability assay (MTT); 2. Tumor volume measurement (3D ultrasound); 3. NK cell cytotoxicity assay; 4. Flow cytometry: Treg (CD4+CD25+Foxp3+) and Th17 (CD4+IL-17+) cells; 5. ELISA: TGF-β, IL-10, IL-17A, IL-2, IFN-γ
Li 2019 [45]	In vivo	Liver cancer	H22 murine hepatoma subcutaneous tumor-bearing Kunming mice	SBD polysaccharides (SBPS)	*n* = 10 per group (5 groups)	1. Tumor weight measurement; 2. Tumor inhibition rate analysis; 3. Serum proteomic profiling (2-DE and MALDI-TOF MS); 4. Differential protein expression analysis
Li 2019 [44]	In vitro + in vivo	Colorectal cancer	HT-29 colorectal cancer cells and splenic injection liver metastasis mouse model	SBD polysaccharide SBPW3	BALB/c nude mice (*n* = 8 per group) and HT-29 cells	1. Cell migration and invasion assays; 2. EMT marker analysis; 3. Liver metastasis nodule assessment in mice
Li 2022 [35]	In vitro + in vivo	Liver cancer	SMMC-7721, HepG2, and Huh7 hepatocellular carcinoma cell lines; nude mouse xenograft model	SBD crude preparation	In vivo: *n* = 5 mice per group; in vitro experiments repeated ≥3 times	1. Cell viability assay (CCK-8); cytotoxicity assay (LDH release); cell proliferation assay (EdU incorporation); 2. Iron concentration measurement; 3. Mitochondrial membrane potential assay (JC-1); 4. ROS generation assay (C11-BODIPY); 5. RT-PCR and Western blot: ferroptosis-related genes (GPX4, SLC7A11, IREB2, ACSL4); 6. Xenograft tumor volume and weight measurement; 7. Immunohistochemistry: Ki-67
Lin 2017 [12]	In vitro	Colorectal cancer	HCT-8/5-FU human colorectal cancer cell line	SBD ethanol extract (EESB)	NR	1. Cell viability assay (MTT); 2. Colony formation assay; 3. Apoptosis assay (DAPI staining, Annexin V/PI flow cytometry); 4. Drug efflux assay (Rhodamine-123 retention); 5. RT-PCR and Western blot: cyclin D1, p21, Bcl-2, Bax, ABCG2; 6. PI3K/AKT signaling analysis
Liu 2018 [74]	In vitro + in vivo	Lung cancer	H1975 NSCLC cells, RAW264.7 macrophages, and LLC tumor-bearing mouse model	Baicalein	C57BL/6 mice (*n* = 6 per group) and cultured cell lines	1. Cell viability assay (CCK-8); 2. Western blot: NF-κB, COX-2, iNOS, ERK, p38, AKT, CDK2, Bax, Bcl-2; 3. Tumor growth measurement; 4. Survival analysis; 5. Immune cell infiltration analysis (CD8+ T cells, NK cells)
Liu 2019 [57]	In vitro + in vivo	Esophageal cancer	ESCC cell lines and patient-derived xenograft (PDX) mouse model	Scutellarin (SCU)	NR	1. Cell proliferation assays; 2. Kinase binding assays; 3. Western blot; 4. Tumor volume measurement in PDX mice
Liu 2022 [51]	In vitro + in vivo	Colorectal cancer	HCT116 human colorectal cancer cells; mouse xenograft tumor model	SBD flavonoids (FSB)	NR	1. Cell viability assay (CCK-8); 2. Colony formation assay; 3. Migration assay; 4. Apoptosis assay; 5. Cell cycle analysis (flow cytometry); 6. Western blot and qRT-PCR; 7. Xenograft tumor growth measurement
Ma 2024 [60]	In vitro + in vivo	Breast cancer	MDA-MB-231 and MDA-MB-361 human breast cancer cells (breast cancer stem cells, BCSCs); mouse tumor-bearing model	Scutellarin (SCU) and SBD water extract (SBW)	NR	1. Cell viability assay; 2. Cell proliferation assay; 3. Mammosphere formation (self-renewal) assay; 4. Migration assay; 5. Molecular mechanism analysis; 6. Tumor growth assay in mice
Niu 2024 [70]	In vitro + in vivo	Gastric cancer	HGC-27 human gastric cancer cells cultured under high-glucose conditions; mouse xenograft tumor model using HGC-27-HG cells	SBD petroleum ether extract	NR	1. Cell viability assay (CCK-8); 2. Colony formation assay; 3. Apoptosis assay; 4. Cell cycle analysis; 5. Cell migration assay (wound healing); 6. Cell invasion assay (Transwell); 7. Western blot analysis; 8. Mouse subcutaneous tumor formation model
Niu 2025 [41]	In vitro + in vivo	Breast cancer	MCF-7 and MDA-MB-231 human breast cancer cells; mouse xenograft tumor model using MDA-MB-231 cells	Scutebarbatine B (SBT-B), a diterpenoid alkaloid isolated from SBD	NR	1. Cell viability (CellTiter-Glo assay); 2. EdU proliferation assay; 3. Cell migration assay (wound healing); 4. Cell invasion assay (Transwell); 5. Flow cytometry for cell cycle and apoptosis; 6. Apoptosis assay (TUNEL); 7. ROS generation assay (DCFH-DA, DHE); 8. Western blot analysis; 9. Mouse xenograft tumor growth measurement
Ouyang 2025 [17]	In vitro	Thyroid cancer	Human thyroid cancer cell lines	Quercetin (SBD component)	NR	1. Cell experiments
Shan 2025 [47]	In vitro + in vivo	Liver cancer	Human HepG2 hepatoma cells; BALB/c nude mice bearing HepG2 xenografts	SBD selenium-polysaccharide (SBP-Se)	NR	1. Cell viability assay (CCK-8); 2. Cell migration assay (Transwell); 3. Colony formation assay; 4. Tumor weight/body weight measurement; 5. H&E histology; 6. Western blot: PI3K/Akt/mTOR pathway
Sheng 2022 [13]	In vitro + in vivo	Prostate cancer	PC-3 and DU145 prostate cancer cell lines; 22RV1 xenograft mouse model	SBD extract	In vitro: experiments repeated ≥3 times; In vivo: *n* = 5 mice/group	1. Cell viability assay (CCK-8); 2. Apoptosis assay (Annexin V/PI flow cytometry); 3. Cell cycle analysis; 4. Colony formation assay; 5. Cell migration assay (wound healing, Transwell) and Cell invasion assay (Transwell); 6. Western blot: PI3K/AKT signaling and EMT markers; 7. Xenograft tumor volume and weight measurement; 8. Ki67 staining; 9. HUVEC tube formation assay
Shi 2019 [75]	In vitro + in vivo	Liver cancer	HepG2 and H22 hepatoma models	Scutellarein	NR	1. Cell proliferation assays; 2. Apoptosis analysis; 3. Tumor growth measurement; 4. Immune function indicator analysis
Shi 2022 [61]	In vitro	Nasopharyngeal cancer	Human nasopharyngeal carcinoma cell lines CNE2 and 5–8F	Baicalein, Wogonin	NR	1. Cell viability assay (CCK-8); 2. Apoptosis assay (Annexin V/PI flow cytometry); 3. Western blot: PI3K, AKT, and p53 pathway proteins
Shim 2016 [14]	In vitro	Gastric cancer	MKN-45 human gastric adenocarcinoma cell line	SBD ethanol extract (ESB)	NR	1. Cell viability assay (MTT); 2. Cell cycle analysis (sub-G1 population by PI flow cytometry); 3. Mitochondrial membrane potential assay (JC-1); 4. Caspase-3 and caspase-9 activity assays; 5. ROS generation assay; 6. MAPK pathway inhibition assays; 7. Chemosensitivity assays with anticancer drugs
Su 2022 [48]	In vitro + in vivo	Liver cancer	HepG2 cells; H22 tumor-bearing mice	SBD acidic polysaccharide SBP-2A	In vitro experiments repeated ≥3 times; In vivo: *n* = 10 mice per group	1. Cell viability assay (MTT, CCK-8); 2. Apoptosis assays; 3. Tumor weight measurement; 4. Serum proteomics analysis; 5. Histological examination
Sun 2017 [46]	In vitro	Colorectal cancer	HT29 human colon cancer cell line	SBD polysaccharide SPS2p	NR	1. Cell viability assay (MTT); 2. Apoptosis assay (Annexin V/PI flow cytometry); 3. Apoptosis index analysis (Hoechst staining); 4. Protein expression analysis (Bcl-2, Bax, Bak, p-AKT/AKT Western blot); 5. EMT marker analysis (E-cadherin, N-cadherin, vimentin qPCR); 6. Fibronectin expression analysis (ELISA)
Tan 2020 [76]	In vitro	Colorectal cancer	HCT116 human colorectal carcinoma cell line	Scutellarin (SCU)	NR	1. Cell viability assay (MTT); 2. Cell migration assay (wound healing); 3. Colony formation assay (soft agar); 4. Gene and protein expression analysis (qRT-PCR and Western blot for Hedgehog pathway components)
Wang 2018 [77]	In vitro	Lung cancer	A549, H460, H1650, H1975 human NSCLC cell lines	SBD and Long-Jin-San-Jie formula (LJSJF)	NR	1. Cell viability assay (MTT); 2. Colony formation assay (soft agar); 3. Gene and protein expression analysis (qRT-PCR and Western blot for EGFR, mTOR, p38 MAPK, cyclin D1, p53, p21, survivin, MDM2)
Wang 2019 [66]	In vitro	Multiple cancers	LoVo, MCF-7, SMMC-7721, HCT-116 human cancer cell lines	Neoclerodane diterpenoids (1–16)	NR	1. Cell viability assay (MTT)
Wang 2019 [54]	In vitro	Pancreatic cancer	PANC-1 human pancreatic cancer cell line	SBD-derived gold nanoparticles (AuNPs)	NR	1. Cytotoxicity assay (MTT); 2. Morphological assessment of apoptosis (acridine orange/propidium iodide staining); 3. Intracellular ROS generation assay (H2DCFDA staining/flow cytometry); 4. Western blot for apoptosis-related proteins (Bid, Bcl-2, Bax, β-actin); 5. RT-qPCR for apoptotic genes (Caspase3, Caspase9, Bax, Bid, Bcl-2)
Wang 2020 [65]	In vitro	Multiple cancers	LoVo (colon cancer), SMMC-7721 (hepatoma), HCT-116 (colon cancer), MCF-7 (breast cancer) cell lines	Neoclerodane diterpenoids (barbatin F, G, scutebata A–P)	NR	1. Cell viability assay (Methylene blue dye)
Wang 2024 [69]	In vitro + in vivo	Colorectal cancer	HT-29, HCT116, SW480 colorectal cancer cell lines; HT-29 xenograft nude mouse model	SBD diterpenoid Bzl-D	In vitro: experiments repeated ≥3 times; In vivo: *n* = 6 per group	1. Cell viability assay (CCK-8); 2. Apoptosis assay (Annexin V/PI flow cytometry); 3. Cell cycle analysis; 4. Migration assay (scratch assay); 5. Cell invasion assay (Transwell); 6. Western blot for HH pathway proteins (SHH, PTCH, SMO, GLI); 7. CETSA for SHH binding; 8. Tumor volume and weight in xenograft mice
Wang 2025 [78]	In vitro	Cervical cancer	Hela and SiHa cervical cancer cell lines	SBD active components baicalin and wogonin	NR	1. Cell viability assay (CCK-8); 2. Cell migration assay (wound healing); 3. Cell invasion assay (Transwell); 4. Apoptosis assay (flow cytometry); 5. Western blot for EMT and signaling pathway proteins
Wei 2017 [39]	In vitro + in vivo	Colorectal cancer	HT-29 human colorectal cancer cells and HT-29 xenograft BALB/c nude mouse model	SBD ethanol extract (EESB)	20 mice (10 EESB-treated, 10 control)	1. Tumor weight; 2. Ki-67 expression (IHC); 3. Cell viability assay (MTT); 4. Colony formation assay; 5. Gene expression analysis for c-Myc, Survivin, and APC (RT-PCR); 6. Protein expression analysis for β-catenin signaling (Western blot/IHC)
Xue 2016 [79]	In vitro	Breast cancer	MCF-7/ADR human breast cancer multidrug-resistant cell line	SBD-derived neoclerodane diterpenoids	NR	1. Cell viability assay (MTT); 2. Drug resistance reversal assay (IC50 of adriamycin); 3. Intracellular accumulation assay for ADR and Rh123 (flow cytometry); 4. P-glycoprotein expression analysis (Western blot)
Xue 2022 [42]	In vitro	Cervical cancer	Cervical cancer cell lines (HeLa, HT-3; comparison with normal HcerEpiC cells)	SBD chloroform extract (ECSB)	NR	1. Cell viability assay (CCK-8); 2. Apoptosis assay (TUNEL); 3. Cell migration assay (Transwell) and Cell invasion assay (Transwell); 4. Gene expression analysis for miR-195-5p and LOXL2 (qRT-PCR); 5. Protein expression analysis for LOXL2 (Western blot); 6. Dual-luciferase reporter assay
Yang 2017 [72]	In vitro	Colorectal cancer	HCT-116 human colon cancer cells	Scutellarin (SCU)	NR	1. Cell viability assay (MTT); 2. Hoechst 33342 staining; 3. AO/EB apoptosis staining; 4. Apoptosis assay (TUNEL); 5. Protein expression analysis for Bcl-2, Bax, caspase-3, p53, p21 (Western blot)
Yang 2017 [68]	In vitro	Multiple cancers	A549, MCF7, SGC7901 human cancer cell lines	SBD-derived neoclerodane diterpenoids, including scutebatas U–W	NR	1. Cell viability assay (MTT)
Yang 2018 [67]	In vitro	Multiple cancers	SGC-7901 (gastric), MCF-7 (breast), A-549 (lung) human cancer cell lines	Neoclerodane diterpenoids (scutebatas X–Z, A1–C1, compounds 1–6)	NR	1. Cell viability assay (MTT)
Yang 2022 [16]	In vitro	Liver cancer	Hepatocellular carcinoma cell lines (CCK-8 proliferation assay)	Rhamnazin and luteolin, active components of SBD	NR	1. Cell proliferation assay (CCK-8)
Yang 2025 [18]	In vitro	Colorectal cancer	HCT-116 colorectal cancer cells; FHC normal colon epithelial cells	BZL-5, a barbatin A diterpene isolated from SBD	NR	1. Cell viability assay (SRB); 2. Colony formation assay; 3. Cell migration assay (wound healing); 4. Cell invasion assay (Transwell); 5. Apoptosis assay (Annexin V/PI flow cytometry); 6. Gene expression analysis for STAT3, HSP90AA1, HIF1A, NFKB1, TLR4, PIK3CA (qRT-PCR)
Yang 2025 [80]	In vitro + in vivo	Liver cancer	In vitro: human HCC cell lines MHCC97-H and HuH-7; In vivo: HCC tumor xenograft mouse model (reported in study)	Luteolin (3′,4′,5,7-tetrahydroxyflavone), a flavonoid isolated from SBD	NR	1. Cell proliferation assay (CCK-8); 2. Cell migration assay (wound healing); 3. Cell invasion assay (Transwell); 4. EMT marker expression analysis (E-cadherin, β-catenin, Vimentin, N-cadherin, Snail, Twist, Slug); 5. Signaling pathway analysis (qRT-PCR, Western blot)
Yue 2021 [33]	In vivo	Colorectal cancer	HCT116 human colon cancer xenograft mice and murine colon26 tumor-bearing mice	SBD water extract (SBW) and scutellarin (SCU)	NR	1. Tumor weight; 2. Lung metastasis area; 3. Expression of metastasis-related proteins (E-cadherin, Tspan8, CXCR4, Src kinase)
Zeng 2021 [59]	In vitro + in vivo	Colorectal cancer	HT-29 colorectal cancer cells; AOM/DSS-induced CAC C57BL/6 mouse model	Scutellarin (SCU)	In vivo: mice randomized into 6 groups; In vitro: repeated ≥3 times	1. Cell viability assay (CCK-8); 2. Cell migration assay (wound healing); 3. Apoptosis assay; 4. ELISA for TNF-α and IL-6; 5. Western blot and qRT-PCR for Bax/Bcl-2 and Wnt/β-catenin pathway proteins; 6. Tumor pathology analysis
Zhang 2017 [38]	In vitro	Ovarian cancer	A2780 human ovarian cancer cells	SBD ethanol crude extract	NR	1. Cell viability assay (MTT); 2. Apoptosis assay (DAPI staining, Annexin V/PI flow cytometry); 3. Cell migration assay (wound healing); 4. Cell migration assay (Transwell); 5. Western blot for caspase-3, caspase-9, Bcl-2, MMP-2, MMP-9; 6. HPLC-Q-TOF-MS phytochemical analysis
Zhang 2017 [43]	In vitro	Colorectal cancer	HCT-8 human colon cancer cells	SBD chloroform fraction (ECSB)	NR	1. Cell viability assay (MTT); 2. Colony formation assay; 3. Apoptosis assay (Annexin V/PI flow cytometry); 4. qRT-PCR for miR-34a; 5. Western blot for Bcl-2, Notch1, Notch2, Jagged1
Zhang 2023 [62]	In vitro	Ovarian cancer	Human ovarian cancer SKOV3 cells	Quercetin, luteolin, and wogonin, active components of SBD	NR	1. Cell proliferation assay; 2. Colony formation assay; 3. mRNA expression analysis
Zhang 2024 [63]	In vitro	Liver cancer	HepG2 human hepatocellular carcinoma cells	Scutellarin, luteolin, and microbiota-derived metabolites	NR	1. Cell viability assay (CCK-8); 2. LC-MS/MS metabolite identification; 3. LC/MSn-IT-TOF analysis; 4. Enzyme metabolism assay
Zhang 2025 [34]	In vitro	Lung cancer	A549 lung cancer cell line	SBD aqueous extract (SBE), with wogonin as an active ingredient	NR	1. Cell viability assay (CCK-8); 2. Cell migration assay (wound healing); 3. Cell invasion assay (Transwell); 4. Protein expression analysis for CDK1 (Western blot)
Zheng 2018 [52]	In vitro + in vivo	Breast cancer	MDA-MB-231 breast cancer cells and nude mouse bone metastasis model	Total flavonoids of SB (TF-SB)	30 female BALB/c nude mice	1. Cell proliferation assay (MTT); 2. Cell migration assay (wound healing); 3. Cell invasion assay (Transwell); 4. Osteoclast number (TRAP staining); 5. PTHrP, RANKL, and OPG expression analysis; 6. Bone destruction analysis (micro-CT)

AKT, protein kinase B; AML, acute myeloid leukemia; BCSC, breast cancer stem cell; CAC, colitis-associated colorectal cancer; CRC, colorectal cancer; DARTS, drug affinity responsive target stability; EMT, epithelial–mesenchymal transition; ER stress, endoplasmic reticulum stress; ESCC, esophageal squamous cell carcinoma; HCC, hepatocellular carcinoma; HUVEC, human umbilical vein endothelial cell; MAPK, mitogen-activated protein kinase; NF-κB, nuclear factor kappa B; NK cells, natural killer cells; NR, not reported; NSCLC, non-small cell lung cancer; PDX, patient-derived xenograft; PI3K, phosphoinositide 3-kinase; ROS, reactive oxygen species; SPR, surface plasmon resonance; STAT3, signal transducer and activator of transcription 3; TAMs, tumor-associated macrophages; TGF-β, transforming growth factor-β; Th17 cells, T helper 17 cells; Treg cells, regulatory T cells.

#### 3.3.2. Induction of Apoptosis, Ferroptosis, Autophagy Modulation, and ER Stress-Associated Cell Death

Growth inhibition was closely linked to programmed cell death. SBD-based interventions induced apoptosis across multiple tumor models, as shown by nuclear condensation, Annexin V positivity, TUNEL staining, DNA fragmentation, caspase-3/8/9 activation, PARP cleavage, Bax or Bak upregulation, and Bcl-2 downregulation [13,14,35,37,38,40,41,42,43,48,50,51,53,54,56,58,59,70,71,72,73,74,75]. These effects were observed after treatment with crude extracts, ethanol extracts, methanol extracts, flavonoid fractions, alkaloid fractions, polysaccharides, SBD oil, isolated flavonoids, diterpenoids, and SBD-derived gold nanoparticles.

Mitochondrial and ROS-dependent apoptosis was frequently observed. Scutellarein induced mitochondrial ROS accumulation, cytochrome c release, caspase-3 activation, Bax upregulation, and Bcl-2 downregulation in multiple myeloma and HCT116 cells [73,75]. SBD ethanol extract induced ROS/MAPK-dependent apoptosis in MKN-45 cells, SBD-derived gold nanoparticles promoted mitochondria-mediated apoptosis in PANC-1 cells, and SBD oil activated caspase-8/9/3-mediated apoptosis in SMMC-7721 cells [14,53,54].

Other regulated death pathways were also reported. SBD extract induced ferroptosis-related changes in hepatocellular carcinoma cells, including iron accumulation, lipid ROS generation, GPX4 and SLC7A11 downregulation, and IREB2 and ACSL4 upregulation [35]. Scutebarbatine A activated PERK/ATF4/CHOP-mediated ER stress and MAPK signaling, while methanol extract of SBD induced ER stress, FAS/FASL signaling, mitochondrial dysfunction, and SIRT1 suppression in NSCLC cells [40,71]. SBD flavonoids also modulated autophagy through the ATF4/sestrin2 axis in colorectal cancer [51].

Apoptosis was the most consistently reported death-related outcome across preparation categories, whereas ferroptosis, autophagy-related modulation, and ER stress-associated cell death were each reported for a smaller number of specific preparations or constituents.

#### 3.3.3. In Vivo Tumor Growth Inhibition and Systemic Safety

In vivo studies supported the antitumor effects observed in vitro. SBD-based interventions reduced tumor volume, tumor weight, tumor burden, Ki-67 expression, or metastatic load in xenograft, syngeneic, orthotopic, patient-derived xenograft, and chemically induced models of hepatocellular carcinoma, colorectal cancer, NSCLC, breast cancer, ovarian cancer, esophageal cancer, prostate cancer, AML, and colitis-associated colorectal cancer [11,13,33,35,36,39,45,47,48,50,56,57,58,59,60,70,75].

Polysaccharide-based preparations reduced hepatoma growth, including SBD polysaccharides in H22 hepatoma-bearing mice and selenium-conjugated or acidic SBD polysaccharides in hepatoma models [45,47,48]. In colorectal cancer models, SBD water extract reduced xenograft and orthotopic tumor burden and lung metastatic burden, while ethanol extract of SBD reduced tumor weight and Ki-67 expression through Wnt/β-catenin suppression [33,39]. Scutellarin reduced tumor load and improved survival in AOM/DSS-induced colitis-associated colorectal cancer mice [59].

Isolated compounds and enriched fractions also showed in vivo activity. Scutellarin suppressed ESCC PDX, AML xenograft, and breast cancer stem cell-derived tumor growth [57,58,60]. Scutebarbatine B, quercetin, SBD alkaloid fraction, and SBD extract reduced tumor growth in breast, gastric, ovarian, and prostate cancer models [13,50,56,70]. Several studies reported stable body weight, unremarkable liver enzyme profiles, or no major organ toxicity at the tested doses [33,38,59,70]. Detailed in vivo dosing schedules and comparator conditions are provided in Supplementary Table S2.

#### 3.3.4. Inhibition of Migration, Invasion, Metastasis, and Tumor Vascularization

SBD and its constituents also inhibited migration, invasion, and metastatic behavior across colorectal, breast, ovarian, hepatocellular carcinoma, cervical, prostate, and lung cancer models [13,15,33,34,38,41,42,43,45,46,50,52,55,56,59,60,62,78,80]. These effects were associated with suppression of MMPs, EMT-related proteins, PI3K/AKT, TGF-β/Smad, RhoA/ROCK1-Ezrin, miR-6809-5p/FLOT1/FAK-PI3K-AKT-mTOR, and miR-195-5p/LOXL2 signaling [15,34,41,42,44,46,78,80].

In colorectal cancer models, SBPW3 reduced TGF-β1-induced migration and invasion and decreased liver metastatic nodules, while SBD water extract reduced lung metastatic burden in a Colon26-Luc orthotopic model [33,44]. Additional compound-specific effects included BZL-5-mediated inhibition of HCT-116 migration and wound healing, scutellarin-mediated suppression of HT-29 migration and anchorage-independent growth through Hedgehog signaling, and luteolin-mediated inhibition of HCC cell migration, invasion, and adhesion through the miR-6809-5p/FLOT1 axis [18,76,80]. In breast cancer models, SBD petroleum ether extract suppressed RhoA/ROCK1-Ezrin-mediated invasion, scutellarin reduced breast cancer stem cell migration and lung metastasis, and total SBD flavonoids attenuated bone metastasis-associated osteoclastogenesis through PTHrP/RANKL/OPG regulation [41,52,60].

Angiogenesis-related effects were also observed, as SBD extract inhibited HUVEC tube formation and prostate cancer angiogenesis via PI3K/AKT pathway inhibition, while cervical cancer experiments showed VEGFA downregulation alongside PI3K/AKT pathway suppression [13,78]. Collectively, these studies showed inhibition of cell motility, matrix degradation, EMT-associated signaling, angiogenesis, and metastatic niche formation across crude and solvent extracts, polysaccharide preparations, enriched fractions, and chemically defined constituents.

#### 3.3.5. Chemosensitization, Combination Effects, and Reversal of Drug Resistance

SBD-derived agents enhanced anticancer therapy or reversed drug resistance in several models. SBD ethanol extract increased the sensitivity of MKN-45 gastric cancer cells to cisplatin, etoposide, and doxorubicin through ROS/MAPK-dependent mechanisms [14]. Methanol extract of SBD enhanced etoposide- or cisplatin-induced effects in NSCLC cells, and SBD alkaloid fraction strengthened cisplatin-induced growth inhibition and apoptosis in ovarian cancer while attenuating cisplatin-induced renal toxicity in vivo [40,50]. SBD also enhanced anticancer effects when combined with other traditional medicine components in NSCLC cells, and scutellarein enhanced bortezomib-induced apoptosis in multiple myeloma models [75,77].

Drug resistance reversal was mainly associated with drug efflux inhibition. SBD ethanol extract overcame 5-FU resistance in HCT-8/5-FU colorectal cancer cells by suppressing ABCG2-mediated efflux and PI3K/AKT signaling [12]. Neo-clerodane diterpenoids reversed adriamycin resistance in MCF-7/ADR breast cancer cells by inhibiting P-glycoprotein activity and expression and increasing intracellular adriamycin and Rh123 accumulation [79]. Conventional anticancer drugs were used primarily as positive controls, combination partners, or comparators in drug-resistance models; no standardized head-to-head efficacy comparisons between SBD-derived interventions and standard anticancer drugs were identified (Supplementary Table S2).

### 3.4. Immune and Tumor Microenvironment Effects

#### 3.4.1. Activation of Antitumor Immunity and Effector Immune Cell Function

Evidence from preclinical studies indicates that SBD and its fractions modulate antitumor immunity by shifting the immune landscape toward an anti-suppressive and effector-dominant profile. In H22 hepatoma-bearing BALB/c mice treated with SBD extract, NK cell cytotoxicity was markedly increased, whereas CD4^+^CD25^+^Foxp3^+^ Treg cells and CD4^+^IL-17^+^ Th17 cells were reduced in tumor tissue. These immune changes were accompanied by increased serum IL-2 and IFN-γ and decreased IL-10, TGF-β, and IL-17A. Administration of IL-17A reversed the antitumor effects of SBD, directly supporting the functional importance of Th17/Treg balance in SBD-mediated antitumor immunity [11].

In LLC tumor-bearing mice, baicalein increased intratumoral CD8^+^ T-cell and NK-cell infiltration and decreased Treg cells, indicating enhancement of antitumor immune activity [74]. SBD water extract also decreased myeloid-derived suppressor cell (MDSC) populations in spleens of Colon26-Luc orthotopic tumor-bearing mice, with a similar decreasing trend observed in tumor tissues after scutellarin treatment [33]. Together, these findings indicate modulation of both lymphoid and myeloid compartments of the immunosuppressive TME, although findings other than the IL-17A reversal experiment should be interpreted as associative rather than causal.

#### 3.4.2. Modulation of TAM Polarization, Cytokine Networks, and Inflammatory Signaling

SBD consistently attenuated pro-tumorigenic inflammatory signaling across tumor models. In a co-culture system of Ishikawa endometrial cancer cells with U937 macrophage-derived TAMs, SBD flavonoids decreased CD163 and CD206 expression on TAMs, indicating inhibition of M2-like polarization. SBD flavonoids also reduced IL-10 secretion and decreased IL-12, IL-1β, and TNF-α mRNA expression, with the operative mechanism identified as blockade of the TLR4/MyD88/NF-κB pathway [49]. These findings suggest that SBD suppresses TAM activation and macrophage-associated immunosuppressive signaling in the TME.

Broader cytokine and inflammatory pathway modulation was reported across several studies. Scutellarin reduced serum TNF-α and IL-6 levels in AOM/DSS-induced colitis-associated colorectal cancer mice and simultaneously suppressed Wnt/β-catenin signaling in tumor tissue [59]. SBD oil inhibited IL-6-induced STAT3 activation and reduced p-STAT3 signaling in hepatoma cells [53]. In cervical cancer cells, SBD-derived components downregulated EGFR, TNF-α, VEGFA, PI3K, AKT, MDM2, and PI3K/AKT signaling [78]. Quercetin, luteolin, and wogonin decreased AKT1, VEGFA, JUN, and TNF mRNA expression and reduced PI3K/p-Akt protein levels while increasing caspase-3 expression [62]. These findings indicate that SBD-associated cytokine regulation is closely integrated with survival, apoptosis, angiogenesis, and metastatic signaling rather than representing an isolated anti-inflammatory effect.

Several computationally guided experimental studies also identified TME-relevant signaling nodes. BZL-5 decreased mRNA expression of STAT3, HSP90AA1, HIF1A, NFKB1, TLR4, and PIK3CA, with HIF-1 signaling proposed as a key pathway based on molecular docking and molecular dynamics simulation [18]. Quercetin reduced SPP1 expression in thyroid cancer cells [17], while SBD aqueous extract reduced CDK1 protein expression in A549 lung adenocarcinoma cells in a study linking CDK1 to LUAD progression and prognosis [34]. Although these studies were partly informed by network pharmacology or bioinformatics, they experimentally linked SBD-related compounds to inflammatory, hypoxic, and cell-cycle-associated tumor progression pathways. Across preparation categories, flavonoid fractions were associated with TAM polarization and TLR4/MyD88/NF-κB signaling, SBD oil with IL-6/STAT3 regulation, and defined flavonoids and diterpenoids with PI3K/AKT-, HIF-1-, and inflammation-related signaling.

#### 3.4.3. EMT, Matrix Remodeling, Angiogenesis, and Metastatic Niche Regulation

SBD and its constituents regulated structural components of the TME, particularly EMT, extracellular matrix remodeling, angiogenesis, and metastatic niche formation. EMT inhibition was observed across gastric, ovarian, prostate, colorectal, cervical, breast, and hepatocellular carcinoma models. Quercetin increased E-cadherin and decreased vimentin, N-cadherin, Snail, p-PI3K, p-AKT, and MYC in high-glucose gastric cancer cells [56]. SBD alkaloid fraction increased E-cadherin and decreased N-cadherin, MMP2, and MMP9 in ovarian cancer models [50]. In prostate cancer, SBD extract increased E-cadherin, decreased N-cadherin and vimentin, and reduced HUVEC tube formation, indicating concurrent regulation of EMT and angiogenesis [13].

In colorectal cancer models, SBD preparations converged on PI3K/AKT, TGF-β/Smad, Wnt/β-catenin, and Hedgehog-related pathways. Ethanol extract of SBD decreased p-PI3K, PI3K, p-AKT, TGF-β, Smad2/3, and Smad4 [15]. SBPW3 reversed TGF-β1-induced EMT by increasing E-cadherin and decreasing N-cadherin and α-SMA, with suppression of Smad2/3 phosphorylation [44]. SPS2p increased E-cadherin and reduced N-cadherin, vimentin, fibronectin, and p-AKT/AKT in HT29 cells [46]. Scutellarin reduced β-catenin, TCF4, c-Myc, and cyclin D1 in colitis-associated colorectal cancer and HT-29 models [59]. SBD diterpenoids and scutellarin suppressed Hedgehog signaling components, including SHH, PTCH, SMO, GLI, Ptch1, Gli1, and c-Myc [69,76].

SBD also regulated metastatic niche- and plasticity-associated pathways. SBD petroleum ether extract inhibited the RhoA/ROCK1-Ezrin axis in triple-negative breast cancer cells, and luteolin suppressed HCC cell migration, invasion, and adhesion through the miR-6809-5p/FLOT1/FAK-PI3K-AKT-mTOR axis [41,80]. In breast cancer stem cells, scutellarin inhibited Wnt/β-catenin, NF-κB, and PTEN/Akt/mTOR signaling, accompanied by reduced stemness and metastatic potential [60]. Chloroform fraction of SBD regulated the miR-34a/Notch axis, and total SBD flavonoids modulated the PTHrP/RANKL/OPG axis in the bone metastasis microenvironment [43,52]. Across preparation categories, solvent extracts and flavonoid or alkaloid fractions were mainly associated with EMT and matrix-related signaling, polysaccharide preparations with EMT and metastatic niche regulation, and chemically defined flavonoids and diterpenoids with tumor-cell plasticity, invasion, and angiogenesis (Supplementary Table S3).

### 3.5. Network Pharmacology Analysis Results

#### 3.5.1. Identification of Active Compounds and Overlapping Immune/TME Targets

**Figure 2:**
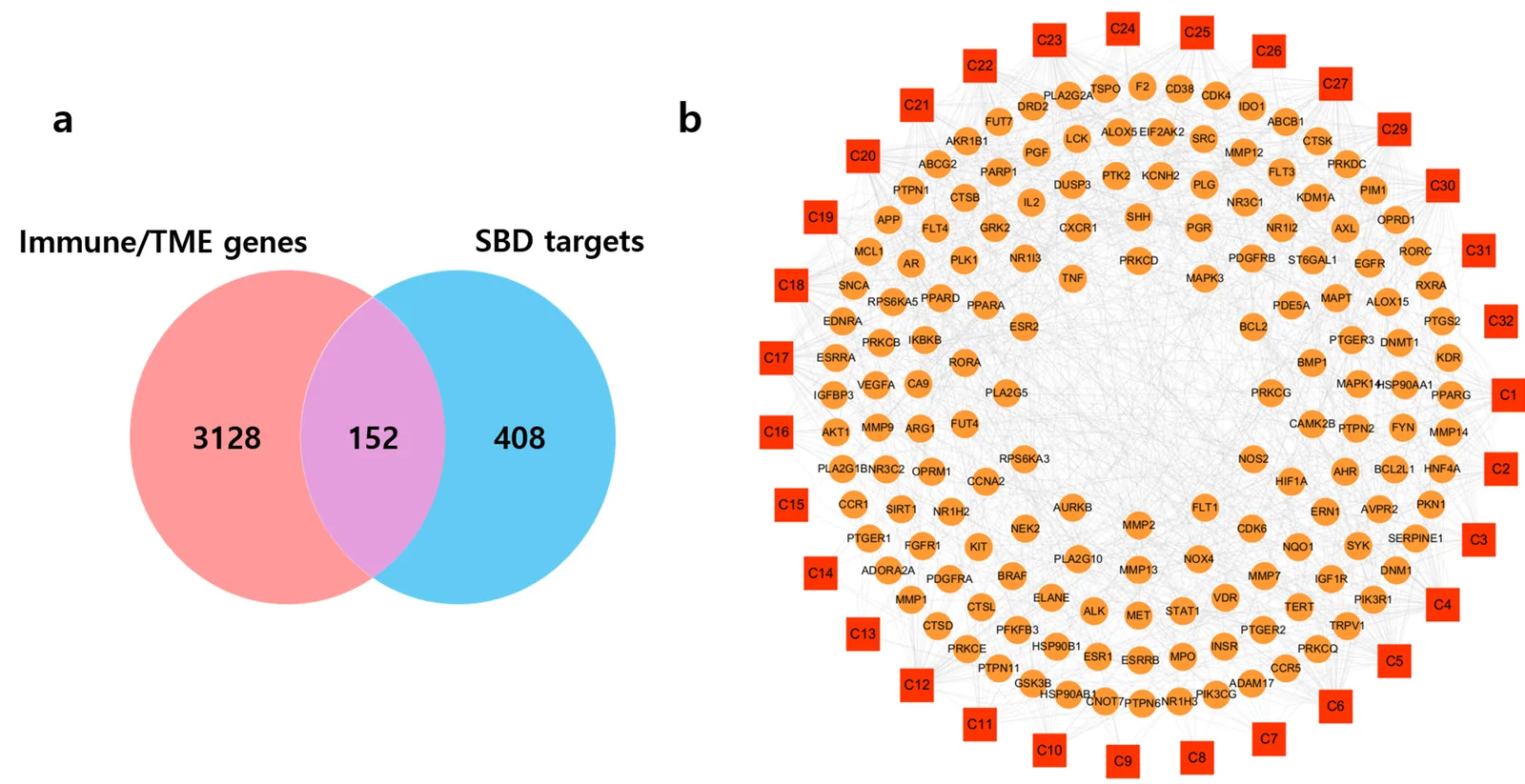
Compound–target network and identification of immune- and tumor microenvironment-related targets of *Scutellaria barbata*. (**a**) Overlap among 3128 immune/TME-related genes and 408 predicted SBD targets, yielding 152 intersecting targets. (**b**) Compound–target network of *Scutellaria barbata* showing active compounds and immune/TME-overlapping targets. Red squares represent active compounds, and orange circles represent immune/TME-overlapping targets. Gray edges indicate predicted compound–target associations, and node size is uniform and has no quantitative meaning. Compound identifiers C1–C32 correspond to the active candidate compounds listed in Supplementary Table S4.

A total of 3128 immune- and TME-related targets were collected from the ImmPort and GeneCards databases. From the TCMSP database, 94 compounds associated with SBD were initially retrieved, of which 29 met the predefined pharmacokinetic criteria of oral bioavailability (OB) ≥ 30% and drug-likeness (DL) ≥ 0.18. In addition, three literature-supported bioactive compounds were manually added, yielding 32 active candidate compounds for the final network pharmacology analysis. These compounds corresponded to 408 predicted protein targets, of which 152 overlapped with the immune- and TME-related gene set (Figure 2a). The relationships between active compounds and their predicted targets are shown in the compound-target network (Figure 2b). The compound identifiers C1–C32 and their corresponding compound names, major chemical classes, and SMILES representations are provided in Supplementary Table S4. Based on their major chemical scaffolds, the 32 candidate compounds were grouped into flavonoids (*n* = 20), sterol/triterpenoid derivatives (*n* = 11), and a chlorophyll-derived tetrapyrrole (*n* = 1). Key active compounds in this network included baicalein, baicalin, wogonin, luteolin, quercetin, scutellarein, and scutellarin from the flavonoid series, as well as β-sitosterol, stigmasterol, and campesterol from the phytosterol series. These compounds were connected to multiple immune- and TME-related targets, indicating a multi-compound and multi-target profile of SBD. Baicalein, wogonin, luteolin, quercetin, scutellarein, and scutellarin identified in the network analysis were also directly evaluated in the included preclinical studies.

#### 3.5.2. PPI Network Construction and Hub Target Identification

**Figure 3:**
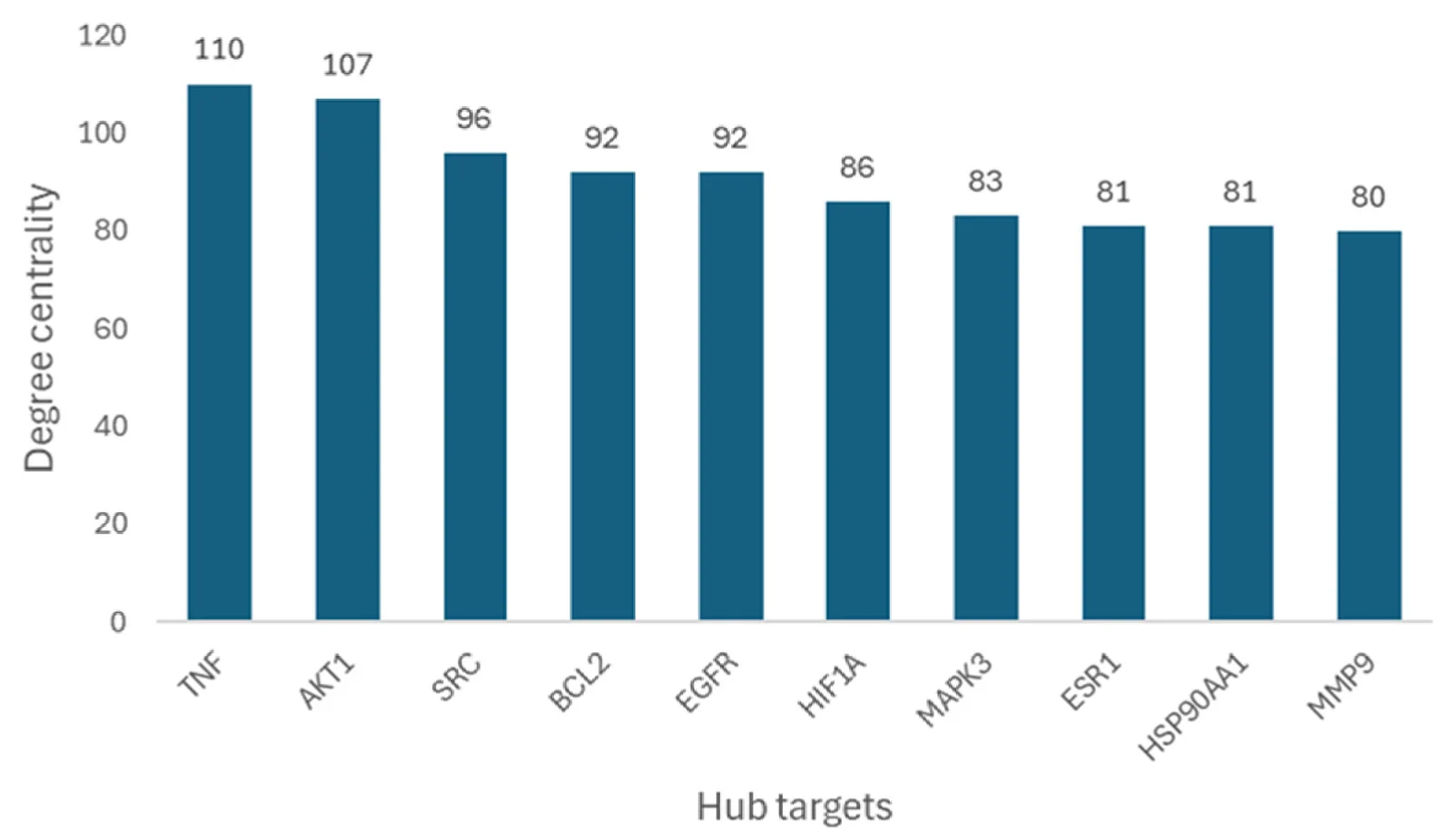
Hub-target ranking based on degree centrality in the complete PPI network. Degree centrality was calculated from the complete 152-node PPI network of immune/TME-overlapping SBD targets. The plot shows the top 10 highest-degree hub targets.

The 152 intersecting immune- and TME-related targets of SBD were used to construct a PPI network based on STRING analysis (Supplementary Figure S1). The final PPI network consisted of 152 nodes and 2196 edges, with an average node degree of 28.9 and a clustering coefficient of 0.606, indicating a highly interconnected network topology. The top 10 hub targets ranked by degree centrality calculated from the complete PPI network are presented in Figure 3. Topological analysis identified TNF, AKT1, SRC, EGFR, and BCL2 as the highest-degree hub targets. Additional high-degree targets included HIF1A, MAPK3, HSP90AA1, ESR1, and MMP9, suggesting involvement of hypoxia signaling, MAPK cascades, proteostasis, hormone-related signaling, and invasion-associated processes.

#### 3.5.3. Functional Module Analysis of the PPI Network

**Figure 4:**
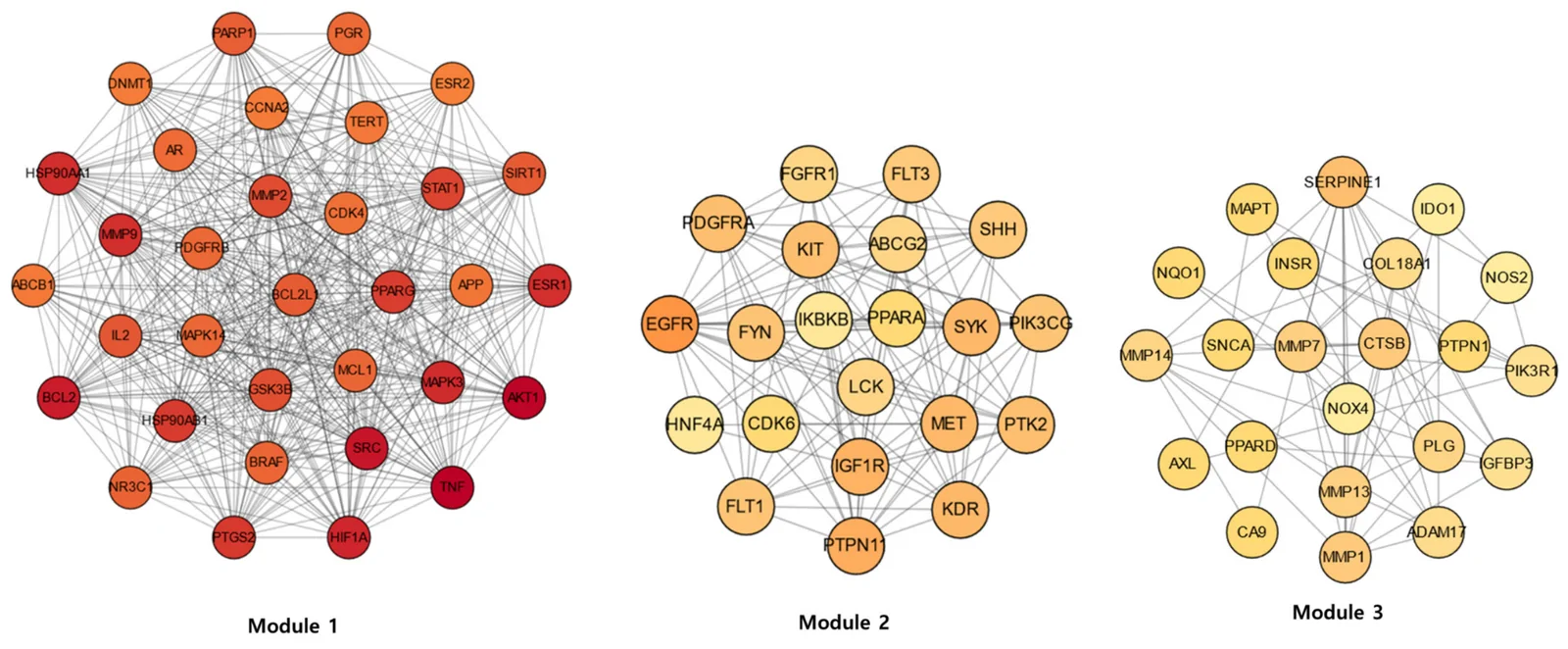
MCODE-based module clustering of the computationally constructed PPI network. Module 1: MCODE score = 27.562; Module 2: MCODE score = 10.7; and Module 3: MCODE score = 5.905. Node labels indicate gene symbols. Node size is uniform, and the yellow-to-red color gradient indicates increasing degree centrality. Gray edges represent protein–protein interactions within each module. MCODE, Molecular Complex Detection; PPI, protein–protein interaction.

Module analysis using the MCODE algorithm identified three highly interconnected clusters within the PPI network (Figure 4). Module 1 (MCODE score = 27.562) contained key regulators including AKT1, TNF, and BCL2, mainly associated with cell survival, apoptosis regulation, and inflammatory signaling. Module 2 (MCODE score = 10.7) comprised targets centered on EGFR, IGF1R, and MET, suggesting enrichment of growth factor receptor signaling and PI3K-related pathways. Module 3 (MCODE score = 5.905) included MMP family members and NOS2, indicating involvement in immune signaling, extracellular matrix remodeling, and inflammatory responses. The identified modules were associated with regulated cell death and survival signaling, growth-factor receptor pathways, inflammatory regulation, and extracellular matrix remodeling, consistent with the principal experimental domains identified in the scoping review.

#### 3.5.4. GO and KEGG Functional Enrichment Analysis

**Figure 5:**
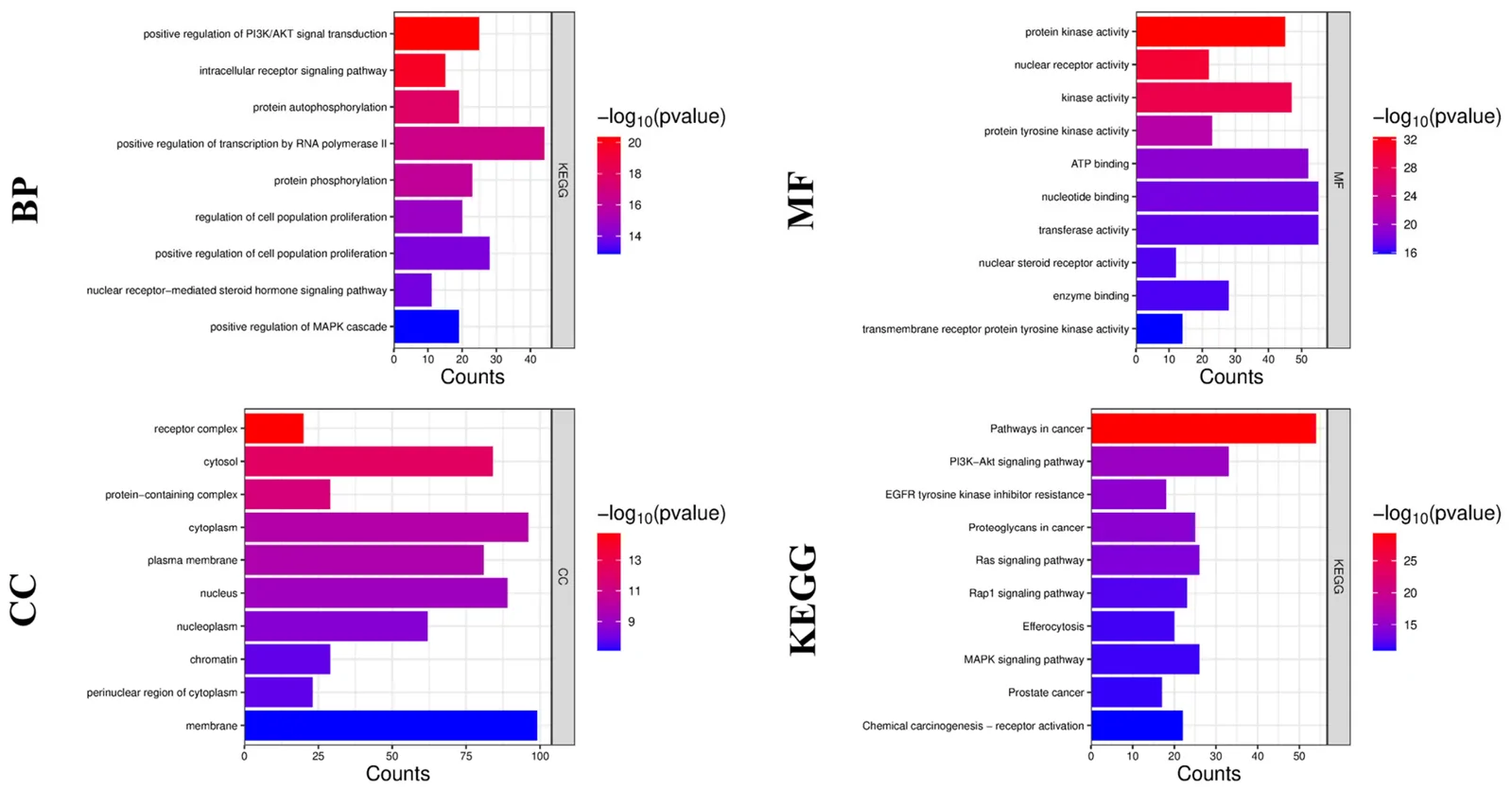
GO and KEGG enrichment analysis of the intersecting immune- and TME-related targets of *Scutellaria barbata*. The four panels present GO biological process, cellular component, and molecular function categories and KEGG pathway enrichment. The x-axis indicates the number of intersecting targets associated with each term, and bar color represents −log10 (adjusted *p*-value). The analysis primarily highlighted cancer-related pathways, PI3K–Akt and MAPK signaling, kinase activity, and membrane- and nucleus-associated components. Enrichment results are based on computational analysis of predicted targets rather than direct experimental validation. BP, biological process; CC, cellular component; MF, molecular function; GO, Gene Ontology; KEGG, Kyoto Encyclopedia of Genes and Genomes; TME, tumor microenvironment.

GO functional enrichment and KEGG pathway enrichment analyses were performed for the 152 intersecting targets using the DAVID database. GO analysis included biological process (BP), cellular component (CC), and molecular function (MF) categories, and the results are summarized in Figure 5.

In the BP category, the intersecting targets were enriched in apoptosis regulation, PI3K–AKT signaling, intracellular receptor signaling, protein phosphorylation, cell proliferation, and MAPK signaling. In the CC category, major enriched terms included plasma membrane, cytoplasm, nucleus, cytosol, receptor complex, chromatin, and nucleoplasm, suggesting involvement of both membrane-associated signaling compartments and nuclear regulatory structures. In the MF category, protein kinase activity, kinase activity, protein tyrosine kinase activity, nuclear receptor activity, ATP binding, and enzyme binding were predominant, indicating roles in signal transduction and transcriptional regulation.

KEGG pathway analysis showed enrichment in Pathways in cancer, PI3K–Akt signaling pathway, EGFR tyrosine kinase inhibitor resistance, Proteoglycans in cancer, and Ras signaling pathway. Additional enriched pathways included MAPK signaling, Rap1 signaling, Efferocytosis, Prostate cancer, and Chemical carcinogenesis-related pathways. The enriched pathways overlapped with recurrent experimental findings involving PI3K–Akt, MAPK, apoptosis, EGFR-associated signaling, drug resistance, and matrix remodeling.

#### 3.5.5. Cross-Tabulation of Network Predictions with Experimental Evidence

Cross-tabulation showed experimental overlap for eight of the top 10 PPI hub targets and direct evaluation of eight of the 32 network-derived candidate compounds (Supplementary Table S5). These overlaps indicate partial concordance between the experimental and computational findings rather than experimental validation of the network predictions.

## 4. Discussion

This study integrates a preclinical scoping review with network pharmacology analysis to clarify how SBD exerts antitumor effects through both tumor-intrinsic and TME mechanisms. Contemporary cancer biology increasingly views tumors as dynamic ecosystems in which malignant cells, immune populations, stromal elements, and vascular structures co-evolve to support tumor progression, immune evasion, and therapeutic resistance [81]. Although previous reviews have summarized the phytochemical composition, antitumor mechanisms, clinical application, and broad pharmacological activities of SBD [9,82], its immune- and TME-related anticancer mechanisms have not been systematically organized within a systems-level framework. The present synthesis is consistent with these reviews in identifying growth inhibition, apoptosis, and PI3K/AKT-related signaling as recurrent themes, but extends the existing literature by organizing immune- and TME-related evidence across experimental models and preparation types. By combining empirical evidence with computational target mapping, the present work frames SBD as a multi-component modulator of the tumor ecosystem rather than a single-pathway cytotoxic agent. Network-based analyses were used to contextualize, rather than replace, the experimentally derived findings [22].

Across preparation categories and tumor types, growth inhibition and regulated cell death were the most consistently reported effects of SBD-derived interventions [13,14,35,37,38,40,41,42,43,48,50,51,53,54,56,57,58,59,70,71,72,73,74,75]. Evidence from isolated constituents was compound-specific, whereas findings for crude extracts and enriched fractions reflected chemically broader preparations with variable levels of phytochemical characterization. Several studies also reported limited cytotoxicity in normal comparator cells, suggesting selective or differential antitumor activity in specific experimental contexts [18,33,38,40,42,55]. These phenotypes were accompanied by modulation of cyclins, CDK-associated proteins, p53/p21 signaling, and Bcl-2 family members, suggesting interference with survival and stress-response programs at multiple points [34,41,48,56,57,69,71,72]. Among these effects, growth inhibition and apoptosis were the most consistently reproduced across preparations and models. In contrast, ferroptosis, autophagy-related modulation, and ER stress-associated cell death were supported by fewer studies, and several pathway assignments were based primarily on molecular marker changes; stronger causal support was limited to studies using pharmacological inhibition, rescue experiments, target perturbation, or direct binding assays [11,14,36,41,43,51,55,57,58,70,71,72,75,80].

The network analysis helps contextualize this diversity. Intersecting immune/TME targets clustered around hub genes including AKT1, TNF, SRC, EGFR, and BCL2 within a dense PPI network comprising 152 nodes and 2196 edges, with a clustering coefficient of 0.606. KEGG enrichment of cancer-associated pathways, PI3K-Akt signaling, EGFR tyrosine kinase inhibitor resistance, and Ras signaling further suggests that SBD-associated compounds converge on signaling networks involved in tumor survival and therapeutic resistance. Several network-derived candidate compounds, including baicalein, wogonin, luteolin, quercetin, scutellarein, and scutellarin, were also directly evaluated in the included preclinical studies. In addition, the major network modules broadly corresponded to recurrent experimental domains involving survival and inflammatory signaling, growth-factor receptor pathways, and extracellular matrix remodeling. These network findings identify candidate mechanisms for further validation rather than confirming shared causal pathways across preparations or cancer types.

In vivo experiments extended the in vitro findings to tumor-level outcomes. SBD-based preparations reduced tumor volume, tumor weight, tumor burden, Ki-67 expression, or metastatic load across xenograft, syngeneic, orthotopic, patient-derived xenograft, and chemically induced models [11,13,33,35,36,39,47,48,50,56,58,59,60,75]. The interpretation of these findings differs according to model type. In vitro studies support detailed pathway investigation but do not reproduce systemic exposure or the full TME [55,71]. Conventional xenografts demonstrate in vivo tumor-growth effects but provide limited evidence for immune mechanisms [60]. Immunocompetent syngeneic and orthotopic models more directly reflect host immune and microenvironmental responses [11,33]. PDX models preserve aspects of human tumor heterogeneity but lack an intact human immune environment [36,57]. Chemically induced models represent inflammation-associated tumorigenesis within a specific disease context [59]. Several studies also reported stable body weight, unremarkable liver enzyme profiles, or no major organ toxicity, suggesting preliminary tolerability signals at the tested doses [33,38,41,59]. In addition, SBD-derived agents enhanced sensitivity to several chemotherapeutic agents, including cisplatin, etoposide, doxorubicin, 5-fluorouracil, and bortezomib, and some studies reported attenuation of cisplatin-induced renal toxicity [12,14,40,50,75,77,79]. Most studies used conventional anticancer drugs as positive controls, combination partners, or comparators in drug-resistance models rather than in standardized head-to-head efficacy comparisons. Accordingly, the available evidence more directly supports chemosensitization, drug-resistance modulation, and potential toxicity attenuation than equivalence or superiority to established anticancer drugs. In the network analysis, these observations aligned with the centrality of AKT1 and EGFR within PI3K-related and growth factor receptor-associated modules, which are closely linked to survival signaling and drug resistance [12,13,57,62].

The immune findings indicate that SBD not only affects tumor cells directly but also reshapes antitumor immune responses. Evidence for immune modulation was reported in both tumor-bearing animal models and macrophage–tumor cell co-culture systems. In vivo studies showed increased NK-cell cytotoxicity or CD8^+^ T-cell and NK-cell infiltration, together with reductions in Treg cells, Th17 cells, and MDSCs [11,33,74]. In a macrophage–tumor cell co-culture model, SBD flavonoids reduced M2-like TAM polarization [49]. These effects were accompanied by modulation of cytokines and inflammatory mediators, including increased IL-2 and IFN-γ and decreased IL-10, TGF-β, IL-17A, TNF-α, IL-6, COX-2, iNOS, and NF-κB-related signaling [11,49,59,74]. The reversal of the antitumor effect by IL-17A provided functional support for Th17/Treg-related immune modulation [11], whereas most other immune findings were based on changes in immune-cell populations or cytokine markers and require further causal validation. Although these effects were demonstrated mainly with SBD extracts, flavonoid preparations, and baicalein, the findings suggest coordinated modulation of lymphoid and myeloid components of the immunosuppressive TME. Network and pathway findings further linked SBD-related compounds to inflammatory and immune-relevant nodes, including TNF, AKT1, HIF1A, STAT3-related signaling, TLR4/NF-κB-related pathways, and PI3K-Akt signaling [18,49,53,62,74,78]. These observations provide a rationale for further evaluation of selected SBD-derived interventions in immunotherapy combination models [2].

Structural remodeling of the TME was another recurring theme. SBD and its constituents inhibited EMT, extracellular matrix remodeling, angiogenesis, and metastatic niche formation across multiple tumor models [15,42,44,46,50,56,70,78,80]. EMT reversal was associated with increased E-cadherin and decreased N-cadherin, vimentin, Snail, α-SMA, and MMPs, with involvement of PI3K/AKT, TGF-β/Smad, RhoA/ROCK1-Ezrin, miR-6809-5p/FLOT1/FAK-PI3K-AKT-mTOR, and miR-195-5p/LOXL2 signaling [15,44,46,55,56,70,78,80]. Stemness- and plasticity-associated pathways were also affected, including Wnt/β-catenin, Hedgehog, NF-κB/PTEN-Akt-mTOR, and miR-34a/Notch signaling [36,43,59,60,69,76]. Reported preparation-specific patterns included associations of solvent extracts and enriched flavonoid or alkaloid fractions with EMT and matrix-related signaling, polysaccharide preparations with EMT and metastatic niche regulation, and isolated flavonoids and diterpenoids with pathways governing invasion, angiogenesis, and tumor-cell plasticity. Although the recurrence of EMT-related marker changes supports an anti-invasive pattern, causal validation was limited to a smaller subset of studies using rescue or pathway-perturbation approaches [41,43,55,80]. Evidence for angiogenesis and metastatic niche regulation was less extensively replicated. One study further reported that gut microbiota-mediated biotransformation of SBD flavonoids generated metabolites with stronger antiproliferative activity than the parent compounds, suggesting that metabolism may influence the observed biological activity [63]. Network enrichment for Proteoglycans in cancer and the MMP/NOS2-associated module was consistent with the experimental findings related to extracellular matrix dynamics, inflammation, and tumor–stroma interactions.

The network analysis identified AKT1, TNF, SRC, EGFR, and BCL2 as central hubs and showed enrichment of PI3K–Akt signaling, EGFR tyrosine kinase inhibitor resistance, Ras signaling, and other cancer-related pathways. The compound–target network identified flavonoids including baicalein, baicalin, wogonin, luteolin, quercetin, scutellarein, and scutellarin, together with phytosterols such as β-sitosterol, stigmasterol, and campesterol, as candidate compounds connected to multiple immune- and TME-related targets. Several of these flavonoids were also directly evaluated in the included preclinical studies and showed antiproliferative, pro-apoptotic, anti-invasive, or immune/TME-modulating effects [16,17,56,57,58,60,61,62,63]. The three MCODE-derived modules involved AKT1/TNF/BCL2-centered survival and inflammatory signaling, EGFR/IGF1R/MET-centered growth-factor receptor signaling, and MMP/NOS2-associated matrix remodeling and inflammatory responses. Together, the experimental and computational findings identify recurrent biological domains involving growth control, apoptosis, PI3K/AKT and MAPK signaling, immune regulation, and matrix remodeling. However, network-derived compounds were not necessarily quantified in the preparations evaluated in the included studies; therefore, the predicted associations should not be interpreted as preparation-specific chemical profiles or confirmed mechanisms.

From a translational viewpoint, the integrated evidence suggests potential adjunctive roles for selected SBD-derived interventions targeting tumor survival, immune suppression, and microenvironmental remodeling. Reported effects on chemotherapeutic sensitivity, treatment-related toxicity, effector immune responses, and EMT- and matrix-associated pathways align with current efforts to overcome therapeutic resistance through multimodal intervention [11,12,14,33,40,44,49,50,52,70,74,75,79]. However, these translational implications remain preliminary because the evidence base is entirely preclinical and heterogeneous in terms of extract preparation, phytochemical characterization, dosage, exposure conditions, tumor models, and outcome measures. Quantitative information on the abundance of active constituents in the source plant material and tested extracts was infrequently reported, limiting attribution of extract-level activity to individual compounds and comparison among differently prepared extracts. Differences in composition, potency, bioavailability, and pharmacological mechanisms limit direct comparison across intervention categories, and findings from isolated constituents cannot be directly extrapolated to whole-herb preparations or generalized across different SBD preparation types. Accordingly, the current evidence cannot establish clinical efficacy, an optimal preparation or dose, clinically relevant herb–drug interactions, or long-term safety. Common preclinical reporting limitations, including incomplete reporting of sample size calculation, randomization, blinding, and formulation characterization, may also limit comparability across studies. The predominance of studies from China may introduce geographic and publication bias. Because Chinese-language databases such as CNKI, Wanfang, and VIP were not searched and non-English full texts were excluded, potentially relevant preclinical studies may have been missed, particularly given the concentration of SBD research in China. This language restriction may therefore limit the completeness and representativeness of the evidence base. Future multilingual reviews incorporating Chinese-language databases and bilingual screening would help provide a broader assessment of the preclinical literature.

As a scoping review, this study maps mechanistic breadth without providing pooled effect estimates or certainty-of-evidence grading. In addition, network pharmacology findings should be interpreted as hypothesis-generating because predicted compound-target interactions and enrichment results do not establish causal mechanisms [22]. Database-driven target prediction may also miss relevant interactions and does not fully account for exposure-related constraints such as bioavailability, metabolism, tissue distribution, or tumor-specific pharmacokinetics. The resulting networks are also sensitive to database coverage, predefined selection thresholds, prediction algorithms, and gene-set definitions, and the broad GeneCards-derived TME gene set may increase nonspecific overlap and network connectivity. Because the network component was centered on shared immune- and TME-related targets across heterogeneous tumor models, cancer-type-specific compound-target-disease pathway analyses were not performed.

Future mechanistic research should prioritize recurrent pathways supported by functional perturbation, particularly AKT-related survival signaling and ROS/MAPK-mediated apoptosis, whereas less-replicated findings involving ferroptosis, autophagy-related modulation, immune-cell remodeling, angiogenesis, and metastatic niche regulation require independent confirmation. Future studies should provide botanical authentication, extraction procedures and yields, quantitative marker-compound profiles, batch standardization, and complete dose, route, duration, and exposure information. They should also incorporate pharmacokinetic/pharmacodynamic assessment, mechanism-linked biomarkers, controlled comparison or combination experiments with standard anticancer therapies, and experimental validation of priority hubs such as AKT1, TNF, SRC, EGFR, BCL2, HIF1A, and MMP9 in immunocompetent syngeneic and clinically relevant patient-derived models.

## 5. Conclusions

This study integrates a scoping review of preclinical evidence with network pharmacology to clarify the antitumor, immune-related, and TME-related effects of SBD-derived interventions. Across diverse cancer models, crude extracts, enriched fractions, derived formulations, and chemically defined constituents showed growth-inhibitory, cell death-related, immunomodulatory, anti-invasive, and microenvironment-remodeling activities. Network pharmacology identified AKT1, TNF, SRC, EGFR, and BCL2 as candidate hub targets associated with tumor survival, inflammatory signaling, immune regulation, and extracellular matrix remodeling. Together, the experimental and computational findings identify recurrent biological processes across SBD-derived interventions, although differences in preparation, chemical characterization, dose, and experimental design limit direct comparison among studies. This integrated framework provides a basis for prioritizing chemically characterized preparations, candidate compounds, and molecular pathways for further mechanistic, pharmacokinetic, and translational investigation.

## Data Availability

No new data were created or analyzed in this study. Data sharing is not applicable to this article.

## Supplementary Materials

The following supporting information can be downloaded at: https://www.mdpi.com/article/10.3390/ijms27188419/s1.

## References

[1] Bray F., Laversanne M., Sung H., Ferlay J., Siegel R.L., Soerjomataram I., Jemal A. (2024). Global cancer statistics 2022: GLOBOCAN estimates of incidence and mortality worldwide for 36 cancers in 185 countries. CA Cancer J. Clin..

[2] Tufail M., Jiang C.-H., Li N. (2025). Immune evasion in cancer: Mechanisms and cutting-edge therapeutic approaches. Signal Transduct. Target. Ther..

[3] Baghban R., Roshangar L., Jahanban-Esfahlan R., Seidi K., Ebrahimi-Kalan A., Jaymand M., Kolahian S., Javaheri T., Zare P. (2020). Tumor microenvironment complexity and therapeutic implications at a glance. Cell Commun. Signal..

[4] Yu H., Li J., Peng S., Liu Q., Chen D., He Z., Xiang J., Wang B. (2025). Tumor microenvironment: Nurturing cancer cells for immunoevasion and druggable vulnerabilities for cancer immunotherapy. Cancer Lett..

[5] Wei G., Li B., Huang M., Lv M., Liang Z., Zhu C., Ge L., Chen J. (2025). Polarization of Tumor Cells and Tumor-Associated Macrophages: Molecular Mechanisms and Therapeutic Targets. MedComm.

[6] Shafqat A., Omer M.H., Ahmed E.N., Mushtaq A., Ijaz E., Ahmed Z., Alkattan K., Yaqinuddin A. (2023). Reprogramming the immunosuppressive tumor microenvironment: Exploiting angiogenesis and thrombosis to enhance immunotherapy. Front. Immunol..

[7] Newman D.J., Cragg G.M. (2020). Natural products as sources of new drugs over the nearly four decades from 01/1981 to 09/2019. J. Nat. Prod..

[8] Atanasov A.G., Zotchev S.B., Dirsch V.M., Supuran C.T. (2021). Natural products in drug discovery: Advances and opportunities. Nat. Rev. Drug Discov..

[9] Sun J., Cao Y., Liu Q., Zhou Z., Xu Y., Liu C. (2024). Chemical Constituents, Anti-Tumor Mechanisms, and Clinical Application: A Comprehensive Review on *Scutellaria barbata*. Molecules.

[10] Gao J., Yin W., Corcoran O. (2019). From *Scutellaria barbata* to BZL101 in Cancer Patients: Phytochemistry, Pharmacology, and Clinical Evidence. Nat. Prod. Commun..

[11] Kan X., Zhang W., You R., Niu Y., Guo J., Xue J. (2017). *Scutellaria barbata* D. Don extract inhibits the tumor growth through down-regulating of Treg cells and manipulating Th1/Th17 immune response in hepatoma H22-bearing mice. BMC Complement. Altern. Med..

[12] Lin J., Feng J., Yang H., Yan Z., Li Q., Wei L., Lai Z., Jin Y., Peng J. (2017). *Scutellaria barbata* D. Don inhibits 5-fluorouracil resistance in colorectal cancer by regulating PI3K/AKT pathway. Oncol. Rep..

[13] Sheng D., Zhao B., Zhu W., Wang T., Peng Y. (2022). *Scutellaria barbata* D. Don (SBD) extracts suppressed tumor growth, metastasis and angiogenesis in Prostate cancer via PI3K/Akt pathway. BMC Complement. Med. Ther..

[14] Shim J.H., Gim H., Lee S., Kim B.J. (2016). Inductions of Caspase-, MAPK- and ROS-dependent Apoptosis and Chemotherapeutic Effects Caused by an Ethanol Extract of *Scutellaria barbata* D. Don in Human Gastric Adenocarcinom Cells. J. Pharmacopunct..

[15] Jin Y., Chen W., Yang H., Yan Z., Lai Z., Feng J., Peng J., Lin J. (2017). *Scutellaria barbata* D. Don inhibits migration and invasion of colorectal cancer cells via suppression of PI3K/AKT and TGF-β/Smad signaling pathways. Exp. Ther. Med..

[16] Yang A.-Y., Liu H.-L., Yang Y.-F. (2022). Study on the mechanism of action of *Scutellaria barbata* on hepatocellular carcinoma based on network pharmacology and bioinformatics. Front. Pharmacol..

[17] Ouyang G., Zhu Y., Ouyang Z. (2025). Investigation of *Scutellaria barbata*’s immunological mechanism against thyroid cancer using network pharmacology and experimental validation. Sci. Rep..

[18] Yang Y., Cheng X., He Y., Chen T., Chen D., Wang A. (2025). Mechanisms of diterpenes from *Scutellaria barbata* D. Don against colorectal cancer via network pharmacology and experimental verification. Sci. Rep..

[19] Arksey H., O’Malley L. (2005). Scoping studies: Towards a methodological framework. Int. J. Soc. Res. Methodol..

[20] Tricco A.C., Lillie E., Zarin W., O’Brien K.K., Colquhoun H., Levac D., Moher D., Peters M.D., Horsley T., Weeks L. (2018). PRISMA extension for scoping reviews (PRISMA-ScR): Checklist and explanation. Ann. Intern. Med..

[21] Peters M.D.J., Marnie C., Tricco A.C., Pollock D., Munn Z., Alexander L., McInerney P., Godfrey C.M., Khalil H. (2020). Updated methodological guidance for the conduct of scoping reviews. JBI Evid. Synth..

[22] Hopkins A.L. (2008). Network pharmacology: The next paradigm in drug discovery. Nat. Chem. Biol..

[23] Li S., Zhang B. (2013). Traditional Chinese medicine network pharmacology: Theory, methodology and application. Chin. J. Nat. Med..

[24] Ru J., Li P., Wang J., Zhou W., Li B., Huang C., Li P., Guo Z., Tao W., Yang Y. (2014). TCMSP: A database of systems pharmacology for drug discovery from herbal medicines. J. Cheminformatics.

[25] Gfeller D., Michielin O., Zoete V. (2013). Shaping the interaction landscape of bioactive molecules. Bioinformatics.

[26] Bhattacharya S., Andorf S., Gomes L., Dunn P., Schaefer H., Pontius J., Berger P., Desborough V., Smith T., Campbell J. (2014). ImmPort: Disseminating data to the public for the future of immunology. Immunol. Res..

[27] Stelzer G., Rosen N., Plaschkes I., Zimmerman S., Twik M., Fishilevich S., Stein T.I., Nudel R., Lieder I., Mazor Y. (2016). The GeneCards suite: From gene data mining to disease genome sequence analyses. Curr. Protoc. Bioinform..

[28] Szklarczyk D., Gable A.L., Lyon D., Junge A., Wyder S., Huerta-Cepas J., Simonovic M., Doncheva N.T., Morris J.H., Bork P. (2019). STRING v11: Protein-protein association networks with increased coverage, supporting functional discovery in genome-wide experimental datasets. Nucleic Acids Res..

[29] Shannon P., Markiel A., Ozier O., Baliga N.S., Wang J.T., Ramage D., Amin N., Schwikowski B., Ideker T. (2003). Cytoscape: A software environment for integrated models of biomolecular interaction networks. Genome Res..

[30] Bader G.D., Hogue C.W.V. (2003). An automated method for finding molecular complexes in large protein interaction networks. BMC Bioinform..

[31] Kanehisa M., Furumichi M., Tanabe M., Sato Y., Morishima K. (2017). KEGG: New perspectives on genomes, pathways, diseases and drugs. Nucleic Acids Res..

[32] Ashburner M., Ball C.A., Blake J.A., Botstein D., Butler H., Cherry J.M., Davis A.P., Dolinski K., Dwight S.S., Eppig J.T. (2000). Gene ontology: Tool for the unification of biology. Nat. Genet..

[33] Yue G.G., Chan Y.-Y., Liu W., Gao S., Wong C.-W., Lee J.K., Lau K.-M., Lau C.B. (2021). Effectiveness of *Scutellaria barbata* water extract on inhibiting colon tumor growth and metastasis in tumor-bearing mice. Phytother. Res..

[34] Zhang P., Zhang T., Wei Y., Yan J., Liu Y., Bao Z., Zeng X. (2025). The antitumor effect of *Scutellaria barbata* D. Don (skullcap) aqueous extract and its mechanism of action in lung adenocarcinoma. Bol. Latinoam. Caribe Plantas Med. Aromat..

[35] Li Y., Zhang J., Zhang K., Chen Y., Wang W., Chen H., Zou Z., Li Y., Dai M. (2022). *Scutellaria barbata* Inhibits Hepatocellular Carcinoma Tumorigenicity by Inducing Ferroptosis of Hepatocellular Carcinoma Cells. Front. Oncol..

[36] Chen W.-W., Gong K.-K., Yang L.-J., Dai J.-J., Zhang Q., Wang F., Li X.-L., Xi S.-C., Du J. (2021). *Scutellaria barbata* D. Don extraction selectively targets stemness-prone NSCLC cells by attenuating SOX2/SMO/GLI1 network loop. J. Ethnopharmacol..

[37] Hu Z., Jing D. (2019). Anti-proliferation effect of *Scutellaria barbata* D. Don extract on lung cancer. Trop. J. Pharm. Res..

[38] Zhang L., Ren B., Zhang J., Liu L., Liu J., Jiang G., Li M., Ding Y., Li W. (2017). Anti-tumor effect of *Scutellaria barbata* D. Don extracts on ovarian cancer and its phytochemicals characterisation. J. Ethnopharmacol..

[39] Wei L.-H., Lin J.-M., Chu J.-F., Chen H.-W., Li Q.-Y., Peng J. (2017). *Scutellaria barbata* D. Don inhibits colorectal cancer growth via suppression of Wnt/β-catenin signaling pathway. Chin. J. Integr. Med..

[40] Chen C.-C., Kao C.-P., Chiu M.-M., Wang S.-H. (2017). The anti-cancer effects and mechanisms of *Scutellaria barbata* D. Don on CL1-5 lung cancer cells. Oncotarget.

[41] Niu J., Hu J., Wang Z. (2024). *Scutellaria barbata* D.Don extract regulates Ezrin-mediated triple negative breast cancer progress via suppressing the RhoA /ROCK1 signaling. Toxicol. Res..

[42] Xue S., Geng A., Lian T., Liu Y. (2022). *Scutellaria barbata* D. Don inhibits cervical cancer cell proliferation, migration, and invasion via miR-195-5p/LOXL2 axis. Toxicol. Res..

[43] Zhang L., Fang Y., Feng J.-Y., Cai Q.-Y., Wei L.-H., Lin S., Peng J. (2017). Chloroform fraction of *Scutellaria barbata* D. Don inhibits the growth of colorectal cancer cells by activating miR-34a. Oncol. Rep..

[44] Li H., Su J., Jiang J., Li Y., Gan Z., Ding Y., Li Y., Liu J., Wang S., Ke Y. (2019). Characterization of polysaccharide from *Scutellaria barbata* and its antagonistic effect on the migration and invasion of HT-29 colorectal cancer cells induced by TGF-β1. Int. J. Biol. Macromol..

[45] Li L., Xu X., Wu L., Zhu H., He Z., Zhang B., Chi Y., Song G. (2019). *Scutellaria barbata* polysaccharides inhibit tumor growth and affect the serum proteomic profiling of hepatoma H22-bearing mice. Mol. Med. Rep..

[46] Sun P., Sun D., Wang X. (2017). Effects of *Scutellaria barbata* polysaccharide on the proliferation, apoptosis and EMT of human colon cancer HT29 Cells. Carbohydr. Polym..

[47] Shan R., Xu X., Yin Y., Liang J., Yuan H., Gao X., Zhao Q., Song G. (2025). Selenium nanoparticles conjugated with *Scutellaris barbata* D. Don polysaccharides: Synthesis, optimization, structural characterization and evaluation of anti-hepatoma activity in vitro and in vivo. Int. J. Biol. Macromol..

[48] Su W., Wu L., Liang Q., Lin X., Xu X., Yu S., Lin Y., Zhou J., Fu Y., Gao X. (2022). Extraction Optimization, Structural Characterization, and Anti-Hepatoma Activity of Acidic Polysaccharides from *Scutellaria barbata* D. Don. Front. Pharmacol..

[49] Bao X., Li L., Xue X. (2019). Flavonoids from *Scutellaria barbata* inhibit activation of tumor-associated macrophages by blocking the Toll-like receptor 4/myeloid differentiation factor 88/nuclear factor-κB signaling pathway. J. Tradit. Chin. Med..

[50] Gao B., Sui X., Choe H., Yang Y., Liu Y., Xia L., Li P., Chen Y., Huo Y., Zhao Z. (2025). Alkaloids isolated from *Scutellaria barbata* D. Don trigger apoptosis and inhibit migration by modulating the p38-p53 pathway in ovarian cancer. J. Ovarian Res..

[51] Liu L., Liu T., Tao W., Liao N., Yan Q., Li L., Tan J., Shen W., Cheng H., Sun D. (2022). Flavonoids from *Scutellaria barbata* D. Don exert antitumor activity in colorectal cancer through inhibited autophagy and promoted apoptosis via ATF4/sestrin2 pathway. Phytomedicine.

[52] Zheng X., Kang W., Liu H., Guo S. (2018). Inhibition effects of total flavonoids from Sculellaria barbata D. Don on human breast carcinoma bone metastasis via downregulating PTHrP pathway. Int. J. Mol. Med..

[53] Chen Y.-Y., Zhu Q., Zhu X.-L., Li H.-H., Li F.-Q. (2024). Study on Chemical Constituents and Cytotoxicity of *Scutellaria barbata* D. Don Oil Against SMMC-7721. Acta Pol. Pharm.-Drug Res..

[54] Wang L., Xu J., Yan Y., Liu H., Karunakaran T., Li F. (2019). Green synthesis of gold nanoparticles from *Scutellaria barbata* and its anticancer activity in pancreatic cancer cell (PANC-1). Artif. Cells Nanomed. Biotechnol..

[55] Ding L., Luo C., Weygant N., Chen W., Ru D., Lai Y., Wang Y., Li H. (2025). Scutellarin suppresses ovarian cancer progression by targeting METTL5. Sci. Rep..

[56] Cao H., Song H., Zhou W., Lv X., Liu X., Xiang Z., Fu R., Cheng Y., Chen J., Wang S. (2025). Exploring the active ingredients of Banzhilian and its mechanism of action on diabetic Gastric cancer based on network pharmacology. Sci. Rep..

[57] Liu F., Zu X., Xie X., Zhang Y., Liu K., Chen H., Wang T., Bode A.M., Dong Z., Kim D.J. (2019). Scutellarin Suppresses Patient-Derived Xenograft Tumor Growth by Directly Targeting AKT in Esophageal Squamous Cell Carcinoma. Cancer Prev. Res..

[58] Huang Z., Yang Y., Fan X., Ma W. (2022). Network pharmacology-based investigation and experimental validation of the mechanism of scutellarin in the treatment of acute myeloid leukemia. Front. Pharmacol..

[59] Zeng S., Chen L., Sun Q., Zhao H., Yang H., Ren S., Liu M., Meng X., Xu H. (2021). Scutellarin ameliorates colitis-associated colorectal cancer by suppressing Wnt/β-catenin signaling cascade. Eur. J. Pharmacol..

[60] Ma H., Yue G.G., Lee J.K.-M., Gao S., Yuen K.-K., Cheng W., Li X., Lau C.B. (2024). Scutellarin, a flavonoid compound from *Scutellaria barbata*, suppresses growth of breast cancer stem cells in vitro and in tumor-bearing mice. Phytomedicine.

[61] Shi H., Liu J., Fan J., He L., Wang X., Tang F., Tian D., He Y. (2022). Molecular Assessment of *Scutellaria barbata* D. Don in the Treatment of Nasopharyngeal Carcinoma Based on Network Pharmacology and Experimental Verification. Evid.-Based Complement. Altern. Med..

[62] Zhang J., Qi C., Li H., Ding C., Wang L., Wu H., Dai W., Wang C. (2023). Exploration of the effect and mechanism of *Scutellaria barbata* D. Don in the treatment of ovarian cancer based on network pharmacology and in vitro experimental verification. Medicine.

[63] Zhang X., Wang J., Fu J., Hu J., Zhang H., Ye M., Yang X., Yu H., Xu H., Lu J. (2024). Dissecting the antitumor effects of *Scutellaria barbata*: Initial insights into the metabolism of scutellarin and luteolin by gut microbiota. J. Pharm. Biomed. Anal..

[64] Hanh T.T.H., Anh D.H., Quang T.H., Trung N.Q., Thao D.T., Cuong N.T., An N.T., Cuong N.X., Nam N.H., Kiem P.V. (2019). Scutebarbatolides A-C, new neo-clerodane diterpenoids from *Scutellaria barbata* D. Don with cytotoxic activity. Phytochem. Lett..

[65] Wang M., Chen Y., Hu P., Ji J., Li X., Chen J. (2020). Neoclerodane diterpenoids from *Scutellaria barbata* with cytotoxic activities. Nat. Prod. Res..

[66] Wang M., Ma C., Chen Y., Li X., Chen J. (2019). Cytotoxic Neo-Clerodane Diterpenoids from *Scutellaria barbata* D.Don. Chem. Biodivers..

[67] Yang G.-C., Hu J.-H., Li B.-L., Liu H., Wang J.-Y., Sun L.-X. (2018). Six New neo-Clerodane Diterpenoids from Aerial Parts of *Scutellaria barbata* and Their Cytotoxic Activities. Planta Med..

[68] Yang G.-C., Liang C., Li S.-G., Liu M., Jia M.-J., Xu X.-N., Wang X.-B., Hua H.-M., Sun L.-X. (2017). Neoclerodane diterpenoids from aerial parts of *Scutellaria barbata*. Phytochem. Lett..

[69] Wang W., Tao Z., Tu L., Huang W., Ye Y., Gao L., Li X. (2024). Diterpenoids from *Scutellaria barbata* D. Don Exert the Antiproliferative Effect on Colorectal Cancer Cells via Hedgehog Signaling Pathway. Pharmacogn. Mag..

[70] Niu C., Li R.-T., Hao X.-S., Qi X., Wang F.-Z., Fei H.-R. (2025). Scutebarbatine B Exerts Anti-Breast Cancer Activity by Inducing Cell Cycle Arrest and Apoptosis Through Multiple Pathways. Phytother. Res..

[71] Feng P.-P., Qi Y.-K., Li N., Fei H.-R. (2021). Scutebarbatine A induces cytotoxicity in hepatocellular carcinoma via activation of the MAPK and ER stress signaling pathways. J. Biochem. Mol. Toxicol..

[72] Yang N., Zhao Y., Wang Z., Liu Y., Zhang Y. (2017). Scutellarin suppresses growth and causes apoptosis of human colorectal cancer cells by regulating the p53 pathway. Mol. Med. Rep..

[73] Guo F., Yang F., Zhu Y.-H. (2019). Scutellarein from *Scutellaria barbata* induces apoptosis of human colon cancer HCT116 cells through the ROS-mediated mitochondria-dependent pathway. Nat. Prod. Res..

[74] Liu J., Jiang M., Li Z., Zhang X., Li X., Hao Y., Su X., Zhu J., Zheng C., Xiao W. (2018). A Novel Systems Pharmacology Method to Investigate Molecular Mechanisms of *Scutellaria barbata* D. Don for Non-small Cell Lung Cancer. Front. Pharmacol..

[75] Shi L., Wu Y., Lv D.L., Feng L. (2019). Scutellarein selectively targets multiple myeloma cells by increasing mitochondrial superoxide production and activating intrinsic apoptosis pathway. Biomed. Pharmacother..

[76] Tan L., Lei N., He M., Zhang M., Sun Q., Zeng S., Chen L., Zhou L., Meng X., Xu H. (2020). Scutellarin protects against human colorectal cancer in vitro by down regulation of hedgehog signaling pathway activity. Int. J. Pharmacol..

[77] Wang Q., Acharya N., Liu Z., Zhou X., Cromie M., Zhu J., Gao W. (2018). Enhanced anticancer effects of *Scutellaria barbata* D. Don in combination with traditional Chinese medicine components on non-small cell lung cancer cells. J. Ethnopharmacol..

[78] Wang G., Jia C., Wang D., Liang L., Li L., Tian G., Feng W. (2025). *Scutellaria barbata* inhibits epithelial-mesenchymal transformation through PI3K/AKT and MDM2 thus inhibiting the proliferation, migration and promoting apoptosis of Cervical Cancer cells. PLoS ONE.

[79] Xue G.-M., Xia Y.Z., Wang Z.-M., Li L.-N., Luo J.-G., Kong L.-Y. (2016). neo-Clerodane diterpenoids from *Scutellaria barbata* mediated inhibition of P-glycoprotein in MCF-7/ADR cells. Eur. J. Med. Chem..

[80] Yang P.-W., Ma S.-P., Chen X.-J., Yang F.-M., Wang S.-M., Wang Q., Zhang S.-H. (2025). Luteolin suppresses cell migration and invasion via targeting miR-6809-5p/FLOT1/FAK and eliciting EMT in hepatocellular carcinoma. Transl. Oncol..

[81] Hanahan D. (2022). Hallmarks of cancer: New dimensions. Cancer Discov..

[82] Wang L., Chen W., Li M., Zhang F., Chen K., Chen W. (2020). A review of the ethnopharmacology, phytochemistry, pharmacology, and quality control of *Scutellaria barbata* D. Don. J. Ethnopharmacol..

