# Supplementary material for: Modulation of Antitumor Immunity and the Tumor Microenvironment by Scutellaria barbata D. Don: A Scoping Review and Network Pharmacology Analysis
Source: Int J Mol Sci. 2026 Sep 21;27(18):8419. doi: 10.3390/ijms27188419 (PMC13607596; doi:10.3390/ijms27188419)
Supplement: Supplementary file 1 [file ijms-27-08419-s001.zip › ijms-4482031-supplementary.pdf]

SUPPLEMENTARY

Supplementary Table S1. Search Strategies Applied Across Databases

Database	Search Strategy	Records Retrieved
**PubMed**	((cancer*) OR (carcinoma*) OR (neoplasm*) OR (tumor*) OR (malignanc*)) AND (("Scutellaria barbata") OR ("Scutellaria barbata D. Don") OR ("Ban Zhi Lian") OR ("Ban-Zhi-Lian") OR ("Barbat skullcap"))	**214**
**EMBASE**	('cancer'/exp OR cancer*:ab,ti OR carcinoma*:ab,ti OR neoplasm*:ab,ti OR tumor*:ab,ti OR malignanc*:ab,ti) AND ('scutellaria barbata'/exp OR scutellaria barbata:ab,ti OR 'ban zhilian':ab,ti OR 'ban-zhi-lian':ab,ti OR 'barbat skullcap':ab,ti)	**267**
**CENTRAL**	(cancer* OR carcinoma* OR tumor* OR malignanc*) AND ("Scutellaria barbata" OR "ban zhi lian" OR "ban-zhi-lian" OR "barbat skullcap")	**6**
**OASIS**	“반지련”	**11**

Supplementary Table S2. Preclinical evidence of the antitumor activity and growth inhibition of SBD

Study ID	Intervention	Dose / Exposure conditions	Comparator	Main findings of the study
**Bao 2019[49]**	SBD flavonoids (SBF)	0–800 μg/mL for 24–48 h (cell viability assay, MTT)	U937 untreated control; TAM control (co-culture without treatment)	1. Cell proliferation ↓ in Ishikawa endometrial cancer cells;
**Cao 2025[56]**	Quercetin	In vitro: 1.56–200 μM for 24–72h; In vivo: 50 mg/kg i.p. every other day × 20 doses	HGC-27 normal glucose cells; HGC-27-HG (high-glucose) control; vehicle-treated mice	1. Cell proliferation ↓ (cell viability↓, colony formation↓, EdU+ proliferation↓, Ki-67↓); 2. Cell cycle arrest ↑ (G0/G1 phase); 3. Apoptosis ↑ (Annexin V+, Bax↑, cleaved caspase-3↑, Bcl-2↓, TUNEL+); 4. Cell migration ↓ and invasion ↓; 7. Tumor growth ↓ in vivo (tumor volume↓, tumor weight↓).
**Chen 2017[40]**	SBD methanol extract (MESB)	In vitro: 0–5 mg/mL for up to 24 h; In vivo: 60 mg/kg intraperitoneal injection, 6 times/week for 15 days	Vehicle control; normal lung cells (MRC5)	1. Cell proliferation ↓ (cell viability↓, PCNA↓) with no cytotoxicity in normal cells; 2. Cell cycle arrest ↑ (G2/M phase); 3. Apoptosis ↑ (Annexin V+, TUNEL+, caspase-3/7 cleavage↑, PARP cleavage↑); 4. Autophagy ↑; 5. Tumor progression ↓ (angiogenesis↓); 6. Tumor growth ↓ in vivo; 12. Combination effect ↑ (enhanced anti-cancer effects with etoposide or cisplatin).
**Chen 2021[36]**	SBD methanol extract (MESB)	In vitro: 30–90 μg/mL for up to 48 h; In vivo: according to previous study	Vehicle control; normal epithelial cells	1. Cell proliferation ↓ (cell viability↓, colony formation↓); 2. Cancer stemness ↓ (sphere formation↓, stemness-related gene expression↓); 3. Tumor growth ↓ in vivo.
**Chen 2024[53]**	SBD oil (SBO)	25–100 μg/mL for 48 h	Vehicle control	1. Cell proliferation ↓ (cell viability↓); 2. Apoptosis ↑ (nuclear condensation, Annexin V+, DNA fragmentation);
**Ding 2025[55]**	Scutellarin (SCU)	0–240 μM for 24 h (cell viability, CCK-8);10–50 μM for 10–14 days (colony formation);10–50 μM for 24–48 h (migration assays); 10–50 μM	Untreated control (vehicle)	1. Cell proliferation↓ in ovarian cancer cell lines (SKOV3, A2780, OVCAR8; no significant effect in OVCAR3); 2. Colony formation ability↓ across all cell lines, even below IC50 concentrations; 3. Cell migration↓ (wound healing, transwell) in a dose-dependent manner;

		for organoid treatment (duration NR);		4. Organoid growth ↓ and viability ↓ (size ↓, number ↓, ATP ↓, structural collapse, PI+ dead cells ↑);
**Feng 2021[71]**	Scutebarbatine A (SBT-A)	0.01–1.6 μM for 48 h (CCK-8); 0.05–0.4 μM for 24 h (colony assay); 0–0.2 μM for 24 h (cell cycle, apoptosis, WB)	Vehicle control; SBT-A alone	1. Cell viability ↓ dose-dependently (IC50: HepG2 0.53 μM, Huh7 0.31 μM); 2. Colony formation ↓; 3. G2/M arrest ↑ and G0/G1 ↑; 4 cyclin B1/D1 ↓, p-Cdc2 ↓, p-Cdc25C ↓; 5. p21 ↑, p27 ↑ (HepG2); 6. Apoptosis ↑ (TUNEL); 7. Cleaved caspase-8/9 ↑, PARP ↑.
**Gao 2025[50]**	SBD alkaloid fraction (SBA)	In vitro: 10–20 mg/mL for 24 h; In vivo: 25–50 mg/kg/day (oral) for 21 days; cisplatin 2 μg/mL (in vitro, combination treatment)	Vehicle; DDP; SBA; combination	1. Cell viability ↓ dose- & time-dependently (IC50: 30.39 → 16.43 mg/mL); 2. Colony formation ↓; 3. Apoptosis ↑ (Cleaved caspase-3/9 ↑, Bax ↑ and Bcl-2 ↓); 4. Migration ↓ (wound healing, transwell); 8. Tumor volume ↓ and tumor weight ↓ with Ki67 ↓; 9. SBA ↑ cisplatin-induced apoptosis and growth inhibition
**Guo 2019 [73]**	Scutellarein	NR	Control	1. Cell viability ↓; 2. Apoptosis ↑ (Hoechst staining, DNA fragmentation); 3. ROS ↑ with Mitochondrial membrane potential ↓; 4. Activation of mitochondrial apoptotic pathway (Caspase-3 activity ↑, Bcl-2 ↓ and Bax ↑, Cytochrome c release ↑); 5. Apoptosis via ROS-mediated mitochondria-dependent pathway
**Hanh 2019[64]**	Scutebarbatolides A–C and related diterpenoids (compounds 1–9)	NR (IC50-based cytotoxicity assay)	Ellipticine (positive control)	1. Moderate cytotoxicity observed for compounds 1 and 6 (IC50: 30.8–51.1 μM); 2. Weak to moderate cytotoxicity for compound 2 (IC50: 51.9–76.2 μM); 3. Weak cytotoxicity for compound 4 (IC50: ~66–73 μM); 4. Compounds 3, 5, and 7–9 were inactive (IC50 > 100 μM)
**Hu 2019[37]**	SBD ethanol extract (SBDE)	30–240 μg/mL for 24–72 h (MTT); 62.5–250 μg/mL for 24–72 h (morphology analysis); 40 μg/mL for 24–48 h (cell cycle analysis); 30–240 μg/mL for 24–48 h (flow cytometry, immunocytochemistry)	Cisplatin (positive control); vehicle (0.04% DMSO)	1. Cell proliferation ↓ and Cell viability ↓; 2. G0/G1 phase arrest ↑ with S and G2/M ↓; 3. Apoptosis ↑ (Caspase-3 ↑, caspase-8 ↑, caspase-9 ↑, Bcl-2 ↓); 4. Ki-67 ↓, p21 ras ↓; 5. Apoptosis via mitochondria-related caspase pathway

**Huang 2022[58]**	Scutellarin (SCU)	In vitro: various concentrations for 72 h (IC50 = 35.99 μM for HL-60, 45.26 μM for THP-1, 62.61 μM for KG-1); In vivo: breviscapine containing ≥90% scutellarin, 60 mg/kg/day orally for 21 days	Vehicle control (normal saline)	1. Cell viability ↓ dose-dependently (IC50: ~35.99–62.61 μM); 2. Apoptosis ↑ dose-dependently; 6. Xenograft tumor growth ↓ in vivo.
**Jin 2017[15]**	SBD ethanol extract (EESB)	0–2 mg/mL for 24–48 h (MTT); 0.125–0.5 mg/mL for 24 h (migration/invasion, WB)	Untreated control	1. Non-cytotoxic conditions confirmed at ≤0.5 mg/mL, while cytotoxicity was observed at ≥1 mg/mL; 2. Migration ↓ and Invasion ↓ dose-dependently (MMP1, MMP2, MMP3/10, MMP9, MMP13 ↓); 3. E-cadherin and N-cadherin ≈ no significant change → EMT-independent mechanism;
**Kan 2017[11]**	SBD extract (SBE)	In vitro: 0.05–0.5 mg/mL for 48h; In vivo: 50–150 mg/kg/day × 30 days; IL-17A injection model	Vehicle; SBE; SBE + IL-17A	1. HepG2 cell proliferation ↓ dose-dependently; 2. Tumor growth ↓ in H22-bearing mice;
**Li 2019a[45]**	SBD polysaccharides (SBPS)	In vivo: 50–200 mg/kg/day × 7 days (H22 hepatoma mice); CTX control included	Model; CTX; SBPS groups	1. Tumor weight ↓ significantly (all SBPS doses); 2. Moderate-dose SBPS showed highest tumor inhibition (~49.68%)
**Li 2019b[44]**	SBD polysaccharide SBPW3	In vitro: 25–800 μg/mL (CCK-8, 36 h); migration/invasion assays (±TGF-β1 10 ng/mL); WB (EMT markers); In vivo: 100–200 mg/kg (metastasis model, 31 days)	Control; TGF-β1-induced model; SBPW3-treated	1. Cell viability ≈ unchanged (non-cytotoxic); 3. migration ↓ (wound healing); 4. invasion ↓ (transwell); 7. Liver metastasis ↓ in vivo; 8. Metastatic nodules ↓ dose-dependently;
**Li 2022[35]**	SBD crude preparation	In vitro: 0–50 mg/mL for 48 h; IC50 concentration used for mechanistic assays; In vivo: Intragastric administration (140 g/10 g body weight)	Vehicle control (PBS-treated cells or mice)	1. Cell viability ↓ dose-dependently (CCK-8); 2. Cytotoxicity ↑ (LDH release ↑); 3. Cell proliferation ↓ (EdU); 5. Tumor volume ↓ and weight ↓ in vivo; 6. Ki-67 ↓ in tumor tissue.

**Lin 2017[12]**	SBD ethanol extract (EESB)	HCT-8/5-FU cells, 0.5–1.5 mg/mL, 24–48 h; MTT, colony formation, apoptosis assay, Rh-123 assay, RT-PCR, WB	5-FU-resistant cells vs parental; untreated control	1. 5-FU resistance confirmed (RI ≈25.34); 2. EESB reduced viability of resistant cells dose-dependently; 3. Cell proliferation ↓ (colony formation ↓); 4. Apoptosis ↑ (DAPI, Annexin V/PI);
**Liu 2018[74]**	Baicalein	In vitro: H1975 cells (5–20 μM, 24–48 h), RAW264.7 (LPS-induced); In vivo: LLC tumor-bearing mice (1.5 mg/kg/day)	Control (vehicle)	1. Cell viability ↓ (dose-dependent, H1975); 2. CDK2 ↓; 3. Bax ↑, Bcl-2 ↓; 6. Tumor volume ↓ and tumor weight ↓ in vivo. 7. Survival ↑ in tumor-bearing mice;
**Liu 2019[57]**	Scutellarin (SCU)	In vitro: ESCC cells (KYSE30/450/510, 48 h; MTT, colony, WB, FACS); In vivo: PDX mice (100 mg/kg)	Control (vehicle), normal esophageal cells	2. ESCC cell viability ↓ (dose-dependent); 3. Anchorage-dependent & -independent growth ↓; 4. G2 phase cell cycle arrest ↑; 5. p27 ↑; 7. PDX tumor volume ↓; 8. Ki-67 ↓ (proliferation marker)
**Liu 2022[51]**	SBD flavonoids (FSB)	In vitro: HCT116 (24 h, 75–300 μg/ml; MTT, EdU, apoptosis, migration, WB); In vivo: xenograft (100–200 mg/kg/day)	Control (vehicle), normal IEC-6 cells	1. Cell viability(dose-dependent, IC50 ≈174 μg/ml) and proliferation ↓ (EdU); 2. Apoptosis ↑, evidenced by ↑ cleaved caspase-3 and Bax and ↓ Bcl-2; 4. Migration ↓, accompanied by ↓ MMP-2 and MMP-9; 9. Tumor growth ↓ in vivo, with ↑ tumor necrosis
**Ma 2024[60]**	Scutellarin (SCU) and SBD water extract (SBW)	In vitro: BCSCs (MTT, BrdU, sphere, colony, migration); In vivo: NOD/SCID mice (50–100 mg/kg)	Vehicle, paclitaxel	1. Cell viability and proliferation ↓, accompanied by ↓ colony formation; 2. Cancer stemness ↓, evidenced by ↓ sphere formation, ↓ CD44 expression, and reduced tumorigenicity following SCU pre-treatment; 3. Migration and metastasis ↓, evidenced by ↓ migration and ↓ lung metastasis; 4. Tumor growth ↓ in vivo, evidenced by ↓ tumor volume/weight and ↓ Ki-67 expression
**Niu 2024[41]**	SBD petroleum ether extract	0–500 μg/mL for 24 h (CCK-8); 500 μg/mL for functional assays (colony formation, transwell, apoptosis, WB)	Vehicle control (0 μg/mL)	1. SB ↓ viability dose-dependently; 2. SB ↓ colony formation; 3. SB ↓ migration & ↓ invasion; 4. SB ↑ apoptosis; 5. SB ↑ Bax, ↑ cleaved caspase-3, ↓ Bcl-2.
**Niu 2025[70]**	Scutebarbatine B (SBT-B), a diterpenoid alkaloid	In vitro: 0.0125–0.8 μM for 24 h (viability); 0.05, 0.1 μM for migration; 0.1, 0.2, 0.4 μM for apoptosis/ROS; In vivo: 0.1	Vehicle control (0 μM, 0.1% DMSO); doxorubicin 1 mg/kg (positive	1. SBT-B ↓ viability dose-dependently (IC50: MCF-7 0.575 μM, MDA-MB-231 0.519 μM); 2. SBT-B ↓ EdU+ proliferating cells; 3. SBT-B ↓ migration & ↓ invasion; 4. SBT-B ↑ γ-H2AX (DNA damage); 5.

	isolated from SBD	mg/kg i.p. every 2 days (xenograft)	control in vivo); NAC (ROS scavenger); SP600125 (JNK inhibitor, 20 μM)	SBT-B → G2/M arrest; ↓ cyclinB1, ↓ cyclinD1, ↓ Cdc2, ↑ p27; 6. SBT-B ↑ apoptosis; ↑ cleaved caspase-8/-9, ↑ cleaved PARP, ↑ DR5, ↓ DR4; 7. In vivo: ↓ tumor volume & ↓ tumor weight; no body weight change; no major organ toxicity; ↓ Ki67.
**Ouyang 2025[17]**	Quercetin (SBD component)	ACT-1 thyroid cancer cells, 48 h; MTT, colony assay, apoptosis assay, RT-qPCR	Normal thyroid cells (Nthy-ori 3-1), control	1. TC cell viability ↓ (dose-dependent, IC50 ≈119 μM); 2. Colony formation ↓; 3. Apoptosis ↑ (flow cytometry).
**Shan 2025[47]**	SBD selenium-polysaccharide (SBP-Se)	In vitro: HepG2 (CCK-8, migration, colony); In vivo: mouse xenograft	SBP, SeNPs, control	1. Cell viability and proliferation ↓ in a dose-dependent manner, accompanied by ↓ colony formation; 2. Migration ↓ (transwell) 3. Tumor growth ↓ in vivo, evidenced by ↓ tumor volume and weight
**Sheng 2022[13]**	SBD extract	PCa cells (PC-3, DU145), 24–48 h; in vivo xenograft; migration/invasion/angiogenesis assays	Control, MK22006 (AKT inhibitor)	1. Cell viability and proliferation ↓, accompanied by ↓ colony formation and cell cycle arrest (S/G2/M); 2. Apoptosis ↑, evidenced by ↑ Bax and cleaved caspase-3 and ↓ Bcl-2; 5. Migration and invasion ↓; 8. Tumor growth ↓ in vivo, evidenced by ↓ tumor volume/weight and ↓ Ki67 expression.
**Shi 2019[75]**	Scutellarein	In vitro: MM cells (MM.1R, IM-9), 24 h; ROS, mitochondrial assay, WB; In vivo xenograft	In vitro: MM cells (MM.1R, IM-9), 24 h; ROS, mitochondrial assay, WB; In vivo xenograft	1. Cell viability ↓, accompanied by apoptosis induction (↑ TUNEL positivity and caspase-3/9 activation); 3. Mitochondrial apoptosis activation, evidenced by ↑ cytochrome c release, ↑ Bax/↓ Bcl-2, and ↓ mitochondrial membrane potential; 7. Tumor burden ↓ in vivo, accompanied by increased apoptosis marker expression; 8. Enhanced apoptosis observed in combination with bortezomib
**Shi 2022[61]**	Baicalein, Wogonin	NPC cells (CNE2, 5-8F), 24–48 h; CCK-8, apoptosis assay, WB	Control, cisplatin	1. Cell viability and proliferation ↓, accompanied by increased apoptosis
**Shim 2016[14]**	SBD ethanol extract (ESB)	MKN-45 cells, 24–72 h; MTT, FACS (sub-G1), caspase assay, JC-1, ROS assay, MAPK inhibitors, NAC	Control, MAPK inhibitors, NAC, chemo drugs	1. Cell viability ↓, accompanied by cell cycle arrest and increased sub-G1 fraction; 2. Apoptosis ↑, evidenced by ↑ caspase-3/9 activity and mitochondrial membrane depolarization; 6. Enhanced cell death observed in combination with chemotherapeutic agents

**Su 2022[48]**	SBD acidic polysaccharide SBP-2A	HepG2 cells, 50–3200 μg/mL, 48 h; MTT, colony formation, EdU, flow cytometry, qPCR, WB	Control	1. Cell viability (dose-dependent, IC50 ≈548 μg/mL) and proliferation (EdU) ↓, accompanied by ↓ colony formation; 2. Cell cycle arrest, evidenced by ↑ G1 phase and ↓ S phase; 3. Apoptosis ↑, accompanied by ↑ Bax and ↓ Bcl-2
**Sun 2017[46]**	SBD polysaccharide SPS2p	HT29 cells, 10–80 mg/L, 24–96 h; MTT, apoptosis assay, WB, qPCR, ELISA	Control	1. Cell proliferation ↓ (dose/time-dependent); 2. apoptosis ↑ (Annexin V, Hoechst) with Bax ↑, Bak ↑, and Bcl-2 ↓
**Tan 2020[76]**	Scutellarin (SCU)	HCT116 cells, 5–20 μg/mL, 24–72 h; MTT, wound healing, soft agar, qPCR, WB	Control, GANT61	1. Cell proliferation ↓ (dose-dependent); 2. migration ↓ (wound healing) and anchorage-independent growth ↓ (soft agar); 3. Hedgehog signaling ↓, as indicated by c-Myc ↓ (mRNA/protein), SHh ↓, Ptch1 ↓, and Gli1 ↓.
**Wang 2018[77]**	SBD and Long-Jin-San-Jie formula (LJSJF)	NSCLC cells (A549, H460, H1650, H1975), 48 h; MTT, soft agar, qPCR, WB	Control, SB vs LJSJF	1. Cell viability ↓ in a dose-dependent manner, with LJSJF showing greater cytotoxicity than SB (synergistic effect); 2. colony formation ↓ (tumorigenicity); 3. apoptosis ↑ via activation of multiple apoptotic pathways, accompanied by survivin ↓, MDM2 ↓, p21 ↑ (A549), p73 ↑ (H460), and p53 ↑ (A549) / ↓ (H460); 4. oncogenic signaling ↓, as indicated by EGFR ↓, mTOR ↓, p38MAPK ↓, and Cyclin D1 ↓.
**Wang 2019a[66]**	Neoclerodane diterpenoids (1-16)	Cancer cell lines (LoVo, MCF-7, SMMC-7721, HCT-116), 48 h; MTT assay	Cisplatin	1. Cytotoxicity ↑ across multiple compounds, with compound 14 showing the strongest activity (IC50 ≈ 4–7 μM) and selective cytotoxicity ↑ (higher sensitivity in LoVo cells); 2. structure–activity relationship (SAR) analysis indicated that ester moieties are important and benzoyloxy substitution ↑ activity.
**Wang 2019b[54]**	SBD-derived gold nanoparticles (AuNPs)	PANC-1 cells, 25–50 μg/mL, 24 h; MTT, AO/PI staining, ROS assay, WB, qRT-PCR	Control	1. Cell viability ↓ (dose-dependent); 2. apoptosis ↑ (AO/PI staining, nuclear condensation) via mitochondria-mediated pathways, accompanied by ROS ↑, Bax ↑, Bcl-2 ↓, Bid ↓, caspase-3 ↑, caspase-9 ↑, and apoptotic gene expression ↑ (qRT-PCR).

**Wang 2020[65]**	Neoclerodane diterpenoids (barbatin F, G, scutebata A–P)	Various concentrations for IC50 determination; 48 h exposure (MTT assay)	Vehicle control	1. Cytotoxicity varied across compounds, with compounds 1, 2 showing weak activity (IC50 ≈ 32.8–44.3 μM), compounds 3, 4 showing moderate activity (IC50 ≈ 5.31–28.5 μM), and compounds 5, 6 showing low or inactive effects; 2. notably, compound 3 exhibited the strongest cytotoxic activity.
**Wang 2024[69]**	SBD diterpenoid Bzl-D	5–40 μg/mL for 24 h (CCK-8, apoptosis, migration/invasion assays); in vivo 50 mg/kg/day ×16 days	Control (vehicle/saline)	1. Cell viability ↓ and proliferation ↓ in a dose-dependent manner (IC50 ≈ 22.75 μg/mL in HT-29, highest sensitivity); 2. apoptosis ↑ (flow cytometry) with early apoptotic cells ↑; 3. cell cycle arrest ↑ at the G2/M phase; 4. Migration ↓ and invasion ↓; 6. Tumor growth ↓ in vivo (tumor volume ↓, tumor weight ↓, pathological mitosis ↓).
**Wang 2025[78]**	SBD active components baicalin and wogonin	0–10 mg/mL, 24–72 h (CCK-8, Transwell, TUNEL, RT-PCR, WB)	Untreated control	1. Cell viability ↓ and proliferation ↓; 2. apoptosis ↑ (including combination treatment); 3. migration ↓ with migration inhibition ↑, indicating metastatic potential ↓; 6. overall anti-tumor effect ↑.
**Wei 2017[39]**	SBD ethanol extract (EESB)	In vitro: 0, 0.5, 1.5, 2.5 mg/mL for 24 h (HT-29 cells); In vivo: 2 g/kg, intragastric administration, 5 days/week for 16 days (CRC xenograft mice)	Untreated control (saline / vehicle)	1. Tumor growth ↓ in vivo, as evidenced by tumor weight ↓ and Ki-67 ↓; 2. cell viability ↓ and colony formation ↓ in a dose-dependent manner; 3. Wnt/β-catenin signaling ↓, as indicated by β-catenin ↓, c-Myc ↓, survivin ↓, and APC ↑ with β-catenin phosphorylation ↑.
**Xue 2016[79]**	SBD-derived neoclerodane diterpenoids	Cytotoxicity screening: 50 μM (MCF-7 cells); MDR reversal assay: 20 μM (MCF-7/ADR cells); Additional assays: 10–20 μM (ADR accumulation, Rh123 assay, Western blot, etc.)	Untreated control; Positive control: verapamil (P-gp inhibitor)	1. Cytotoxicity ↓ (generally weak across compounds); 2. multidrug resistance (MDR) reversal ↑, as indicated by decreased IC50 of adriamycin and increased chemosensitivity; 3. intracellular drug accumulation ↑ (ADR ↑, Rh123 ↑), leading to enhanced cancer cell death ↑.
**Xue 2022[42]**	SBD chloroform extract (ECSB)	0, 0.2, 0.5 mg/mL for 24 h (CCK-8, TUNEL, Transwell); IC50: HeLa 0.504 mg/mL, HT-3 0.525 mg/mL	Untreated control (0 mg/mL); normal cervical	1. Cell viability ↓ dose-dependently in HeLa and HT-3 cells; no significant cytotoxicity in HcerEpiC normal cells; 2. Apoptosis ↑

			epithelial cells (HcerEpiC)	(TUNEL) dose-dependently; 3. Migration ↓ (Transwell) dose-dependently; 4. Invasion ↓ (Transwell) dose-dependently.
**Yang 2017a[72]**	Scutellarin (SCU)	10, 30, 100 μM for 24–48 h (WB for p53, p21, Bcl-2, Bax, caspase-3)	DMSO control	1. Cell viability ↓ dose- and time-dependently in HCT-116 colon cancer cells; 2. Apoptosis ↑ (Hoechst 33342 staining, AO/EB staining, TUNEL assay); 3. Bcl-2 ↓ and Bax ↑ dose-dependently; 4. Active caspase-3 ↑; 5. p-p53 ↑ and p21 ↑; 6. p53 inhibitor (pifithrin-α) abrogated Scutellarin-induced apoptosis, confirming p53-dependent mechanism.
**Yang 2017b[68]**	SBD-derived neoclerodane diterpenoids, including scutebatas U–W	0–100 μg/mL for 48 h (MTT assay)	Vehicle control	1. No significant cytotoxicity observed against A549 (lung), MCF7 (breast), or SGC7901 (gastric) cancer cell lines (IC50 > 100 μg/mL for all three compounds).
**Yang 2018[67]**	Neoclerodane diterpenoids (scutebatas X–Z, A1–C1, compounds 1–6)	0–100 μg/mL for 48 h (MTT assay)	Cisplatin (positive control)	1. Compound 6 (scutebata C1) showed cytotoxic activity against all three cell lines (IC50: SGC-7901 17.9 μM, MCF-7 29.9 μM, A-549 35.7 μM); 2. Compound 1 showed selective cytotoxicity against MCF-7; compound 4 showed selective cytotoxicity against A-549; 3. Compounds 2, 3, and 5 were inactive (IC50 > 40 μM) against all three cell lines.
**Yang 2022[16]**	Rhamnazin and luteolin, active components of SBD	Various concentrations for 24 h (CCK-8 assay); IC50: Luteolin 202.1 μM (Hep3B), 210.5 μM (HepG2); Rhamnazin 303.9 μM (Hep3B), 339.6 μM (HepG2)	Untreated control (vehicle DMSO)	1. HCC cell viability ↓ in HepG2 and Hep3B cells dose-dependently for both Rhamnazin and Luteolin (CCK-8).
**Yang 2025a[18]**	BZL-5, a barbatin A diterpene isolated from SBD	0, 2, 4, 8, 16 μM for 72 h (SRB assay); colony formation, Transwell, wound-healing assays at 0, 2, 4, 8 μM; apoptosis assay (48 h)	Untreated control; FHC normal colon epithelial cells	1. HCT-116 cell viability ↓ dose-dependently (IC50 ≈ 4.35 μM); no significant cytotoxicity in FHC normal cells; 2. Colony formation ↓ dose-dependently; 3. Cell migration ↓ (Transwell assay) dose-dependently; 4. Wound healing ↓ (scratch assay) dose-dependently;

				5. Apoptosis ↑ (Annexin V-FITC/PI, flow cytometry) dose-dependently.
**Yang 2025b[80]**	Luteolin (3′,4′,5,7-tetrahydroxyflavone), a flavonoid isolated from SBD	In vitro: 20 μM for 24, 48, and 72 h (CCK-8); 20 μM for 24 h (wound healing, transwell, adhesion, qRT-PCR, WB); transfection with LV-oe-miR-6809-5p and/or LV-oe-FLOT1	Vehicle control (DMSO); LV-miR-NC; LV-anti-miR-6809-5p; LV-oe-FLOT1; luteolin + miR-6809-5p overexpression; luteolin + FLOT1 overexpression	1. Luteolin ↓ viability time-dependently; 2. Luteolin ↓ migration; 3. Luteolin ↓ invasion; 4. Luteolin ↓ cell adhesion.
**Yue 2021[33]**	SBD water extract (SBW) and scutellarin (SCU)	In vitro: SBW 50–800 μg/mL, Scu 9.75–156 μM for 48 h (MTT, Transwell migration/invasion); In vivo: SBW 615 mg/kg (SBW-L), 1,230 mg/kg (SBW-H), Scu 3.5 mg/kg (Scu-L) or 7 mg/kg (Scu-H) orally for 4 weeks (HCT116 xenograft nude mice and Colon26-Luc orthotopic model)	Untreated control; human fibroblasts Hs27 (normal cell cytotoxicity comparison)	1. Cell viability ↓ dose-dependently; no significant cytotoxicity in normal Hs27 cells; 2. Cell migration ↓ dose-dependently; 3. Cell invasion ↓ dose-dependently; 4. Tumor weight ↓ in vivo (xenograft model); 5. Lung metastatic burden ↓ in vivo; 6. Orthotopic tumor burden ↓ in vivo; 7. No hepatotoxicity or muscle toxicity (ALT, AST, LDH, CK normal).
**Zeng 2021[59]**	Scutellarin (SCU)	In vitro: 60–400 μM for 48 h (MTT); 90, 180, 360 μM for 24–48 h (wound healing, Hoechst 33258); In vivo: 25, 50, 100 mg/kg/day i.p. × 3 cycles (AOM/DSS-induced CAC C57BL/6 mice); Positive control: 5-FU 20 μM (in vitro) / 20 mg/kg (in vivo)	Vehicle control (1‰ DMSO); model group (AOM/DSS without treatment)	1. Cell proliferation ↓ concentration-dependently in HT-29 CRC cells (MTT); 2. Cell migration ↓ dose-dependently at 24 h and 48 h (wound healing assay); 3. Apoptosis ↑ in HT-29 cells (Hoechst 33258: nuclear condensation and shrinkage); 4. Bax ↑ and Bcl-2 ↓ at mRNA and protein level in HT-29 cells; 5. Tumor load ↓ and colorectal length restored in AOM/DSS CAC mice dose-dependently; 6. Survival rate ↑ and body weight ↑ in CAC mice; 7. Bax ↑ and Bcl-2 ↓ at mRNA and protein level in CAC tissue of mice.

**Zhang 2017a[38]**	SBD ethanol crude extract	0, 50, 100, 200, 400, 600 μg/mL for 24–48 h (MTT); 0, 100, 200, 400 μg/mL for 24 h (DAPI staining, Annexin V/PI flow cytometry, WB); 0, 50, 100 μg/mL for 24 h (wound healing, Transwell)	Vehicle control (untreated cells); normal ovarian epithelial cells IOSE80 (cytotoxicity comparison)	1. Cell viability ↓ dose- and time-dependently in A2780 and SK-OV-3 ovarian cancer cells (MTT); no significant effect on normal IOSE80 cells, indicating selective cytotoxicity; 2. Apoptosis ↑ (DAPI: nuclear condensation and apoptotic bodies; Annexin V/PI: early and late apoptotic cell fraction ↑ dose-dependently); 3. Cleaved caspase-3 ↑ and cleaved caspase-9 ↑ dose-dependently (mitochondrial apoptosis pathway); 4. Bcl-2 ↓ dose-dependently; 5. Cell migration ↓ dose-dependently (wound healing: closure rate 40% at 50 μg/mL, 20% at 100 μg/mL vs. control); 6. Cell migration ↓ dose-dependently (Transwell: migrated cells reduced by 75% and 87.5% at 50 and 100 μg/mL); 7. MMP-2 ↓ and MMP-9 ↓ dose-dependently (Western blot).
**Zhang 2017b[43]**	SBD chloroform fraction (ECSB)	0, 100, 150, 200 μg/mL for 24–48 h (MTT, colony formation, Annexin V/PI flow cytometry, WB, qRT-PCR)	Untreated control (vehicle DMSO ≤0.25%)	1. Cell viability ↓ dose- and time-dependently in HCT-8 colon cancer cells (24 h: 98.4→36.7%; 48 h: 86.4→23.5%); 2. Colony formation ↓ dose-dependently (survival rate reduced by 27.1%, 49.2%, 63.0% at 100, 150, 200 μg/mL); 3. Apoptosis ↑ dose-dependently (Annexin V/PI: early + late apoptosis 10.4→23.3%); 4. Bcl-2 mRNA ↓ and protein ↓ dose-dependently.
**Zhang 2023[62]**	Quercetin, luteolin, and wogonin, active components of SBD	IC50 concentrations for 48 h (quercetin 121.43 μmol/L, luteolin 95.27 μmol/L, wogonin 115.57 μmol/L); 0–150 μmol/L for 24–48 h (viability)	Vehicle control (DMSO)	1. Quercetin, luteolin, and wogonin ↓ viability of SKOV3 cells dose- and time-dependently; 2. Quercetin, luteolin, and wogonin ↓ colony formation.
**Zhang 2024[63]**	Scutellarin, luteolin, and microbiota-derived metabolites	0–100 μg/mL for 48 h (CCK-8, HepG2)	Vehicle control (DMSO)	1. Scutellarin ↓ viability of HepG2 (IC50 = 16.64 μg/mL); 2. Luteolin ↓ viability of HepG2 (IC50 = 25.05 μg/mL); 3. Scutellarein showed strongest antiproliferative activity (IC50 = 10.29 μg/mL); 4. Hydroxygenkwanin ↓ viability (IC50 = 15.70 μg/mL); 5. Scutellarein tetramethyl ether ↓ viability (IC50 = 20.08 μg/mL); 6. Naringenin,

				desaminotyrosine, and p-hydroxycinnamic acid showed weaker activity (IC50 = 99.98, 521.90, 434.60 μg/mL, respectively).
**Zhang 2025[34]**	SBD aqueous extract (SBE), with wogonin as an active ingredient	In vitro: 0, 40, 80, 120, 240, 360 μg/mL for 24–72 h (CCK-8); 0, 120, 240, 360 μg/mL for 24 h (wound healing); Transwell invasion assay; WB for MMP-2, MMP-9, CDK1 at 0, 240, 360 μg/mL	Untreated control	1. Cell viability ↓ dose- and time-dependently in A549 lung adenocarcinoma cells (CCK-8); 2. Cell migration ↓ dose-dependently (wound healing assay); 3. Cell invasion ↓ dose-dependently (Transwell assay); 4. MMP-2 ↓ and MMP-9 ↓ dose-dependently (Western blot).
**Zheng 2018[52]**	Total flavonoids of SB (TF-SB)	In vitro: 40–200 μg/mL for 24–72 h; In vivo: 50, 100, 200 mg/kg by gavage for 4 weeks	Vehicle control (distilled water)	1. TF-SB ↓ viability of MDA-MB-231 dose- and time-dependently; 2. TF-SB ↓ migration dose-dependently; 3. TF-SB ↓ invasion dose-dependently; 4. In vivo: TF-SB ↓ osteoclast cell number dose-dependently; no significant effect on tumor weight (except at 200 mg/kg); no effect on mouse survival.

AML, acute myeloid leukemia; AOM/DSS, azoxymethane/dextran sulfate sodium; AuNPs, gold nanoparticles; BCSC, breast cancer stem cell; CAC, colitis-associated colorectal cancer; CRC, colorectal cancer; EMT, epithelial–mesenchymal transition; ER stress, endoplasmic reticulum stress; ESCC, esophageal squamous cell carcinoma; HCC, hepatocellular carcinoma; MAPK, mitogen-activated protein kinase; MDR, multidrug resistance; MMP, matrix metalloproteinase; NPC, nasopharyngeal carcinoma; NR, not reported; NSCLC, non-small cell lung cancer; PARP, poly(ADP-ribose) polymerase; PCa, prostate cancer; PDX, patient-derived xenograft; P-gp, P-glycoprotein; PI3K/AKT/mTOR, phosphoinositide 3-kinase/protein kinase B/mechanistic target of rapamycin; ROS, reactive oxygen species; SBD, Scutellaria barbata D. Don; TAM, tumor-associated macrophage; TGF-β, transforming growth factor-β.

Supplementary Table S3. Preclinical evidence of immune and tumor microenvironment–related effects of SBD

Study ID	Intervention	Dose / Exposure conditions	Comparator	Main findings of the study
**Bao 2019[1]**	SBD flavonoids (SBF)	functional assays at 800 μg/mL for 24 h (flow cytometry, ELISA, WB, qPCR)	U937 untreated control; TAM control (co-culture without treatment)	2. TAM polarization ↓ (CD163↓, CD206↓); 3. Immunosuppressive cytokine secretion ↓ (IL-10↓, IL-12↓, IL-1β↓, TNF-α↓); 4. TLR4/MyD88/NF-κB signaling pathway activity ↓.
**Cao 2025[2]**	Quercetin	In vitro: 1.56–200 μM for 24–72h; In vivo: 50 mg/kg i.p. every other day × 20 doses	HGC-27 normal glucose cells; HGC-27-HG (high-glucose) control; vehicle-treated mice	5. EMT inhibition (E-cadherin↑, Vimentin↓, N-cadherin↓, Snail↓); 6. PI3K/AKT signaling pathway ↓ (p-PI3K↓, p-AKT↓, MYC↓).
**Chen 2017[3]**	SBD methanol extract (MESB)	In vitro: 0–5 mg/mL for up to 24 h; In vivo: 60 mg/kg intraperitoneal injection, 6 times/week for 15 days	Vehicle control; normal lung cells (MRC5)	7. ER stress activation ↑ (GRP78↑, HSP70↑, caspase-4 activation↑); 8. Mitochondrial dysfunction ↑ (MMP↓, ROS↑, cytochrome c release, Bcl-2↓, pro-apoptotic proteins↑); 9. Extrinsic apoptosis activation ↑ (FAS/FASL signaling); 10. P38/MAPK signaling ↑ and AKT signaling ↓ (p-P38↑, p-AKT↓); 11. SIRT1 expression ↓ contributing to apoptosis induction.
**Chen 2021[4]**	SBD methanol extract (MESB)	In vitro: 30–90 μg/mL for up to 48 h; In vivo: according to previous study	Vehicle control; normal epithelial cells	4. SOX2 identified as a direct molecular target; 5. Hedgehog signaling pathway ↓ (SOX2↓, SMO↓, GLI1↓).
**Chen 2024[5]**	SBD oil (SBO)	25–100 μg/mL for 48 h	Vehicle control	3. Caspase cascade activation ↑ (caspase-8↑, caspase-9↑, caspase-3↑); 4. Mitochondrial apoptosis pathway activation ↑ (Bax↑, Bcl-2↓); 5. IL-6/STAT3 signaling pathway ↓ (p-STAT3↓, IL-6-induced activation↓).

**Ding 2025[6]**	Scutellarin (SCU)	10, 25, 50 μM for 48 h (Western blot for METTL5 expression);	Untreated control (vehicle)	5. METTL5 expression ↓ (notably at 50 μM); 6. METTL5 overexpression rescues Scutellarin-induced migration inhibition (functional validation of target).
**Feng 2021[7]**	Scutebarbatine A (SBT-A)	0–0.2 μM for 24 h (WB); Z-VAD-FMK 20 μM (2 h pre-treatment) + 0.4 μM SBT-A (24 h)	Vehicle control; SBT-A alone	8. Z-VAD-FMK partially rescued viability; 9. p-ERK, p-JNK, p-p38 ↑; 10. PERK, ATF4, CHOP ↑.
**Gao 2025[8]**	SBD alkaloid fraction (SBA)	In vitro: 10–20 mg/mL for 24 h (WB); In vivo: 25–50 mg/kg/day oral × 21 days + cisplatin (DDP) combination	Vehicle; DDP; SBA; combination	5. E-cadherin ↑ and N-cadherin ↓ (EMT inhibition); 6. MMP2 ↓ and MMP9 ↓; 7. p-p38 ↑ and p-p53 ↑; 10. SBA attenuated cisplatin-induced renal toxicity.
**Huang 2022[12]**	Scutellarin (SCU)	In vitro: various concentrations for 72 h (IC50 = 35.99 μM for HL-60, 45.26 μM for THP-1, 62.61 μM for KG-1); In vivo: breviscapine containing ≥90% scutellarin, 60 mg/kg/day orally for 21 days	Vehicle control (normal saline)	3. Z-VAD-FMK partially rescued apoptosis; 4. JNK/caspase-3 pathway activation confirmed in tumor tissue (p-JNK ↑, cleaved caspase-3 ↑, cleaved PARP ↑); 5. SP600125 (JNK inhibitor) partially reversed apoptosis and protein changes
**Jin 2017[13]**	SBD ethanol extract (EESB)	0.125–0.5 mg/mL for 24 h (WB)	Untreated control	4. p-PI3K ↓, PI3K ↓, p-AKT ↓, PTEN ↑; 5. TGF-β ↓, Smad2/3 ↓, Smad4 ↓
**Kan 2017[14]**	SBD extract (SBE)	In vitro: 0.05–0.5 mg/mL for 48 h; In vivo: 50–150 mg/kg/day × 30 days; IL-17A injection model	Vehicle; SBE; SBE + IL-17A	3. Enhanced antitumor immunity, evidenced by ↑ NK cell cytotoxicity, ↓ CD4+CD25+Foxp3+ Treg cells and CD4+IL-17+ Th17 cells in tumor tissue, ↓ Serum IL-10 and TGF-β, ↓ IL-17A, ↑ Serum IL-2 and IFN-γ); 4. IL-17A administration reversed anti-tumor effect.
**Li 2019a[15]**	SBD polysaccharides (SBPS)	In vivo: 50–200 mg/kg/day × 7 days (H22 hepatoma mice); CTX control included	Model; CTX; SBPS groups	3. Systemic proteomic alterations, evidenced by differential protein expression profiles (2-DE), including ↓ Pus1p and SRP9/14 and restoration of MRPL24
**Li 2019b[16]**	SBD polysaccharide SBPW3	In vitro: 25–800 μg/mL (CCK-8, 36 h); migration/invasion assays (±TGF-β1 10 ng/mL); WB (EMT	Control; TGF-β1-induced	2. TGF-β1-induced migration ↑ (model validation); 5. TGF-β1-induced EMT ↓ (E-cadherin ↑, N-cadherin ↓, α-SMA ↓); 6. Suppression of Smad-dependent signaling, indicated by ↓ p-

		markers); In vivo: 100–200 mg/kg (metastasis model, 31 days)	model; SBPW3-treated	Smad2 and p-Smad3, with no significant changes in PI3K/AKT signaling
**Li 2022[17]**	SBD extract	In vitro: HCC cell lines (SMMC-7721, HepG2, Huh7), 0–50 mg/mL, 48 h; LDH, EdU, JC-1, ROS, iron assay, WB, RT-PCR; In vivo: xenograft model (oral administration)	Scutellaria barbata extract	4. Ferroptosis-related alterations, evidenced by ↑ iron accumulation, mitochondrial dysfunction (↓ membrane potential), lipid ROS dysregulation, ↓ GPX4 and SLC7A11, and ↑ IREB2 and ACSL4, with consistent in vivo maintenance of ferroptosis markers
**Lin 2017[18]**	SBD ethanol extract (EESB)	HCT-8/5-FU cells, 0.5–1.5 mg/mL, 24–48 h; MTT, colony formation, apoptosis assay, Rh-123 assay, RT-PCR, WB	5-FU-resistant cells vs parental; untreated control	5. Inhibition of drug efflux, evidenced by ↑ Rh-123 accumulation and ↓ ABCG2 expression 6. Regulation of cell cycle and apoptosis, indicated by ↓ Cyclin D1, ↑ p21, ↓ Bcl-2, and ↑ Bax 7. PI3K ↓ and p-AKT ↓ (pathway inhibition)
**Liu 2018[19]**	Baicalein	In vitro: H1975 cells (5–20 μM, 24–48 h), RAW264.7 (LPS-induced); WB; In vivo: LLC tumor-bearing mice (1.5 mg/kg/day)	Control (vehicle)	4. Suppression of oncogenic signaling pathways, evidenced by ↓ p-AKT and ↓ p-ERK/p-p38; 5. Anti-inflammatory effects, evidenced by ↓ COX-2, iNOS, and NF-κB; 8. Enhanced antitumor immunity, evidenced by ↑ CD8+ T cells, ↓ Treg cells, and ↑ NK cell infiltration
**Liu 2019[20]**	Scutellarin (SCU)	In vitro: ESCC cells (KYSE30/450/510, 48 h; MTT, colony, WB, FACS); In vivo: PDX mice (100 mg/kg)	Control (vehicle), normal esophageal cells	1. suppression of AKT signaling, evidenced by ↓ AKT1/2 kinase activity, ↓ p-AKT, and ↓ GSK3β phosphorylation; 6. AKT-dependent mechanism supported by reduced cell growth upon AKT knockdown and diminished scutellarin effects in AKT-deficient cells;
**Liu 2022[21]**	SBD flavonoids (FSB)	In vitro: HCT116 (24 h, 75–300 μg/ml; MTT, EdU, apoptosis, migration, WB); In vivo: xenograft (100–200 mg/kg/day)	Control (vehicle), normal IEC-6 cells	3. ROS accumulation ↑; 5. Autophagy ↓, evidenced by ↓ LC3 puncta and LC3-II/I; 6. Dysregulation of AMPK/mTOR signaling, evidenced by ↓ p-AMPK and p-ULK1 and ↑ p-mTOR; 7. Downregulation of ATF4 signaling, evidenced by ↓ ATF4 and sestrin2; 8. ATF4-dependent mechanism supported by attenuation of FSB effects upon ATF4 overexpression;

**Ma 2024[22]**	Scutellarin (SCU) and SBD water extract (SBW)	In vitro: BCSCs (MTT, BrdU, sphere, colony, migration); In vivo: NOD/SCID mice (50–100 mg/kg)	Vehicle, paclitaxel	5. Wnt/β-catenin signaling ↓; 6. NF-κB signaling ↓; 7. ↓PTEN/Akt/mTOR signaling
**Niu 2024[23]**	SBD petroleum ether extract	500 μg/mL for 24 h (in vitro); EGF 50 ng/mL; GSK429286A 1 μM; oe-Ezrin transfection	Vehicle control; EGF (RhoA/ROCK1 activator); GSK429286A (RhoA/ROCK1 inhibitor); oe-Ezrin	6. SB ↓ p-RhoA & ↓ p-ROCK1; 7. SB ↓ Ezrin; 8. EGF ↑ p-RhoA, ↑ p-ROCK1, ↑ Ezrin, reversing SB effects; 9. GSK429286A ↓ ROCK1, ↓ p-ROCK1, ↓ Ezrin; RhoA/p-RhoA unchanged; 10. oe-Ezrin ↑ Ezrin without affecting RhoA/ROCK1 levels; 11. SB suppresses RhoA/ROCK1–Ezrin axis to inhibit TNBC invasion and metastasis.
**Niu 2025[24]**	Scutebarbatine B (SBT-B)	0.05–0.4 μM for 24 h (in vitro); SP600125 20 μM (JNK inhibitor, 1 h pretreatment); 0.1 mg/kg i.p. every 2 days (in vivo)	Vehicle control; SP600125 alone; SBT-B + SP600125 combination	8. SBT-B ↑ ROS; NAC reversed SBT-B-induced ↓ viability & ↑ cleaved caspase-8/PARP; 9. SBT-B ↓ pRB, ↓ E2F1, ↓ survivin; 10. SBT-B ↓ p-Akt & ↓ p-p70S6K (Akt/mTOR suppression); 11. SBT-B ↑ IRE1 & ↑ p-JNK; SP600125 reversed SBT-B-induced ↓ viability & ↑ cleaved PARP; 12. In vivo: ↑ ROS (DHE), ↑ p-JNK in tumor tissues.
**Ouyang 2025[25]**	Quercetin, an active component of SBD	ACT-1 thyroid cancer cells, 48 h; MTT, colony assay, apoptosis assay, RT-qPCR	Normal thyroid cells (Nthy-ori 3-1), control	4. SPP1 expression ↓, evidenced by ↓ SPP1 mRNA and protein levels
**Shan 2025[26]**	SBD selenium-polysaccharide (SBP-Se)	In vitro: HepG2 (CCK-8, migration, colony); In vivo: mouse xenograft	SBP, SeNPs, control	4. Suppression of PI3K/Akt/mTOR signaling, evidenced by ↓ p-PI3K, p-Akt, and p-mTOR
**Sheng 2022[27]**	SBD extract	PCa cells (PC-3, DU145), 24–48 h; in vivo xenograft; migration/invasion/angiogenesis assays	Control, MK22006 (AKT inhibitor)	3. Suppression of PI3K/AKT signaling, evidenced by ↓ p-PI3K and p-AKT; 4. Consistent pathway involvement confirmed by similar effects with AKT inhibitor treatment; 6. ↑ E-cadherin, ↓ N-cadherin and vimentin; 7. Angiogenesis ↓, evidenced by reduced HUVEC tube formation

**Shi 2019[28]**	Scutellarein	In vitro: MM cells (MM.1R, IM-9), 24 h; ROS, mitochondrial assay, WB; In vivo xenograft	In vitro: MM cells (MM.1R, IM-9), 24 h; ROS, mitochondrial assay, WB; In vivo xenograft	2. Activation of intrinsic (Apaf-1–dependent) apoptotic pathway; 4. ROS accumulation ↑, including increased mitochondrial superoxide; 5. ROS-dependent mechanism supported by reduced apoptosis upon MitoTEMPO treatment; 6. Partial contribution of NADPH oxidase to ROS
**Shi 2022[29]**	Baicalein, Wogonin	NPC cells (CNE2, 5-8F), 24–48 h; CCK-8, apoptosis assay, WB	Control, cisplatin	2. PI3K/AKT signaling ↓ evidenced by ↓ PI3K and AKT; 3. p53 expression ↓
**Shim 2016[30]**	SBD ethanol extract (ESB)	MKN-45 cells, 24–72 h; MTT, FACS (sub-G1), caspase assay, JC-1, ROS assay, MAPK inhibitors, NAC	Control, MAPK inhibitors, NAC, chemo drugs	3. ROS accumulation ↑, with reduced apoptosis following NAC treatment; 4. MAPK-dependent mechanism supported by reduced apoptosis upon ERK, p38, and JNK inhibition; 5. No comparable effect observed with PI3K/AKT inhibition
**Su 2022[31]**	SBD acidic polysaccharide SBP-2A	HepG2 cells, 50–3200 μg/mL, 48 h; MTT, colony formation, EdU, flow cytometry, qPCR, WB	Control	4. Regulation of cell cycle–related signaling, evidenced by ↑ p53 and ↓ Cyclin D1/CDK4
**Sun 2017[32]**	SBD polysaccharide SPS2p	HT29 cells, 10–80 mg/L, 24–96 h; MTT, apoptosis assay, WB, qPCR, ELISA	Control	3. epithelial–mesenchymal transition (EMT) ↓, as indicated by E-cadherin ↑ and N-cadherin ↓, vimentin ↓, and fibronectin (FN) ↓; 4. PI3K/AKT signaling ↓ (p-AKT/AKT ↓).
**Wang 2024[38]**	SBD diterpenoid Bzl-D	5–40 μg/mL for 24 h (CCK-8, apoptosis, migration/invasion assays); in vivo 50 mg/kg/day ×16 days	Control (vehicle/saline)	5. Hedgehog (HH) signaling ↓, as indicated by SHH ↓, PTCH ↓, SMO ↓, and GLI ↓, with SHH binding ↑
**Wang 2025[39]**	Baicalin, Wogonin	0–10 mg/mL, 24–72 h (CCK-8, Transwell, TUNEL, RT-PCR, WB)	Untreated control	4. epithelial–mesenchymal transition (EMT) ↓; 5. oncogenic and inflammatory signaling ↓, as indicated by EGFR ↓, TNF-α ↓, VEGFA ↓, PI3K ↓, AKT ↓, MDM2 ↓, and PI3K/AKT signaling ↓;
**Xue 2016[41]**	SBD-derived neoclerodane diterpenoids	Cytotoxicity screening: 50 μM (MCF-7 cells); MDR reversal assay: 20 μM (MCF-7/ADR	Untreated control; Positive control:	4. P-glycoprotein (P-gp) activity ↓ and expression ↓, indicating inhibition of drug efflux; 5. structure–activity relationship (SAR)

		cells); Additional assays: 10–20 μM (ADR accumulation, Rh123 assay, Western blot, etc.)	verapamil (P-gp inhibitor)	analysis revealed that ester moieties (especially benzoyloxy substitution) ↑ MDR reversal activity.
**Yang 2017a[43]**	Scutellarin (SCU)	10, 30, 100 μM for 24–48 h (WB for p53, p21, Bcl-2, Bax, caspase-3)	DMSO control	7. p53/p21 pathway activation: p-p53↑ and p21↑ dose-dependently; p53-dependent apoptosis mechanism confirmed by pifithrin-α rescue experiment (Bcl-2↑, Bax↓, active caspase-3↓ upon p53 inhibition).
**Yang 2025a[47]**	BZL-5 (barbatin A)	8 μM for 48 h (qRT-PCR for STAT3, HSP90AA1, HIF1A, NFKB1, TLR4, PIK3CA); molecular docking and molecular dynamics simulation	Untreated control	6. mRNA expression ↓ for STAT3, HSP90AA1, HIF1A, NFKB1, TLR4, PIK3CA at 8 μM ($p < 0.05$); 7. HIF-1 signaling pathway identified as key mechanism: BZL-5 showed strongest binding affinity to HIF1A (docking score −9.989 kcal/mol); 8. Molecular dynamics simulation confirmed stable BZL-5/HIF1A complex binding; 9. Key enriched pathways: PI3K-Akt signaling pathway, HIF-1 signaling pathway, Ras signaling pathway, cellular senescence.
**Yang 2025b[48]**	Luteolin, a flavonoid isolated from SBD	20 μM for 24 h; LV-oe-miR-6809-5p and/or LV-oe-FLOT1 transfection	Vehicle control (DMSO); LV-miR-NC; LV-oe-FLOT1	5. Luteolin ↓ miR-6809-5p (aberrantly upregulated in HCC); 6. Luteolin ↓ FLOT1 & ↓ p-AKT; miR-6809-5p OE restored both; 7. Luteolin ↓ FAK, ↓ PI3K, ↓ AKT, ↓ mTOR mRNA; 8. Luteolin ↑ E-cadherin, ↑ β-catenin, ↓ Vimentin, ↓ N-cadherin, ↓ Snail, ↓ Twist, ↓ Slug (EMT suppression); miR-6809-5p OE & FLOT1 OE reversed EMT changes.
**Yue 2021[49]**	SBD water extract (SBW) and scutellarin (SCU)	In vivo: SBW 615 mg/kg, Scu 7 mg/kg for 4 weeks; Western blot on HCT116 xenograft tumor tissue; flow cytometry on spleens and tumors of Colon26-Luc mice	Untreated control	8. Apoptosis marker expression ↑ in tumor tissue: cleaved caspase-3 ↑, cleaved PARP ↑, p53 ↑; 9. Metastasis-related protein modulation: E-cadherin ↑ ($p<0.01$), CXCR4 ↓ (trend), Tspan8 ↓ in tumor tissue; 10. Signaling pathway suppression: p-ERK ↓, β-catenin ↓ (Scu-H), Src kinase ↓ (SBW-L); 11. MDSC (myeloid-derived suppressor cell) population ↓ in spleens of

				SBW-L-treated mice (p<0.01); MDSC population ↓ in tumors of Scu-H-treated mice (not significant).
**Zeng 2021[50]**	Scutellarin (SCU)	In vitro: 90, 180, 360 μM for 48 h (WB, qRT-PCR, ICC for Wnt pathway); In vivo: 25, 50, 100 mg/kg/day i.p. (IHC, WB, qRT-PCR on CAC tissue; ELISA on serum)	Vehicle control; model group	8. TNF-α ↓ and IL-6 ↓ in serum of CAC mice dose-dependently (ELISA); 9. Wnt/β-catenin signaling ↓ in CAC tissue and HT-29 cells: total β-catenin ↓ and nuclear β-catenin ↓; 10. GSK-3β ↑ and p-GSK-3β (Ser9) ↓, indicating restoration of GSK-3β activity and enhanced β-catenin phosphorylation/degradation; 11. TCF4 ↓, c-Myc ↓, cyclin D1 ↓ (downstream Wnt target genes) at mRNA and protein level in both CAC tissue and HT-29 cells.
**Zhang 2017b[52]**	SBD chloroform fraction (ECSB)	150 μg/mL for 48 h (qRT-PCR, WB); anti-miR-34a oligonucleotide (AMO-34a) transfection + ECSB 150 μg/mL rescue experiment	Untreated control; scrambled oligonucleotide negative control	5. miR-34a expression ↑ after ECSB treatment (qRT-PCR); 6. Notch1 ↓, Notch2 ↓, Jagged1 ↓ at mRNA and protein level (miR-34a downstream targets); 7. AMO-34a transfection reversed miR-34a upregulation → Notch1/2, Bcl-2, Jagged1 ↑; ECSB co-treatment rescued this effect, confirming miR-34a/Notch signaling as the direct mechanism of ECSB anti-tumor action.
**Zhang 2023[53]**	Quercetin, luteolin, and wogonin, active components of SBD	IC50 concentrations for 48 h (quercetin 121.43 μmol/L, luteolin 95.27 μmol/L, wogonin 115.57 μmol/L)	Vehicle control (DMSO)	3. Quercetin, luteolin, and wogonin ↓ AKT1 mRNA; 4. Quercetin, luteolin, and wogonin ↓ VEGFA mRNA; 5. Quercetin, luteolin, and wogonin ↓ JUN mRNA; 6. Quercetin, luteolin, and wogonin ↓ TNF mRNA; 7. Quercetin, luteolin, and wogonin ↓ PI3K protein & ↓ p-Akt; ↑ Caspase-3 protein.
**Zhang 2025[55]**	SBD aqueous extract (SBE), with wogonin as an active ingredient	0, 240, 360 μg/mL for 24 h (WB for CDK1); network pharmacology + bioinformatics (TCMSP, GEPIA2, UALCAN, STRING, GeneMANIA, TIMER 2.0)	Untreated control; LUAD vs. normal tissue (database analysis)	5. CDK1 protein expression ↓ after SBE treatment in A549 cells (Western blot); 6. CDK1 overexpressed in LUAD vs. normal tissue (GEPIA2, UALCAN); high CDK1 expression correlated with advanced pathological stage (I→IV) and poor prognosis (OS↓, DFS↓); 7. CDK1-interacting genes identified: CKS1B, CKS2, CDC25C, CCNB1, CCNB2; all positively correlated with CDK1 expression in LUAD (TIMER 2.0); 8. Key enriched pathways: cell

				cycle regulation (G2/M phase transition), progesterone-mediated oocyte maturation, p53 signaling pathway.
**Zheng 2018[56]**	Total flavonoids of SBD (TF-SB)	In vitro: 40–200 μg/mL for 24–72 h; In vivo: 50, 100, 200 mg/kg by gavage for 4 weeks	Vehicle control (distilled water)	5. TF-SB ↓ PTHrP mRNA & protein dose-dependently; 6. TF-SB ↓ RANKL mRNA & protein dose-dependently; 7. TF-SB ↑ OPG mRNA & protein dose-dependently; 8. TF-SB suppresses breast cancer bone metastasis–induced osteolysis via PTHrP/RANKL/OPG axis modulation in the bone microenvironment.

AKT, protein kinase B; AMPK, AMP-activated protein kinase; EMT, epithelial–mesenchymal transition; ER stress, endoplasmic reticulum stress; ESCC, esophageal squamous cell carcinoma; HCC, hepatocellular carcinoma; JNK, c-Jun N-terminal kinase; LUAD, lung adenocarcinoma; MAPK, mitogen-activated protein kinase; MDR, multidrug resistance; MDSC, myeloid-derived suppressor cell; NF-κB, nuclear factor kappa B; NK, natural killer; NPC, nasopharyngeal carcinoma; PARP, poly(ADP-ribose) polymerase; PCa, prostate cancer; PDX, patient-derived xenograft; P-gp, P-glycoprotein; PI3K/AKT/mTOR, phosphoinositide 3-kinase/protein kinase B/mechanistic target of rapamycin; ROS, reactive oxygen species; SBD, Scutellaria barbata D. Don; STAT3, signal transducer and activator of transcription 3; TAM, tumor-associated macrophage; TGF-β, transforming growth factor-β; TNBC, triple-negative breast cancer; Treg, regulatory T cell

Supplementary Table S4. Active candidate compounds of *Scutellaria barbata* used in the compound–target network, with their major chemical classes and SMILES representations.

Compound ID	Compound name	Major chemical class	SMILES representation
**C1**	(2R)-5,7-dihydroxy-2-(4-hydroxyphenyl)chroman-4-one	Flavonoid	C1[C@@H](OC2=CC(=CC(=C2C1=O)O)O)C3=CC=C(C=C3)O
**C2**	5,7,4'-trihydroxy-6-methoxyflavanone	Flavonoid	COC1=C(C2=C(C=C1O)O[C@H](CC2=O)C3=CC=C(C=C3)O)O
**C3**	5,7,4'-trihydroxy-8-methoxyflavanone	Flavonoid	COC1=C(C=C(C2=C1OC(CC2=O)C3=CC=C(C=C3)O)O)O
**C4**	5-hydroxy-7,8-dimethoxy-2-(4-methoxyphenyl)chromone	Flavonoid	COC1=CC=C(C=C1)C2=CC(=O)C3=C(O2)C(=C(C=C3O)OC)OC
**C5**	7-hydroxy-5,8-dimethoxy-2-phenyl-chromone	Flavonoid	COC1=C2C(=O)C=C(OC2=C(C(=C1)O)OC)C3=CC=CC=C3
**C6**	Chrysin-5-methylether	Flavonoid	COC1=CC(=CC2=C1C(=O)C=C(O2)C3=CC=CC=C3)O
**C7**	9,19-cyclolanost-24-en-3-ol	Sterol/triterpenoid derivative	C[C@H](CCC=C(C)C)[C@H]1CC[C@@]2([C@@]1(CC[C@]34[C@H]2CC[C@H]5[C@]3(C4)CCC(C5(C)C)O)C)C
**C8**	Baicalin	Flavonoid	C1=CC=C(C=C1)C2=CC(=O)C3=C(C(=C(C=C3O2)O[C@H]4[C@@H]([C@H]([C@@H]([C@H](O4)C(=O)O)O)O)O)O)O
**C9**	campesterol	Sterol/triterpenoid derivative	C[C@H](CC[C@@H](C)C(C)C)[C@H]1CC[C@@H]2[C@@]1(CC[C@H]3[C@H]2CC=C4[C@@]3(CC[C@@H](C4)O)C)C
**C10**	CLR	Sterol/triterpenoid derivative	C[C@H](CCCC(C)C)[C@H]1CC[C@@H]2[C@@]1(CC[C@H]3[C@H]2CC=C4[C@@]3(CC[C@@H](C4)O)C)C
**C11**	beta-sitosterol	Sterol/triterpenoid derivative	CC[C@H](CC[C@@H](C)[C@H]1CC[C@@H]2[C@@]1(CC[C@H]3[C@H]2CC=C4[C@@]3(CC[C@@H](C4)O)C)C)C(C)C
**C12**	rivularin	Flavonoid	COC1=CC=CC(=C1C2=CC(=O)C3=C(O2)C(=C(C=C3O)OC)OC)O
**C13**	Sitosteryl acetate	Sterol/triterpenoid derivative	CC[C@H](CC[C@@H](C)[C@H]1CC[C@@H]2[C@@]1(CC[C@H]3[C@H]2CC=C4[C@@]3(CC[C@@H](C4)OC(=O)C)C)C)C(C)C
**C14**	Stigmasta-5,22-dien-3-ol-acetate	Sterol/triterpenoid derivative	CC[C@H](C=C[C@@H](C)[C@H]1CC[C@@H]2[C@@]1(CC[C@H]3[C@H]2CC

			=C4[C@@]3(CCC(C4)OC(=O)C)C)C)C(C)C
**C15**	Stigmastan-3,5,22-triene	Sterol/triterpenoid derivative	CCC(/C=C/C(C)C1CCC2C1(CCC3C2CC=C4C3(CCC=C4)C)C)C(C)C
**C16**	Stigmasterol	Sterol/triterpenoid derivative	CC[C@H](/C=C/[C@@H](C)[C@H]1CC[C@@H]2[C@@]1(CC[C@H]3[C@H]2CC=C4[C@@]3(CC[C@@H](C4)O)C)C)C(C)C
**C17**	wogonin	Flavonoid	COC1=C(C=C(C2=C1OC(=CC2=O)C3=CC=CC=C3)O)O
**C18**	Dinatin	Flavonoid	COC1=C(C2=C(C=C1O)OC(=CC2=O)C3=CC=C(C=C3)O)O
**C19**	24-Ethylcholest-4-en-3-one	Sterol/triterpenoid derivative	CC[C@H](CC[C@@H](C)[C@H]1CC[C@@H]2[C@@]1(CC[C@H]3[C@H]2CCC4=CC(=O)CC[C@]34C)C)C(C)C
**C20**	baicalein	Flavonoid	C1=CC=C(C=C1)C2=CC(=O)C3=C(O2)C=C(C(=C3O)O)O
**C21**	6-Hydroxynaringenin	Flavonoid	C1[C@H](OC2=C(C1=O)C(=C(C(=C2)O)O)O)C3=CC=C(C=C3)O
**C22**	Salvigenin	Flavonoid	COC1=CC=C(C=C1)C2=CC(=O)C3=C(C(=C(C=C3O2)OC)OC)O
**C23**	Rhamnazin	Flavonoid	COC1=CC(=C2C(=C1)OC(=C(C2=O)O)C3=CC(=C(C=C3)O)OC)O
**C24**	sitosterol	Sterol/triterpenoid derivative	CC[C@H](CC[C@@H](C)[C@H]1CC[C@@H]2[C@@]1(CC[C@H]3[C@H]2CC=C4[C@@]3(CC[C@H](C4)O)C)C)C(C)C
**C25**	eriodictyol	Flavonoid	C1[C@H](OC2=CC(=CC(=C2C1=O)O)O)C3=CC(=C(C=C3)O)O
**C26**	daucostero_qt	Sterol/triterpenoid derivative	CC[C@@H](CC[C@@H](C)[C@@H]1CC[C@@H]2[C@]1(CC[C@@H]3[C@@H]2CC=C4[C@@]3(CC[C@@H](C4)O)C)C)C(C)C
**C27**	luteolin	Flavonoid	C1=CC(=C(C=C1C2=CC(=O)C3=C(C=C(C=C3O2)O)O)O)O
**C28**	Moslosooflavone	Flavonoid	COC1=C(C2=C(C(=C1)O)C(=O)C=C(O2)C3=CC=CC=C3)OC
**C29**	quercetin	Flavonoid	C1=CC(=C(C=C1C2=C(C(=O)C3=C(C=C(C=C3O2)O)O)O)O)O
**C30**	Scutellarein	Flavonoid	C1=CC(=CC=C1C2=CC(=O)C3=C(O2)C=C(C(=C3O)O)O)O
**C31**	Scutellarin	Flavonoid	C1=CC(=CC=C1C2=CC(=O)C3=C(C(=C(C=C3O2)O[C@H]4[C@@H]([C@H]([C@@H]([C@H](O4)C(=O)O)O)O)O)O)O)O
**C32**	Pheophorbide a	Chlorophyll-derived tetrapyrrole	C=Cc1c(C)c2cc3nc(c4c5[nH]c(cc6nc(cc1[nH]2)C(C)=C6CC)c(C)c5C(=O)[C@@H]4C(=O)OC)[C@@H](CCC(=O)O)[C@@H]3C

Supplementary Table S5. Cross-tabulation of network-derived hub targets and candidate compounds with experimental evidence from the included studies.

Category	Network-derived item	Experimental overlap in included studies, n
**Hub target**	TNF	4
	AKT1	2
	SRC	1
	EGFR	2
	BCL2	19
	HIF1A	1
	MAPK3	0
	HSP90AA1	1
	ESR1	0
	MMP9	5
**Candidate compound**	Baicalin	1
	Baicalein	2
	Wogonin	4
	Rhamnazin	1
	Luteolin	4
	Quercetin	3
	Scutellarein	2
	Scutellarin	9

For hub targets, experimental overlap was counted only when the corresponding gene or protein was explicitly measured in an included study. For candidate compounds, overlap was counted only when the compound was directly evaluated as an experimental intervention. The remaining 24 network-derived candidate compounds had no direct intervention-level match in the included studies.

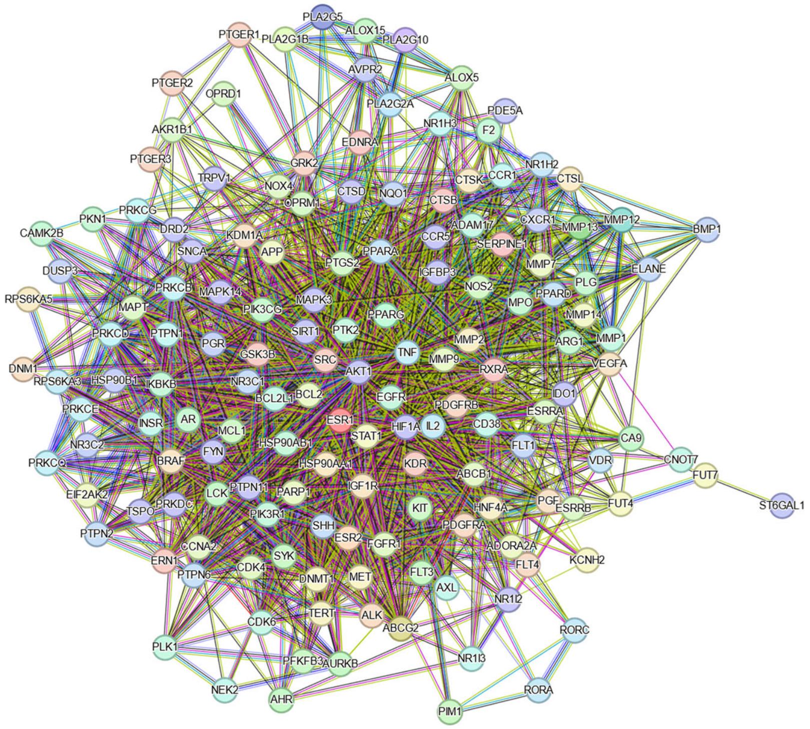

Supplementary Figure S1. Complete STRING PPI network comprising 152 immune/TME-overlapping SBD targets. The network was constructed using the 152 immune/TME-overlapping targets of Scutellaria barbata. Node labels indicate gene symbols. Node size is uniform, and node colors are used only for visual distinction. Edge colors represent STRING evidence channels: light blue, curated databases; purple, experimental evidence; green, gene neighborhood; red, gene fusion; dark blue, gene co-occurrence; yellow, text mining; and black, co-expression. Major hub targets included AKT1, TNF, SRC, EGFR, and BCL2.

References

49. Bao, X.; Li, L.; Xue, X. Flavonoids from *Scutellaria barbata* inhibit activation of tumor-associated macrophages by blocking the Toll-like receptor 4/myeloid differentiation factor 88/nuclear factor-κB signaling pathway. *J. Tradit. Chin. Med.* **2019**, *39*, 160–165.
56. Cao, H.; Song, H.; Zhou, W.; Lv, X.; Liu, X.; Xiang, Z.; Fu, R.; Cheng, Y.; Chen, J.; Wang, S.; et al. Exploring the active ingredients of Banzhilian and its mechanism of action on diabetic Gastric cancer based on network pharmacology. *Sci. Rep.* **2025**, *15*, 14808.
40. Chen, C.-C.; Kao, C.-P.; Chiu, M.-M.; Wang, S.-H. The anti-cancer effects and mechanisms of *Scutellaria barbata* D. Don on CL1-5 lung cancer cells. *Oncotarget* **2017**, *8*, 109340–109357.
36. Chen, W.-W.; Gong, K.-K.; Yang, L.-J.; Dai, J.-J.; Zhang, Q.; Wang, F.; Li, X.-L.; Xi, S.-C.; Du, J. *Scutellaria barbata* D. Don extraction selectively targets stemness-prone NSCLC cells by attenuating SOX2/SMO/GLI1 network loop. *J. Ethnopharmacol.* **2021**, *265*, 113295.
53. Chen, Y.-Y.; Zhu, Q.; Zhu, X.-L.; Li, H.-H.; Li, F.-Q. Study on Chemical Constituents and Cytotoxicity of *Scutellaria barbata* D. Don Oil Against SMMC-7721. *Acta Pol. Pharm.-Drug Res.* **2024**, *81*, 831–839.
55. Ding, L.; Luo, C.; Weygant, N.; Chen, W.; Ru, D.; Lai, Y.; Wang, Y.; Li, H. Scutellarin suppresses ovarian cancer progression by targeting METTL5. *Sci. Rep.* **2025**, *15*, 18472.
71. Feng, P.-P.; Qi, Y.-K.; Li, N.; Fei, H.-R. Scutebarbatine A induces cytotoxicity in hepatocellular carcinoma via activation of the MAPK and ER stress signaling pathways. *J. Biochem. Mol. Toxicol.* **2021**, *35*, e22731.
50. Gao, B.; Sui, X.; Choe, H.; Yang, Y.; Liu, Y.; Xia, L.; Li, P.; Chen, Y.; Huo, Y.; Zhao, Z.; et al. Alkaloids isolated from *Scutellaria barbata* D. Don trigger apoptosis and inhibit migration by modulating the p38-p53 pathway in ovarian cancer. *J. Ovarian Res.* **2025**, *18*, 301.
73. Guo, F.; Yang, F.; Zhu, Y.-H. Scutellarein from *Scutellaria barbata* induces apoptosis of human colon cancer HCT116 cells through the ROS-mediated mitochondria-dependent pathway. *Nat. Prod. Res.* **2019**, *33*, 2372–2375.
64. Hanh, T.T.H.; Anh, D.H.; Quang, T.H.; Trung, N.Q.; Thao, D.T.; Cuong, N.T.; An, N.T.; Cuong, N.X.; Nam, N.H.; Kiem, P.V.; et al. Scutebarbatolides A-C, new neo-clerodane diterpenoids from *Scutellaria barbata* D. Don with cytotoxic activity. *Phytochem. Lett.* **2019**, *29*, 65–69.
37. Hu, Z.; Jing, D. Anti-proliferation effect of *Scutellaria barbata* D. Don extract on lung cancer. *Trop. J. Pharm. Res.* **2019**, *18*, 1867–1872.
58. Huang, Z.; Yang, Y.; Fan, X.; Ma, W. Network pharmacology-based investigation and experimental validation of the mechanism of scutellarin in the treatment of acute myeloid leukemia. *Front. Pharmacol.* **2022**, *13*, 952677.
15. Jin, Y.; Chen, W.; Yang, H.; Yan, Z.; Lai, Z.; Feng, J.; Peng, J.; Lin, J. *Scutellaria barbata* D. Don inhibits migration and invasion of colorectal cancer cells via suppression of PI3K/AKT and TGF-β/Smad signaling pathways. *Exp. Ther. Med.* **2017**, *14*, 5527–5534.
11. Kan, X.; Zhang, W.; You, R.; Niu, Y.; Guo, J.; Xue, J. *Scutellaria barbata* D. Don extract inhibits the tumor growth through down-regulating of Treg cells and manipulating Th1/Th17 immune response in hepatoma H22-bearing mice. *BMC Complement. Altern. Med.* **2017**, *17*, 41.
45. Li, L.; Xu, X.; Wu, L.; Zhu, H.; He, Z.; Zhang, B.; Chi, Y.; Song, G. *Scutellaria barbata* polysaccharides inhibit tumor growth and affect the serum proteomic profiling of hepatoma H22-bearing mice. *Mol. Med. Rep.* **2019**, *19*, 2254–2262.
44. Li, H.; Su, J.; Jiang, J.; Li, Y.; Gan, Z.; Ding, Y.; Li, Y.; Liu, J.; Wang, S.; Ke, Y. Characterization of polysaccharide from *Scutellaria barbata* and its antagonistic effect on the migration and invasion of HT-29 colorectal cancer cells induced by TGF-β1. *Int. J. Biol. Macromol.* **2019**, *131*, 886–895.
35. Li, Y.; Zhang, J.; Zhang, K.; Chen, Y.; Wang, W.; Chen, H.; Zou, Z.; Li, Y.; Dai, M. *Scutellaria barbata* Inhibits Hepatocellular Carcinoma Tumorigenicity by Inducing Ferroptosis of Hepatocellular Carcinoma Cells. *Front. Oncol.* **2022**, *12*, 693395.
12. Lin, J.; Feng, J.; Yang, H.; Yan, Z.; Li, Q.; Wei, L.; Lai, Z.; Jin, Y.; Peng, J. *Scutellaria barbata* D. Don inhibits 5-fluorouracil resistance in colorectal cancer by regulating PI3K/AKT pathway. *Oncol.*

Rep. **2017**, *38*, 2293–2300.

74. Liu, J.; Jiang, M.; Li, Z.; Zhang, X.; Li, X.; Hao, Y.; Su, X.; Zhu, J.; Zheng, C.; Xiao, W.; et al. A Novel Systems Pharmacology Method to Investigate Molecular Mechanisms of *Scutellaria barbata* D. Don for Non-small Cell Lung Cancer. *Front. Pharmacol.* **2018**, *9*, 1473.
57. Liu, F.; Zu, X.; Xie, X.; Zhang, Y.; Liu, K.; Chen, H.; Wang, T.; Bode, A.M.; Dong, Z.; Kim, D.J. Scutellarin Suppresses Patient-Derived Xenograft Tumor Growth by Directly Targeting AKT in Esophageal Squamous Cell Carcinoma. *Cancer Prev. Res.* **2019**, *12*, 849–860.
51. Liu, L.; Liu, T.; Tao, W.; Liao, N.; Yan, Q.; Li, L.; Tan, J.; Shen, W.; Cheng, H.; Sun, D. Flavonoids from *Scutellaria barbata* D. Don exert antitumor activity in colorectal cancer through inhibited autophagy and promoted apoptosis via ATF4/sestrin2 pathway. *Phytomedicine* **2022**, *99*, 154007.
60. Ma, H.; Yue, G.G.; Lee, J.K.-M.; Gao, S.; Yuen, K.-K.; Cheng, W.; Li, X.; Lau, C.B. Scutellarin, a flavonoid compound from *Scutellaria barbata*, suppresses growth of breast cancer stem cells in vitro and in tumor-bearing mice. *Phytomedicine* **2024**, *128*, 155418.
41. Niu, J.; Hu, J.; Wang, Z. *Scutellaria barbata* D.Don extract regulates Ezrin-mediated triple negative breast cancer progress via suppressing the RhoA /ROCK1 signaling. *Toxicol. Res.* **2024**, *13*, tfae033.
70. Niu, C.; Li, R.-T.; Hao, X.-S.; Qi, X.; Wang, F.-Z.; Fei, H.-R. Scutebarbatine B Exerts Anti-Breast Cancer Activity by Inducing Cell Cycle Arrest and Apoptosis Through Multiple Pathways. *Phytother. Res.* **2025**, *39*, 3432–3449.
17. Ouyang, G.; Zhu, Y.; Ouyang, Z. Investigation of *Scutellaria barbata*'s immunological mechanism against thyroid cancer using network pharmacology and experimental validation. *Sci. Rep.* **2025**, *15*, 2490.
47. Shan, R.; Xu, X.; Yin, Y.; Liang, J.; Yuan, H.; Gao, X.; Zhao, Q.; Song, G. Selenium nanoparticles conjugated with *Scutellaris barbata* D. Don polysaccharides: Synthesis, optimization, structural characterization and evaluation of anti-hepatoma activity in vitro and in vivo. *Int. J. Biol. Macromol.* **2025**, *319*, 145196.
13. Sheng, D.; Zhao, B.; Zhu, W.; Wang, T.; Peng, Y. *Scutellaria barbata* D. Don (SBD) extracts suppressed tumor growth, metastasis and angiogenesis in Prostate cancer via PI3K/Akt pathway. *BMC Complement. Med. Ther.* **2022**, *22*, 120.
75. Shi, L.; Wu, Y.; Lv, D.L.; Feng, L. Scutellarein selectively targets multiple myeloma cells by increasing mitochondrial superoxide production and activating intrinsic apoptosis pathway. *Biomed. Pharmacother.* **2019**, *109*, 2109–2118.
61. Shi, H.; Liu, J.; Fan, J.; He, L.; Wang, X.; Tang, F.; Tian, D.; He, Y. Molecular Assessment of *Scutellaria barbata* D. Don in the Treatment of Nasopharyngeal Carcinoma Based on Network Pharmacology and Experimental Verification. *Evid.-Based Complement. Altern. Med.* **2022**, *2022*, 1988378.
14. Shim, J.H.; Gim, H.; Lee, S.; Kim, B.J. Inductions of Caspase-, MAPK- and ROS-dependent Apoptosis and Chemotherapeutic Effects Caused by an Ethanol Extract of *Scutellaria barbata* D. Don in Human Gastric Adenocarcinom Cells. *J. Pharmacopunct.* **2016**, *19*, 129–136.
48. Su, W.; Wu, L.; Liang, Q.; Lin, X.; Xu, X.; Yu, S.; Lin, Y.; Zhou, J.; Fu, Y.; Gao, X.; et al. Extraction Optimization, Structural Characterization, and Anti-Hepatoma Activity of Acidic Polysaccharides from *Scutellaria barbata* D. Don. *Front. Pharmacol.* **2022**, *13*, 827782.
46. Sun, P.; Sun, D.; Wang, X. Effects of *Scutellaria barbata* polysaccharide on the proliferation, apoptosis and EMT of human colon cancer HT29 Cells. *Carbohydr. Polym.* **2017**, *167*, 90–96.
76. Tan, L.; Lei, N.; He, M.; Zhang, M.; Sun, Q.; Zeng, S.; Chen, L.; Zhou, L.; Meng, X.; Xu, H. Scutellarin protects against human colorectal cancer in vitro by down regulation of hedgehog signaling pathway activity. *Int. J. Pharmacol.* **2020**, *16*, 53–62.
77. Wang, Q.; Acharya, N.; Liu, Z.; Zhou, X.; Cromie, M.; Zhu, J.; Gao, W. Enhanced anticancer effects of *Scutellaria barbata* D. Don in combination with traditional Chinese medicine components on non-small cell lung cancer cells. *J. Ethnopharmacol.* **2018**, *217*, 140–151.
66. Wang, M.; Ma, C.; Chen, Y.; Li, X.; Chen, J. Cytotoxic Neo-Clerodane Diterpenoids from

Scutellaria barbata D.Don. *Chem. Biodivers.* **2019**, *16*, e1800499.
54. Wang, L.; Xu, J.; Yan, Y.; Liu, H.; Karunakaran, T.; Li, F. Green synthesis of gold nanoparticles from *Scutellaria barbata* and its anticancer activity in pancreatic cancer cell (PANC-1). *Artif. Cells Nanomed. Biotechnol.* **2019**, *47*, 1617–1627.
65. Wang, M.; Chen, Y.; Hu, P.; Ji, J.; Li, X.; Chen, J. Neoclerodane diterpenoids from *Scutellaria barbata* with cytotoxic activities. *Nat. Prod. Res.* **2020**, *34*, 1345–1351.
69. Wang, W.; Tao, Z.; Tu, L.; Huang, W.; Ye, Y.; Gao, L.; Li, X. Diterpenoids from *Scutellaria barbata* D. Don Exert the Antiproliferative Effect on Colorectal Cancer Cells via Hedgehog Signaling Pathway. *Pharmacogn. Mag.* **2024**, *20*, 877–886.
78. Wang, G.; Jia, C.; Wang, D.; Liang, L.; Li, L.; Tian, G.; Feng, W. *Scutellaria barbata* inhibits epithelial-mesenchymal transformation through PI3K/AKT and MDM2 thus inhibiting the proliferation, migration and promoting apoptosis of Cervical Cancer cells. *PLoS ONE* **2025**, *20*, e0321556.
39. Wei, L.-H.; Lin, J.-M.; Chu, J.-F.; Chen, H.-W.; Li, Q.-Y.; Peng, J. *Scutellaria barbata* D. Don inhibits colorectal cancer growth via suppression of Wnt/β-catenin signaling pathway. *Chin. J. Integr. Med.* **2017**, *23*, 858–863.
79. Xue, G.-M.; Xia, Y.Z.; Wang, Z.-M.; Li, L.-N.; Luo, J.-G.; Kong, L.-Y. neo-Clerodane diterpenoids from *Scutellaria barbata* mediated inhibition of P-glycoprotein in MCF-7/ADR cells. *Eur. J. Med. Chem.* **2016**, *121*, 238–249.
42. Xue, S.; Geng, A.; Lian, T.; Liu, Y. *Scutellaria barbata* D. Don inhibits cervical cancer cell proliferation, migration, and invasion via miR-195-5p/LOXL2 axis. *Toxicol. Res.* **2022**, *11*, 804–811.
72. Yang, N.; Zhao, Y.; Wang, Z.; Liu, Y.; Zhang, Y. Scutellarin suppresses growth and causes apoptosis of human colorectal cancer cells by regulating the p53 pathway. *Mol. Med. Rep.* **2017**, *15*, 929–935.
68. Yang, G.-C.; Liang, C.; Li, S.-G.; Liu, M.; Jia, M.-J.; Xu, X.-N.; Wang, X.-B.; Hua, H.-M.; Sun, L.-X. Neoclerodane diterpenoids from aerial parts of *Scutellaria barbata*. *Phytochem. Lett.* **2017**, *19*, 1–6.
67. Yang, G.-C.; Hu, J.-H.; Li, B.-L.; Liu, H.; Wang, J.-Y.; Sun, L.-X. Six New neo-Clerodane Diterpenoids from Aerial Parts of *Scutellaria barbata* and Their Cytotoxic Activities. *Planta Med.* **2018**, *84*, 1292–1299.
16. Yang, A.-Y.; Liu, H.-L.; Yang, Y.-F. Study on the mechanism of action of *Scutellaria barbata* on hepatocellular carcinoma based on network pharmacology and bioinformatics. *Front. Pharmacol.* **2022**, *13*, 1072547.
18. Yang, Y.; Cheng, X.; He, Y.; Chen, T.; Chen, D.; Wang, A. Mechanisms of diterpenes from *Scutellaria barbata* D. Don against colorectal cancer via network pharmacology and experimental verification. *Sci. Rep.* **2025**, *15*, 42779.
80. Yang, P.-W.; Ma, S.-P.; Chen, X.-J.; Yang, F.-M.; Wang, S.-M.; Wang, Q.; Zhang, S.-H. Luteolin suppresses cell migration and invasion via targeting miR-6809-5p/FLOT1/FAK and eliciting EMT in hepatocellular carcinoma. *Transl. Oncol.* **2025**, *61*, 102511.
33. Yue, G.G.; Chan, Y.-Y.; Liu, W.; Gao, S.; Wong, C.-W.; Lee, J.K.; Lau, K.-M.; Lau, C.B. Effectiveness of *Scutellaria barbata* water extract on inhibiting colon tumor growth and metastasis in tumor-bearing mice. *Phytother. Res.* **2021**, *35*, 361–373.
59. Zeng, S.; Chen, L.; Sun, Q.; Zhao, H.; Yang, H.; Ren, S.; Liu, M.; Meng, X.; Xu, H. Scutellarin ameliorates colitis-associated colorectal cancer by suppressing Wnt/β-catenin signaling cascade. *Eur. J. Pharmacol.* **2021**, *906*, 174253.
38. Zhang, L.; Ren, B.; Zhang, J.; Liu, L.; Liu, J.; Jiang, G.; Li, M.; Ding, Y.; Li, W. Anti-tumor effect of *Scutellaria barbata* D. Don extracts on ovarian cancer and its phytochemicals characterisation. *J. Ethnopharmacol.* **2017**, *206*, 184–192.
43. Zhang, L.; Fang, Y.; Feng, J.-Y.; Cai, Q.-Y.; Wei, L.-H.; Lin, S.; Peng, J. Chloroform fraction of *Scutellaria barbata* D. Don inhibits the growth of colorectal cancer cells by activating miR-34a.

Oncol. Rep. **2017**, *37*, 3695–3701.

62. Zhang, J.; Qi, C.; Li, H.; Ding, C.; Wang, L.; Wu, H.; Dai, W.; Wang, C. Exploration of the effect and mechanism of *Scutellaria barbata* D. Don in the treatment of ovarian cancer based on network pharmacology and in vitro experimental verification. *Medicine* **2023**, *102*, e36656.
63. Zhang, X.; Wang, J.; Fu, J.; Hu, J.; Zhang, H.; Ye, M.; Yang, X.; Yu, H.; Xu, H.; Lu, J.; et al. Dissecting the antitumor effects of *Scutellaria barbata*: Initial insights into the metabolism of scutellarin and luteolin by gut microbiota. *J. Pharm. Biomed. Anal.* **2024**, *248*, 116325, Correction in *J. Pharm. Biomed. Anal.* **2024**, *249*, 116393.
34. Zhang, P.; Zhang, T.; Wei, Y.; Yan, J.; Liu, Y.; Bao, Z.; Zeng, X. The antitumor effect of *Scutellaria barbata* D. Don (skullcap) aqueous extract and its mechanism of action in lung adenocarcinoma. *Bol. Latinoam. Caribe Plantas Med. Aromat.* **2025**, *24*, 212–224.
52. Zheng, X.; Kang, W.; Liu, H.; Guo, S. Inhibition effects of total flavonoids from Sculellaria barbata D. Don on human breast carcinoma bone metastasis via downregulating PTHrP pathway. *Int. J. Mol. Med.* **2018**, *41*, 3137–3146.
